# 24th International Symposium on Infections in the Critically Ill Patient

**DOI:** 10.3390/medsci7020023

**Published:** 2019-02-06

**Authors:** Antonio Artigas, Jean Carlet, José Garnacho, Michael Niederman, Antoni Torres

**Affiliations:** 1 Critical Care Center, Sabadell Hospital, University Institute Parc Taulí, Autonomous University of Barcelona, Ciberes, Spain; 2 President of the World Alliance against Antibiotic Resistance (WAAAR), Paris, France; 3 Intensive Care Clinical Unit, Virgen Macarena University Hospital, Seville, Spain; 4 Division of Pulmonary and Critical care Medicine, New York Presbyterian Hospital, Weill Cornell Medical College, USA; 5 Pulmonology Department, Clinic Hospital, University of Barcelona, Barcelona, CIBER Enfermedades Respiratorias, Spain


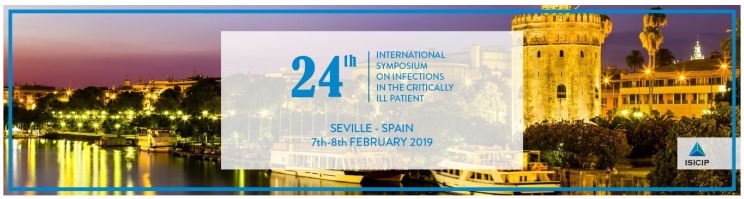


## 1. Introduction

This 24th International Symposium on Infections in the Critically Ill Patient aims to review current concepts, technology and present advances in infections in critically ill patient.

Sepsis, Pulmonary Infections and their therapeutic and preventive strategies will be the topics presented by international experts who will review and update sepsis as a global international problem.

New guidelines of Surviving Sepsis campaign, fluid therapy and vassopressors, a balance view between personalization and protocol treatment and new recommendations for the design of future randomized control trials are provided. The immune response and the emerging methods to personalize sepsis care including new biomarkers and immunomonitoring of patients with sepsis represent a new complementary view to treat patients with severe infections and organ failure in addition to early antibiotic and the control of source of infection.

New ways to treat pulmonary infections including the new global guidelines and the international actions against multiresistant microorganisms and the development of new antibiotics represent key factors to improve the outcome of severe infections.

**José Garnacho, MD** Intensive Care clinical Unit Virgen Macarena University Hospital Seville, Spain**Antonio Artigas, MD** Critical Care Center, Sabadell Hospital University Institute Parc Taulí, Autonomous University of Barcelona, Sabadell, Spain

## 2. Abstracts Speakers

### 2.1. SESSION I. SEPSIS

#### **2.1.1. Year Update and Clinical Review: Sepsis and Septic Shock** 

AnnaneDjillaliGeneral ICU, Raymond Poincaré hospital (AP-HP)Health Science Center Simone VeilSchool of medicine, University of Versailles SQY, University Paris Saclay104 boulevard Raymond Poincaré, 92380 Garches, France
Djillali.annane@aphp.fr


In 2018, there were more than 8000 scientific publications related to sepsis or septic shock, of which about one third were human studies and roughly 120 were randomized trials. 

**What’s new about epidemiology of sepsis?**

Epidemiological and cost data for sepsis were reported from a retrospective analysis of the Brazilian Unified Health System hospitalizations from 2006 to 2015 [1]. Authors used the ICD-10 codes for sepsis. There were a total of 724,458 cases of sepsis out of 115,392,208 hospitalizations. Roughly one out of three were admitted to the ICU. The incidence of sepsis increased by 16 points in ten years from 31.5 in 2006 to 47.4 per 100,000 in 2015. Likewise, in the same interval, mortality increased from about 14% to 30%, with major differences between public and private hospitals with higher mortality rates in the public institutions. 

In a prospective cohort of intensive care unit (ICU) patients in an academic centre in The Netherland, of 632 patients with sepsis, about three quarters met Sepsis 2 definition for septic shock and one out of two met Sepsis 3 criteria [2]. Mortality rates were five points higher in those meeting the Sepsis 3 criteria compared to those meeting Sepsis 2 criteria. These findings confirmed that using Sepsis 3 definition will likely result in a lower incidence of sepsis than with the previous definition, and higher mortality risk. 

**What’s new about the diagnosis of sepsis?**

A recent prospective observational study has investigated the clinical utility of SeptiCyte LAB test for the discrimination of ICU patients with vs. without sepsis related systemic inflammation [3]. SeptiCyte LAB. is a real-time quantitative polymerase chain reaction (RT-qPCR) assay that quantitates the relative expression levels of four genes (CEACAM4, LAMP1, PLAC8, PLA2G7) and that was cleared in 2017 by the United States Food and Drug Administration (FDA) for the diagnosis of sepsis. The study combined information from three cohorts (two from the United States and one from The Netherland). The SeptiScores organized into four numerical bands (band 1, 0–3.0; band 2, 3.1–4.4; band 3, 4.5–5.9; band 4, 6–10), showed better performance for the diagnosis of sepsis than any reference test with an area under the curve for the receiver operating characteristic (ROC) ranging from 0.82 to 0.89. 

The recent winner of the first Pioneers tournament, involving innovators from more than 100 countries and encompassing all fields in science, has developed a synthetic polymer capable of binding toxins from bacteria that may serve both as rapid test for sepsis and of therapeutic removal of endotoxin [4].

**What’s new about the management of sepsis?**

The two largest randomized trials of corticosteroids for septic shock have been published in 2018, i.e., ADRENAL [5] and APROCCHSS [6]. Both trials found that corticosteroids accelerate the recovery from shock and organs dysfunction in sepsis, reduce length of stay in the hospital and are rather well tolerated. APROCCHSS trial also found significant survival benefit, about 6% absolute reduction in short- and long-term mortality rates. ADRENAL found a non-significant reduction in mortality. However, when one combine the two trials (*n* = 4922 participants), the relative risk of death was 0.90 (95% CI (confident interval): 0.82 to 0.98) in favour of corticosteroids (*p* value = 0.02) without heterogeneity in the results (I^2^ = 0%) [7]. Interestingly, molecular analysis of a large cohort of patients with septic shock identified a transcriptomic sepsis response signature that predicted harm from hydrocortisone [8]. 

Artificial intelligence (AI) may assist physicians in the management of sepsis. In a recent analysis of two large electronic ICU databases (MIMIC II and Philips eICU database) based on reinforcement learning modelling, researchers elaborated algorithms to guide fluid resuscitation and vasopressor therapy in septic shock [9]. They demonstrated that AI assisted decisions prevented misuses of fluids and vasopressors and may reduce mortality from sepsis.

References

1. Quintano Neira, R.A.; Hamacher, S., Japiassú, A.M. Epidemiology of sepsis in Brazil: Incidence, lethality, costs, and other indicators for Brazilian Unified Health System hospitalizations from 2006 to 2015. *PLoS ONE*
**2018**
*13* e0195873.
2. Driessen, R.G.H.; van de Poll, M.C.G.; Mol, M.F.; van Mook, W.N.K.A.; Schnabel, R.M. The influence of a change in septic shock definitions on intensive care epidemiology and outcome: Comparison of sepsis-2 and sepsis-3 definitions. *J. Infect. Dis*. **2018**, *50*, 207–213.
3. Miller, R.R. 3rd; Lopansri, B.K.; Burke, J.P.; Levy, M; Opal, S; Rothman, R.E.; D’Alessio, F.R.; Sidhaye, V.K.; Aggarwal, N.R.; Balk, R; et al. Validation of a Host Response Assay, SeptiCyte LAB, for Discriminating Sepsis from Systemic Inflammatory Response Syndrome in the ICU. *Am J Respir Crit Care Med*. **2018**, *198*, 903–913.
4. Allen, D. Can a polymer help reduce deaths from sepsis? MD+DI Qmed; 2019.
5. https://www.mddionline.com/can-polymer-help-reduce-deaths-sepsis
6. Venkatesh, B.; Finfer, S; Cohen, J.; Rajbhandari, D.; Arabi, Y.; Bellomo, R.; Billot, L.; Correa, M.; Glass, P.; Harward, M. et al. Adjunctive Glucocorticoid Therapy in Patients with Septic Shock. *N. Engl. J. Med*. **2018,**
*378*, 797–808.
7. Annane, D.; Renault, A.; Brun-Buisson, C.; Megarbane, B.; Quenot, J.P.; Siami, S.; Cariou, A.; Forceville, X.; Schwebel, C.; Martin, C.; et al. CRICS-TRIGGERSEP Network. Hydrocortisone plus Fludrocortisone for Adults with Septic Shock. *N. Engl. J. Med*. **2018**, *378*, 809–18.
8. Rochwerg, B.; Oczkowski, S.J.; Siemieniuk, R.A.C.; Agoritsas, T.; Belley-Cote, E.; D’Aragon, F.; Duan, E.; English, S.; Gossack-Keenan, K.; Alghuroba, M.; et al. Corticosteroids in Sepsis: An Updated Systematic Review and Meta-Analysis. *Crit. Care Med*. **2018**, 46, 1411–1420.
9. Antcliffe, D.B.; Burnham, K.L.; Al-Beidh, F.; Santhakumaran, S.; Brett, S.J.; Hinds, C.J.; Ashby, D.; Knight, J.C.; Gordon, A.C. Transcriptomic Signatures in Sepsis and a Differential Response to Steroids: From the VANISH Randomized Trial. *J. Respir. Crit. Care Med*. **2018**. doi:10.1164/rccm.201807-1419OC.

#### **2.1.2. Elderly Patients with Sepsis** 

GuidetBertrandSorbonne Universités, INSERM, UMR_S 1136, Institut Pierre Louis d’Epidémiologie et de Santé Publique, F-75012, Paris, FranceAssistance Publique - Hôpitaux de Paris, Hôpital Saint-Antoine, service de réanimation médicale, Paris, F-75012, France

**Abstract**

Sepsis in the elderly population is a global health concern with severe consequences to both the patient as an individual (high mortality rate) and the health care system as a whole (high annual costs).

**Epidemiology**

The reported incidence of sepsis is increasing, likely reflecting aging population with more comorbidities. Almost 60% of septic patients are over 65 years of age [1]. Sepsis continues to grow as a problem. Martin et al. reported that over a 22-year period, the incidence of sepsis in the United States had almost quadrupled—from estimates of around 164,000 cases per year in the 1970s to a little fewer than 660,000 cases per year at the turn of the millennium [2]. Furthermore, institutionalization, invasive devices, multiple medications, reduced renal function and poor nutritional status contribute to the high risk of sepsis. The number of ICU admission related to respiratory infections has dramatically increased in the elderly in the past 10 years [3]. 

**Clinical presentation of sepsis and/or septic shock**

Subtle or atypical clinical presentations induce delays in diagnosis and thus appropriate treatment. Septic elderly patients often exhibit no fever, tachycardia and capillary dilatation carrying a diagnostic challenge for early recognition. The most common example is the presence of altered mental status, which is a nonspecific marker of infection in older patients and does not necessarily indicate a nervous system infection. Other examples of unique symptoms of infection in older patients include: lethargy, tachypnea, loss of appetite, dehydration, weakness, dizziness, falls, and incontinence [4]

**Ageing and the immune system:**

Immunosenescence refers to a gradual age dependent deterioration of the immune system brought on by ageing process. This can lead to deterioration in the ability to respond properly to infections. Immunosenescence has been described as the result of a chronic hyperstimulation of the immune system components [5]. This affects cell-mediated immunity and humoral immune responses in particular. Innate immunity is generally thought to be relatively well preserved or enhanced during aging compared with adaptive immunity, which is characterized by several alterations [6]. All immunocompetent cells are affected. Macrophages show decreased ability to phagocytosis and increased cytokine production. There is a decreased production of B-cell lymphocytes and in T-lymphocytes CD8 cells increases while CD4 decreases. The neutrophils have decreased chemotaxis and free radical production while the NK cells show decreased cytotoxicity and IL-2 production. Immunosenescence explains the higher incidence of sepsis and also higher severity in elderly patients compared to younger patients. Sepsis and stress share many features in elderly patients [7].

Elderly patients are unable to mount the inflammatory response that characterizes an infection. They typically exhibit compensatory anti-inflammatory response syndrome (CARS) characterized by cutaneous anergy, reduction of lymphocytes by means of apoptosis, decreased cytokine response of monocytes to stimulation, decreased numbers of human leukocyte antigen (HLA) antigen-presenting receptors on monocytes, and decreased expression of cytokines, such as IL-10, that suppress tumor necrosis factor (TNF) expression [8]. 

**Treatment**

Percutaneous and less invasive procedures are recommended in elderly fragile patients [9]. Elderly patients are exposed to numerous repeated antimicrobial therapies, this is partially due to comorbid illnesses, immunocompromised states, residence in nursing homes, repeated hospitalizations and the increased prevalence of intra-abdominal, soft-tissue and urinary tract infections. This significantly increases the risk of older patients to accumulate multidrug-resistant microbial flora, necessitating an even broader spectrum of empirical antibiotics. Additionally, septic patients may exhibit pronounced myocardial dysfunction consequently a rate of fluid of 10 mL/kg within the first hour of resuscitation seems reasonable in order to avoid fluid overload [10]. 

**Prognosis**

Increasing age, independent of severity of illness and presence of comorbid conditions, is associated with a higher rate of death due to sepsis [1,2]. However, this association is not uniformly observed as confounding factors are contributory. In a single center study of septic intensive care unit (ICU) patients, the hospital mortality was significantly higher in very elderly compared to elderly patients (54.2% vs. 47.4%; *p* = 0.02). Among the very elderly patients, prompt adherence of the resuscitation bundle was predictor of hospital mortality [11].

Besides crude mortality, qualitative outcomes are very important to consider with decline of functional autonomy and health related quality of life. Especially in the elderly, surviving sepsis has been associated with a significant increased risk of consequent significant persistent cognitive impairment and functional disability. Iwashyna et al, in a prospective cohort study of 1194 patients hospitalized for severe sepsis, described an increased incidence of long-term cognitive impairment and functional limitations in survivors of sepsis as compared to non-sepsis hospitalized patients [12]. Cognitive impairment and functional limitations persisted for a minimum of eight years in sepsis survivors. 

**Conclusions**

Sepsis in the elderly is a growing concern as the population shifts towards an older demographic. In order to best manage sepsis, it is important to identify the groups most at risk and develop management protocols to effectively minimize mortality. The specificities of elderly patients with underlying diseases, immunosenescence and loss of physiological reserve resulting in frailty phenotype deserve special attention. Guidelines should probably be tailored to this specific population since the bundle may not applicable directly to elderly patients.

References

1. Angus, D.C.; Linde-Zwirble, W.T.; Lidicker, J.; Clermont, G.; Carcillo, J; Pinsky, M.R. Epidemiology of severe sepsis in the United States: analysis of incidence, outcome, and associated costs of care. *Crit. Care Med.*
**2001**, *29*, 1303–1310.
2. Martin, G.S.; Mannino, D.M.; Eaton, S.; Moss, M. The epidemiology of sepsis in the United States from 1979 through 2000. *N. Engl. J. Med*. **2003**, *348*, 1546–1554.
3. Laporte, L.; Hermertet, C.; Jouan, Y.; Gaborit, C.; Rouve, E.; Shea, K.M.; Si-Tahar, M.; Dequin, P.F.; Grammatico-Guillon, L.; Guillon, A. Ten-year trends in intensive care admissions for respiratory infections in the elderly. *Ann. Intensive Care*
**2018**, *8*, 84.
4. Clifford, K.M.; Dy-Boarman, E.A.; Haase, K.K.; Maxvill, K.H.; Pass, S.; Alvarez, C.A. Challenges with Diagnosing and Managing Sepsis in Older Adults. *Expert Rev. Anti. Infect. Ther*. **2016**, 14, 231–241.
5. Franceschi, C.; Bonafe, M.; Valensin, S.; Olivieri, F.; De Luca, M.; Ottaviani, E.; De Benedictis, G. Inflamm-aging. An evolutionary perspective on immunosenescence. *Ann. N. Y. Acad. Sci*. **2000**, *908*, 244–254.
6. Martín, S., Pérez, A., Aldecoa, C. Sepsis and Immunosenescence in the Elderly Patient: A Review. *Front. Med*. **2017**, *4*, 20.
7. Fali, T.; Vallet, H.; Sauce, D. Impact of stress on aged immune system compartments: overview from fundamental to clinical data. Experimental Gerontology. *Exp. Gerontol*. **2018**, *105*, 19–26. https://doi.org/10.1016/j.exger.2018.02.007.
8. Ward, N.S.; Casserly, B.; Ayala, A. The compensatory anti-inflammatory response syndrome (CARS) in critically ill patients. *Clin. Chest Med*. **2008**, *29*, 617–625.
9. Martínez, M.L.; Ferrer, R.; Torrents, E.; Guillamat-Prats, R.; Gomà, G.; Suárez, D.; Álvarez-Rocha, L.; Pozo Laderas, J.C.; Martín-Loeches, I.; Levy, M.M.; et al. Edusepsis Study Group: Impact of Source Control in Patients With Severe Sepsis and Septic Shock. *Crit. Care Med*. **2017**, *45*, 11–19.
10. Marik, P.E.; Linde-Zwirble, W.T.; Bittner, E.A.; Sahatjian, J.; Hansell, D. Fluid administration in severe sepsis and septic shock, patterns and outcomes: an analysis of a large national database. *Intensive Care Med*. **2017**, *43*, 625–632.
11. Martín-Loeches, I.; Guia, M.C, Vallecoccia, M.S.; et al. Risk factors for mortality in elderly and very elderly critically ill patients with sepsis: A prospective, observational, multicenter cohort study. *Ann. Intensive Care* (in press).
12. Iwashyna, T.J.; Ely, E.W.; Smith, D.M.; Langa, K.M. Long-term cognitive impairment and functional disability among survivors of severe sepsis. *JAMA*
**2010**, *304*, 1787–1794.

#### **2.1.3. Negative Clinical Trials: a Revisited Design** 

RussellJames A.Professor of Medicine, University of British Columbia, Principal Investigator, Centre for Heart Lung Innovation, Vancouver, BC, Canada

Sepsis is a very heterogeneous condition and there have been no drugs approved for sepsis save for the transient use of activated protein C. Nearly all randomized controlled trials (RCTs) in sepsis and septic shock are negative so we MUST change our paradigm to improve patient outcomes and mitigate increasing health care costs.

I suggest a **BETTER** design—**B**iomarker-guided **E**arly an**T**ibiotic adjuvan**T**s in **ER** (**BETTER**). Table 1 summarizes key features of **BETTER** RCTs in sepsis and septic shock compared to oncology trials that lead to **BETTER** outcomes.

**Biomarker-guided** means using *predictive* biomarkers that identify patients who have an improved response to therapy such as genomic markers for improved response to vasopressin [1], norepinephrine [2], angiotensin-II [3], corticosteroids [4,5], PCSK9 inhibition [6,7] and CETP inhibition [8,9] and weaning from mechanical ventilation [10]. More specifically, the half-life of vasopressin in human plasma is 4–24 min and is primarily determined by leucyl/cystinyl aminopeptidase (LNPEP; also known as vasopressinase), a physiologically essential enzyme that cleaves peptide bonds of vasopressin. The genetic variation in *LNPEP* (vasopressinase) is associated with 28-day mortality in septic shock and is associated with biological effect on vasopressin clearance and serum sodium regulation. Regarding norepinephrine, the β2-adrenergic receptor gene (*ADRB2*) plays a key role in outcome and response to adrenergic agonists in cardiovascular diseases. We found that the AA genotype of the β2-adrenergic receptor gene rs1042717 G/A polymorphism, marking the known functional ADRB2 *CysGlyGln* haplotype, was significantly associated with increased mortality and more organ dysfunction in two cohorts of septic shock patients. These results are consistent with the observation that the AA genotype is associated with decreased responsiveness to the anti-inflammatory effects of adrenergic agonists. Regarding weaning, an RCT compared two methods of weaning from ventilation: (1) weaning guided by fluid management to progressively decrease B-type natriuretic peptide (BNP, also known as NPPB) using diuretics (i.e., a biomarker-guided protocol) versus (2) weaning and fluid management by usual clinical guidelines. The BNP group weaned more quickly (time to extubation was decreased from about 59 to about 42 h) and had more ventilator-free days [10]. 

This Biomarker-guided strategy is the standard of care and has improved outcomes in oncology (e.g., standard of care in breast cancer: Her 2 neu expression defines who gets treatment with Herceptin).

**Early** means early in time and early in disease evolution. Time: Vasopressin infusion is more effective when used within 12 h; disease evolution: vasopressin is also more effective in patients who have less severe septic shock (norepinephrine dose < 15 μg/min or lactate < 2 mmol/L) [11,12] perhaps in part because vasopressin prevents progression of inflammation [13] and organ failure. Early goal-directed therapy (EGDT) was not effective overall in three recent RCTs [14] but may have been effective in patients who had lower levels of inflammatory and coagulation biomarkers [15]. We and others have noted that several therapies are more effective when given to patients who are less severely ill, perhaps because their condition is more reversible and the inflammatory, coagulation, and apoptosis cascades are not as briskly stimulated then [16].

**anTibiotic adjuvanTs** means supplementing the bacterial killing effects of antibiotics by augmenting the host’s natural lipopolysaccharide (LPS) and lipotechoic acid (LTA) clearance mechanisms. Early antibiotics improve mortality of septic shock [17–19] but do not remove LPS or LTA directly. Microbial cell walls contain pathogenic lipids including LPS in Gram-negative bacteria, lipoteichoic acid in Gram-positive bacteria, and phospholipomannan in fungi. These pathogen lipids are major ligands for innate immune receptors that trigger the septic inflammatory response. Alternatively, the host has a defense mechanism: pathogen lipids can be cleared and neutralized, thereby mitigating the inflammatory response. Pathogen lipids released into the circulation are initially bound by transfer proteins, notably LPS binding protein (LBP) and phospholipid transfer protein (PLTP), and incorporated into high density lipoprotein (HDL) particles. LBP, PLTP, and other transfer proteins transfer these lipids to ApoB-containing lipoproteins, including low density (LDL) and very low density (VLDL) lipoproteins and chylomicrons. LDL and VLDL LPS and LTA are cleared by the LDL and VLDL receptors in liver and adipose tissue respectively. LDL receptor density is decreased by proprotein convertase subtilisin/kexin type 9 (PCSK9) and PCSK9 inhibition increases LDL receptor density and decreases LPS, LTA and cytokine levels as well as mortality in human genomic (in patients who carry PCSK9 loss-of-function variants) and animal model studies [7,20]. Low HDL levels are associated with increased mortality [21] and subjects who have cholesterylester transfer protein (CETP loss-of-function variants have higher HDL levels than wild type subjects. Human genomic (CETP loss-of-function variants) and animal models show that increasing HDL by CETP inhibition improves outcomes [8,9] of septic shock. In future studies, the integration of different genomic methods and application of multiple ‘omics approaches (e.g., genomics, proteomics, metabolomics, transcriptomics, and epigenomics) could lead to discovery and validation of accurate biomarkers that may predict risk, response to therapy, mortality and more novel drugs for septic shock. We have utilized a novel inverted drug discovery strategy that incorporates (1) focus on the early infectious stage of sepsis, (2) multiple ‘omics (multi-‘omics) and (3) we start with human ‘omics for drug candidate discovery (instead of animal models), confirm mechanisms in human cell and clinically-relevant sepsis models, and only then make go/no-go decisions for potential validated targets for clinical development—all this designed to increase the chances of discovering effective sepsis drugs.

In **ER** means that RCTs must recruit septic patients in the ER and not wait until patients are in the ICU so that biomarkers can be measured early and personalized care can be initiated early. Three RCTs of EGDT were coordinated across continents and countries [22] so that results could be compared and a patient level meta-analysis [14] could be done—all this was planned prospectively and emphasized ER recruitment of patients into these complex RCTs. These RCTs highlighted the successful partnerships of ER and ICU in RCTs that recruited patients with septic shock within 1–2 h of presentation [14,23–25]. Another ER study showed that early antibiotics in ER are the most effective component of early ER interventions [19].

References

1. Nakada TA, Russell JA, Wellman H, Boyd JH, Nakada E, Thain KR, et al. Leucyl/cystinyl aminopeptidase gene variants in septic shock. *Chest*
**2011**, *139*, 1042–1049.
2. Nakada TA, Russell JA, Boyd JH, Aguirre-Hernandez R, Thain KR, Thair SA, et al. Beta2-Adrenergic receptor gene polymorphism is associated with mortality in septic shock. *Am. J. Respir. Crit. Care Med*. **2010**, *181*, 143–9.
3. Nakada TA, Russell JA, Boyd JH, McLaughlin L, Nakada E, Thair SA, et al. Association of angiotensin II type 1 receptor-associated protein gene polymorphism with increased mortality in septic shock. *Crit. Care Med*. **2011**, *39*, 1641–1648.
4. Bentzer P, Fjell C, Walley KR, Boyd J, Russell JA. Plasma cytokine levels predict response to corticosteroids in septic shock. *Intensive Care Med*. **2016**, *42*, 1970–1979.
5. Russell JA. When and how to use predictive biomarkers for corticosteroid treatment of septic shock. *Crit. Care*
**2018**, *22*, 318.
6. Boyd JH, Fjell CD, Russell JA, Sirounis D, Cirstea MS, Walley KR. Increased Plasma PCSK9 Levels Are Associated with Reduced Endotoxin Clearance and the Development of Acute Organ Failures during Sepsis. *J. Innate Immun*. **2016**, *8*, 211–220.
7. Walley KR, Thain KR, Russell JA, Reilly MP, Meyer NJ, Ferguson JF, et al. PCSK9 is a critical regulator of the innate immune response and septic shock outcome. *Sci. Transl. Med*. **2014**, *6*, 258ra143.
8. Genga KR, Trinder M, Kong HJ, Li X, Leung AKK, Shimada T, et al. CETP genetic variant rs1800777 (allele A) is associated with abnormally low HDL-C levels and increased risk of AKI during sepsis. *Sci. Rep*. **2018**, *8*, 16764.
9. Trinder M, Genga KR, Kong HJ, Blauw LL, Lo C, Li X, et al. Cholesteryl Ester Transfer Protein Influences High-Density Lipoprotein Levels and Survival in Sepsis. *Am. J. Respir. Crit. Care Med*. **2018**. https://doi.org/10.1164/rccm.201806-1157OC.
10. Russell JA. Biomarker (BNP)-guided weaning from mechanical ventilation: time for a paradigm shift? *Am. J. Respir. Crit. Care Med*. **2012**, *186*, 1202–1204.
11. Russell JA, Lee T, Singer J, Boyd JH, Walley KR, Vasopressin, et al. The Septic Shock 3.0 Definition and Trials: A Vasopressin and Septic Shock Trial Experience. *Crit. Care Med*. **2017**, *45*, 940–948.
12. Russell JA, Walley KR, Singer J, Gordon AC, Hebert PC, Cooper DJ, et al. Vasopressin versus norepinephrine infusion in patients with septic shock. *N. Engl. J. Med*. **2008**, *358*, 877–887.
13. Russell JA, Fjell C, Hsu JL, Lee T, Boyd J, Thair S, et al. Vasopressin compared with norepinephrine augments the decline of plasma cytokine levels in septic shock. *Am. J. Respir. Crit. Care Med*. **2013**, *188*, 356–364.
14. Angus DC. Early, Goal-Directed Therapy for Septic Shock - A Patient-Level Meta-Analysis. *N. Engl. J. Med*. **2017**, *377*, 995.
15. Kellum JA, Pike F, Yealy DM, Huang DT, Shapiro NI, Angus DC, et al. Relationship Between Alternative Resuscitation Strategies, Host Response and Injury Biomarkers, and Outcome in Septic Shock: Analysis of the Protocol-Based Care for Early Septic Shock Study. *Crit. Care Med*. **2017**, *45*, 438–445.
16. Thompson BT. Greater Treatment Effect With Lower Disease Severity: VASST Insights. *Crit. Care Med*. **2017**, *45*, 1094–1095.
17. Liu VX, Fielding-Singh V, Greene JD, Baker JM, Iwashyna TJ, Bhattacharya J, et al. The Timing of Early Antibiotics and Hospital Mortality in Sepsis. *Am. J. Respir. Crit. Care Med*. **2017**, *196*, 856–863.
18. Kumar A, Roberts D, Wood KE, Light B, Parrillo JE, Sharma S, et al. Duration of hypotension before initiation of effective antimicrobial therapy is the critical determinant of survival in human septic shock. *Crit. Care Med.*
**2006,**
*34*, 1589–1596.
19. Seymour CW, Gesten F, Prescott HC, Friedrich ME, Iwashyna TJ, Phillips GS, et al. Time to Treatment and Mortality during Mandated Emergency Care for Sepsis. *N. Engl. J. Med*. **2017**, *376*, 2235–2244.
20. Topchiy E, Cirstea M, Kong HJ, Boyd JH, Wang Y, Russell JA, et al. Lipopolysaccharide Is Cleared from the Circulation by Hepatocytes via the Low Density Lipoprotein Receptor. *PLoS ONE*. **2016**, *11*, e0155030.
21. Cirstea M, Walley KR, Russell JA, Brunham LR, Genga KR, Boyd JH. Decreased high-density lipoprotein cholesterol level is an early prognostic marker for organ dysfunction and death in patients with suspected sepsis. *J. Crit. Care*. **2017**, *38*, 289–294.
22. Russell JA, Moller MH, Annane D. Early goal-directed therapy: from discovery through enthusiasm to equipoise? *Intensive Care Med*. **2015**, *41*, 1676–1678.
23. Angus DC, Yealy DM, Kellum JA, Pro CI. Protocol-based care for early septic shock. *N. Engl. J. Med*. **2014**, *371*, 386.
24. Mouncey PR, Power GS, Coats TJ. Early, Goal-Directed Resuscitation for Septic Shock. *N. Engl. J. Med*. **2015**, *373*, 577–578.
25. Pro CI, Yealy DM, Kellum JA, Huang DT, Barnato AE, Weissfeld LA, et al. A randomized trial of protocol-based care for early septic shock. *N. Engl. J. Med*. **2014**, *370*, 1683–1693.

### 2.2. SESSION II. FLUID THERAPY AND VASSOPRESSORS

#### **2.2.1. New Vasoactive Agents** 

RussellJames A.Professor of Medicine, University of British Columbia, Principal Investigator, Centre for Heart Lung Innovation, Vancouver, BC, Canada

The Surviving Sepsis Guidelines [1] recommend norepinephrine (NE) as the first vasopressor agent followed by epinephrine (EPI) or vasopressin (AVP) in patients who do not respond adequately to norepinephrine. Dobutamine is added in patients who have evidence of depressed ventricular function due to underlying disease or septic cardiac dysfunction.

I review several new studies regarding these agents and several new agents that were not successful in pivotal RCTs (levosimendan), were recently approved (angiotensin-II (AT=II)) or are in development (selepressin).

**Norepinephrine**

The ARISE [2] early Goal-Directed Therapy (EGDT) RCT did a sub-study of vasopressor use; the median time from ED presentation to commencing a vasopressor was 4.4 (2.7, 7.1) h (38% prior to central venous access) after receiving 3.1 (2.3, 4.3) L. intravenous fluid prior [3]. Interestingly, earlier initiation of vasopressor(s) was associated with higher crude 90-day mortality. 

In patients who are on both NE and AVP, tapering NE before AVP may be associated with a higher incidence of hypotension [4]. Clinically, NE is dosed by weight or not by weight. A retrospective study found that morbidly obese patients had lower in-hospital mortality, but had higher 1-year mortality compared to normal- and under-weight patients. Cumulative norepinephrine exposure was highest in morbidly obese patients [5]. Total norepinephrine exposure was an independent mortality predictor in septic shock.

**Vasopressin**

The Vasopressin and Septic Shock Trial (VASST) found no difference in overall mortality, but decreased mortality in the AVP group who had less severe septic shock [6]. It is not clear whether AVP versus NE changed mortality in practice in the VASST coordinating center hospital after VASST was published. We used propensity matching of AVP- to NE-treated patients in the VASST coordinating center before and after VASST was published [7]. Before VASST, AVP was associated with increased mortality compared to NE while after VASST, there was no difference in mortality between AVP- and NE-treated patients. 

Acute Kidney Injury (AKI) sub-phenotypes could be used to identify responsiveness to AVP in septic shock. Latent class analysis methodology was applied independently in two critically ill populations (Discovery: *n* = 794 and Replication: *n* = 425) with AKI. In VASST, AVP compared to NE was associated with improved 90-day mortality in AKI-SP1 (27% vs. 46%), but no significant difference in AKI-SP2 (45% vs. 49%) [8]. This analysis identified two molecularly distinct AKI sub-phenotypes with different response to AVP. 

Some patients with septic shock are on chronic renin-angiotensin-aldosterone system inhibitor but it is unknown whether the hemodynamic response to AVP differs between patients who are on vs. not on chronic renin-angiotensin-aldosterone system inhibitor(s) [9]. There was no significant difference in 6-hour mean arterial pressure in septic shock patients receiving vasopressin who were on versus those not on chronic renin-angiotensin-aldosterone system inhibitor therapy. Renin-angiotensin-aldosterone system inhibitor patients had lower total vasopressor requirements at 24 h compared with non-renin-angiotensin-aldosterone system inhibitor patients.

The Septic Shock 3.0 definition could alter treatment comparisons in RCTs. We wondered whether the AVP versus NE comparison of 28-day mortality of patients who met the Septic Shock 3.0 definition (lactate > 2 mmol/L) differed from AVP versus NE in the (pre-Sepsis 3.0) septic shock definition used in VASST [10]. In VASST, the Septic Shock 3.0 definition decreased sample size by half and increased 28-day mortality rate by 10%. AVP lowered mortality versus NE if lactate was less than or equal to 2 mmol/L. Patients had higher plasma cytokines in lactate greater than 2 vs. less than or equal to 2 mmol/L, indicating a brisker cytokine response to infection. The Septic Shock 3.0 definition and our findings have important implications for RCT design in septic shock.

**Levosimendan**

Levosimendan was a possible alternative to dobutamine in septic shock with depressed ventricular function because levosimendan is a calcium-sensitising drug with inotropic and other properties that could be beneficial. In a large pivotal RCT there was no difference in SOFA score between the levosimendan group (6.7, SD 4.0) versus placebo or in 28-day mortality rates (34.5% and 30.9%) [11]. Levosimendan was associated with lower likelihood of successful extubation and increased risk of supraventricular tachyarrhythmias. This RT was critiqued because there was no need for a baseline echocardiogram to detect decreased ventricular function for inclusion into the RCT. However, levosimendan is not recommended for septic shock with depressed ventricular function.

**Angiotensin-II**

AT-II is a potent vasoconstrictor that could be beneficial in refractory septic shock and a proof-of-principle RCT showed some benefit on short term markers (marked reduction in NE dose requirements) [12]. A large RCT found that angiotensin II increased MAP more rapidly (within 3 h) than did NE [13] but there were no results regarding effects on non-cardiovascular organ dysfunction. Mortality was nominally but not significantly lower with angiotensin II than NE (46% vs. 54%, *p* = 0.12). Angiotensin II is available now clinically and may be effective for profound vasodilatory shock.

**Selepressin**

Selepressin is a highly selective V1a agonist that could have advantages over AVP because selepressin would mitigate effects on vasopressin-induced increased von Willbrand multimers (procoagulant proteins) and could mitigate increased permeability and fluid balance in septic shock [14]. Selepressin increased MAP and quite dramatically minimized the increase in fluid balance in an ovine model of septic shock [15]. In a proof-of-principle RCT showed that a greater fraction of patients on 2.5 g/kg/min selepressin maintained MAP >60 mmHg without norepinephrine (about 50% and 70% at 12 and 24 h, respectively) vs. 1.25 ng/kg/minute selepressin and placebo and NE was weaned more rapidly with selepressin 2.5 ng/kg/min vs. placebo (0.04 vs. 0.18 μg/kg/minute at 24 h) [16]. 

Furthermore, fluid balance was lower from day 5 on with selepressin 2.5 ng/kg/min vs. placebo possibly due to protection of endothelial permeability. The results of a large novel pivotal RCT that uses (1) response adaptive design and (2) organ dysfunction as the primary endpoint of selepressin in septic shock will be reported shortly [17].

References

1. Rhodes A, Evans LE, Alhazzani W, Levy MM, Antonelli M, Ferrer R, et al. Surviving Sepsis Campaign: International Guidelines for Management of Sepsis and Septic Shock: 2016. *Crit. Care Med*. **2017**, *45*, 486–552.
2. Investigators A, Group ACT, Peake SL, Delaney A, Bailey M, Bellomo R, et al. Goal-directed resuscitation for patients with early septic shock. *N. Engl. J. Med*. **2014**, *371*, 1496–506.
3. Udy AA, Finnis M, Jones D, Delaney A, Macdonald S, Bellomo R, et al. Incidence, Patient Characteristics, Mode of Drug Delivery, and Outcomes of Septic Shock Patients Treated with Vasopressors in the Arise Trial. *Shock*
**2018**. doi:10.1097/SHK.0000000000001281.
4. Jeon K, Song JU, Chung CR, Yang JH, Suh GY. Incidence of hypotension according to the discontinuation order of vasopressors in the management of septic shock: A prospective randomized trial (DOVSS). *Crit. Care*
**2018**, *22*, 131.
5. Kotecha AA, Vallabhajosyula S, Apala DR, Frazee E, Iyer VN. Clinical Outcomes of Weight-Based Norepinephrine Dosing in Underweight and Morbidly Obese Patients: A Propensity-Matched Analysis. *J. Intensive Care Med*. **2018**. https://doi.org/10.1177/0885066618768180.
6. Russell JA, Walley KR, Singer J, Gordon AC, Hebert PC, Cooper DJ, et al. Vasopressin versus norepinephrine infusion in patients with septic shock. *N. Engl. J. Med*. **2008**, *358*, 877–887.
7. Russell JA, Wellman H, Walley KR. Vasopressin versus norepinephrine in septic shock: a propensity score matched efficiency retrospective cohort study in the VASST coordinating center hospital. *J. Intensive Care*
**2018**, *6*, 73.
8. Bhatraju PK Zl, Herting J, Katz R, Mikacenic C, Kosamo S, Morrell ED, Robinson-Cohen C, Calfee CS, Christie JD, Liu KD, et al. Identification of Acute Kidney Injury sub-phenotypes with differing molecular signatures and response to vasopressin therapy. *Am. J. Respir. Crit. Care Med*. **2018**. doi:10.1164/rccm.201807-1346OC.
9. Erwin BL, Denaburg MA, Barker AB, McArdle PJ, Windham ST, Morgan CJ. Evaluation of Vasopressin for Septic Shock in Patients on Chronic Renin-Angiotensin-Aldosterone System Inhibitors. *Crit. Care Med*. **2017**, *45*, e1226-e32.
10. Russell JA, Lee T, Singer J, Boyd JH, Walley KR, Vasopressin, et al. The Septic Shock 3.0 Definition and Trials: A Vasopressin and Septic Shock Trial Experience. *Crit. Care Med*. **2017**, *45*, 940–948.
11. Gordon AC, Perkins GD, Singer M, McAuley DF, Orme RM, Santhakumaran S, et al. Levosimendan for the Prevention of Acute Organ Dysfunction in Sepsis. *N. Engl. J. Med*. **2016**, *375*, 1638–1648.
12. Chawla LS, Busse L, Brasha-Mitchell E, Davison D, Honiq J, Alotaibi Z, et al. Intravenous angiotensin II for the treatment of high-output shock (ATHOS trial): a pilot study. *Crit. Care*. **2014**, *18*, 534.
13. Khanna A, English SW, Wang XS, Ham K, Tumlin J, Szerlip H, et al. Angiotensin II for the Treatment of Vasodilatory Shock. *N. Engl. J. Med*. **2017**, *377*, 419–430.
14. Marks JA, Pascual JL. Selepressin in septic shock: sharpening the VASST effects of vasopressin? *Crit. Care Med*. **2014**, *42*, 1747–1748.
15. He X, Su F, Taccone FS, Laporte R, Kjolbye AL, Zhang J, et al. A Selective V(1A) Receptor Agonist, Selepressin, Is Superior to Arginine Vasopressin and to Norepinephrine in Ovine Septic Shock. *Crit. Care Med*. **2016**, *44*, 23–31.
16. Russell JA, Vincent JL, Kjolbye AL, Olsson H, Blemings A, Spapen H, et al. Selepressin, a novel selective vasopressin V1A agonist, is an effective substitute for norepinephrine in a phase IIa randomized, placebo-controlled trial in septic shock patients. *Crit. Care*
**2017**, *21*, 213.
17. Lewis RJ, Angus DC, Laterre PF, Kjolbye AL, van der Meulen E, Blemings A, et al. Rationale and Design of an Adaptive Phase 2b/3 Clinical Trial of Selepressin for Adults in Septic Shock. Selepressin Evaluation Programme for Sepsis-induced Shock-Adaptive Clinical Trial. *Ann. Am. Thorac. Soc.*
**2018,**
*15*, 250–257.

#### **2.2.2. Balanced Crystalloids versus Saline in Sepsis** 

KellumJohn A.University of Pittsburgh, Pittsburgh, Pennsylvania, USA

Administration of intravenous fluids is a ubiquitous therapy in medicine. Selection of intravenous fluids is usually based on clinician’s preferences with marked regional variations. “Normal” saline (0.9% sodium chloride), the most commonly prescribed crystalloid solution worldwide, contains supraphysiologic concentrations of chloride (154 mmol/L), with a strong ion difference (SID) of zero. Balanced or “buffered” solutions, on the other hand, have a lower sodium and chloride content and a positive SID, with an electrochemical composition that more closely approximates extracellular fluid. 

In experimental studies, resuscitation with 0.9% saline, but not with low-chloride solutions, led to hyperchloremic metabolic acidosis, increased inflammatory response, coagulopathy, derangements in renal perfusion and acute kidney injury (AKI). In the clinical setting, randomized controlled trials have demonstrated that saline leads to hyperchloremic acidosis. However, evidence of low- versus high chloride solutions having effects on clinical outcomes has been limited until recently. Most prior evidence has been derived from before and after and observational studies. These studies suggest that low-chloride solutions reduce the risk of AKI, use of renal replacement therapy, coagulopathy, need of blood transfusion and mortality. Conversely, individual patient level RCTs have failed to show benefit of the low-chloride solutions. However, two fundamental limitations exist with these RCTs. First, outcomes such as mortality and use of RRT were uncommon (<10% and <5% respectively) and thus lack of power is an alternative explanation for the negative results observed. Second, in these trials expected effect sizes may be rather small due to low total volume of study solution infused. For example, in the largest trial to date, median volume of study fluid was only 2 L over the entire ICU stay. Such volumes are unlikely to produce changes in plasma sodium, chloride concentration or acid-base balance and are thus unlikely to have any effect on hard clinical outcomes. 

In 2018 two very large (collectively enrolling nearly 30,000 patients) pragmatic trials were reported from a single US medical center. These trials utilized a cluster-randomized design and included patients going to the ICU (in one trial) or to the general medical/surgical floor (in the other). The trials were remarkable, in part, for the consistency of the results. Both studies showed a 1% absolute risk reduction in occurrence of a composite endpoint including death, dialysis and persistent renal dysfunction. Importantly, the trials detected harm even with an exposure as low as 1L of fluid and found the largest effect to be in patients with sepsis. Two large RCTs are currently underway in Brazil and in Australia and New Zealand. These trials will hopefully shed additional light on this issue. However, 0.9% saline has a very limited role in the management of critically ill patients, especially those with sepsis, as should no longer be seen as the default solution for fluid therapy. Instead, intravenous fluid therapy should be individualized and patients should receive the type of fluid, route of administration, volume and rate that their clinical status dictate. 

References

1. Myburgh, J.A.; Mythen, M.G. Resuscitation fluids. *N. Engl. J. Med.*
**2013**
*369*, 2462–2463.
2. Healey, M.A.; Davis, R.E.; Liu, F.C.; Loomis, W.H.; Hoyt, D.B. Lactated ringer’s is superior to normal saline in a model of massive hemorrhage and resuscitation. *J. Trauma*
**1998**, *45*, 894–899.
3. Zhou, F.; Peng, Z.-Y.; Bishop, J. V.; Cove, M. E.; Singbartl, K.; Kellum, J. A. Effects of Fluid Resuscitation With 0.9% Saline Versus a Balanced Electrolyte Solution on Acute Kidney Injury in a Rat Model of Sepsis. *Crit. Care Med.*
**2013**, *42*, e270–e278.
4. Watters, J.M.; Brundage, S.I.; Todd, S.R.; Zautke, N.A.; Stefater, J.A.; Lam, J.C.; et al. Resuscitation with lactated inger’s does not increase inflammatory response in a Swine model of uncontrolled hemorrhagic shock. *Shock*
**2004**, *22*, 283–287.
5. Yunos, N.M.; Bellomo, R.; Hegarty, C.; Story, D.; Ho, L.; Bailey, M. Association between a chloride-liberal vs chloride-restrictive intravenous fluid administration strategy and kidney injury in critically ill adults. *JAMA*
**2012**, *308*, 1566–1572.
6. Shaw, A.D.; Schermer, C.R.; Lobo, D.N.; Munson, S.H.; Khangulov, V.; Hayashida, D.K;, et al. Impact of intravenous fluid composition on outcomes in patients with systemic inflammatory response syndrome. *Crit. Care*
**2015**, *19*, 334.
7. Kellum, J. A.; Shaw, A. D. Assessing Toxicity of Intravenous Crystalloids in Critically Ill Patients. *JAMA*
**2015**, *314*, 1695–1697.
8. Sen, A.; Keener, C. M.; Sileanu, F. E.; Foldes, E.; Clermont, G.; Murugan, R.; Kellum, J. A. Chloride Content of Fluids Used for Large-Volume Resuscitation Is Associated With Reduced Survival. *Crit. Care Med.*
**2017**, *45*, e146–e153.
9. Semler, M. W.; Self, W. H.; Wanderer, J. P.; Ehrenfeld, J. M.; Wang, L.; Byrne, D. W.; et al. Balanced Crystalloids versus Saline in Critically Ill Adults. *N. Engl. J. Med.*
**2018**, *378*, 829–839.
10. Self, W. H.; Semler, M. W.; Wanderer, J. P.; Wang, L.; Byrne, D. W.; Collins, S. P., et al. Balanced Crystalloids versus Saline in Noncritically Ill Adults. *N. Engl. J. Med.*
**2018**, *378*, 819–828.
11. Kellum, J. A. Abnormal saline and the history of intravenous fluids. *Nat. Rev. Nephrol.*
**2018**, *14*, 358–360.

#### **2.2.3. Fluid responsiveness: the limitations** 

TEBOULJean-LouisMDService de réanimation médicale, Hôpital de Bicêtre, Hôpitaux universitaires Paris-Sud, INSERM UMR S_999, Univ Paris-Sud, Le Kremlin-Bicêtre, FranceEmail: jean-louis.teboul@aphp.fr

**Introduction**

Prediction of fluid responsiveness plays a major role in guiding resuscitation of critically ill patients. Fluid responsiveness is defined as the ability of the heart to increase its stroke volume in response to an increase in preload induced by fluid administration. This implies that both ventricles work on the ascending part of the Frank-Starling relationship. Fluid responsiveness is present in about 50% of critically ill patients [1]. Since fluid loading might cause harm to critically ill patients in particular to fluid non-responders, it is important to assess fluid responsiveness before infusing fluid. A variety of dynamic tests, which challenge the Frank-Starling relationship without the need for any fluid infusion, have been developed to predict fluid responsiveness. Among them the respiratory variation of hemodynamic signals such as stroke volume variation (SVV) or pulse pressure variation (PPV) have gained a lot of popularity over the past years [2]. PPV was demonstrated to be reliable to predict fluid responsiveness in patients ventilated with a tidal volume of at least 8 mL/kg [3]. 

**Limitations of pulse pressure variation and stroke volume variation**

Several conditions—detailed below—limit the interpretation of PPV (and SVV) in critically ill patients [4]. 

*Patients with spontaneous breathing activity (even during mechanical ventilation)*. In such cases, PPV cannot predict fluid responsiveness as the respiratory changes in intrathoracic pressure are irregular, in rate or in amplitude. 

*Patients with cardiac arrhythmias*. The variability of stroke volume (and thus of pulse pressure) is obviously due to the irregular heart rhythm and not to ventilation. False positive PPV values are thus expected.

*Patients with acute respiratory distress syndrome (ARDS)*. Under low tidal volume ventilation, respiratory changes in intrathoracic pressure might be not large enough to produce significant changes in preload. Accordingly, De Backer et al. showed that the prediction of fluid responsiveness using PPV was weaker when the tidal volume was <8 mL/kg than when the tidal volume was ≥8 mL/kg [5]. Nevertheless, during low tidal volume ventilation, a high value of PPV (e.g., >12%) still suggests fluid responsiveness whereas a low PPV cannot exclude the presence of fluid responsiveness [2]. To improve the interpretation of low PPV, it has been recommended to quantify the change in PPV in response to a transient (<60 s) increase in tidal volume from 6 to 8 mL/kg (tidal volume challenge) [6]. Myatra et al. showed that an increase in the absolute value of PPV ≥3.5% during a tidal volume, predicted fluid responsiveness with excellent accuracy [6]. Further confirmation is obviously necessary. Others proposed to overcome the limitation of using PPV in the case of low tidal volume ventilation by dividing PPV by the respiratory changes in esophageal pressure [7]. The disadvantage of this test is the need of an esophageal probe. 

Low lung compliance by reducing the transmission of airway pressure to the intrathoracic structures, should result in false negative PPV values [2]. Accordingly, a clinical study showed that when the compliance of the respiratory system (Crs) was >30 mL/cmH_2_O, PPV predicted fluid responsiveness accurately, whereas when Crs was ≤30 mL/cmH_2_O [8]. 

In cases of high respiratory rate a low PPV can be observed even in cases of fluid responsiveness. A study showed that PPV cannot be accurately interpreted when the heart rate/respiratory rate ratio is <3.6 [9]. 

*Patients with intra-abdominal hypertension (IAH).* In such patients, the respiratory variations of stroke volume are not exclusively related to volemia and threshold values separating responders and non-responders should be higher than under normal intra-abdominal pressure [10]. For instance, a PPV value of 15% can be associated with fluid unresponsiveness.

*Patients with right heart failure.* High PPV values (>12%) despite fluid unresponsiveness were reported in cases of right ventricular dysfunction [11]. A predominant effect of mechanical insufflation on the right heart afterload would result in a decrease in right ventricular stroke volume during insufflation due to right ventricular afterload-dependence rather than to right ventricular preload-dependence [2]. However, in the above-mentioned study [11], the tidal volume was >8 mL/kg, and attenuation of the degree of RV afterload-dependence during low tidal volume ventilation cannot be excluded. 

**Limitations of other dynamic tests**

Other tests can be used in the (frequent) presence of limitations of PPV (and SVV) [12]. In mechanically ventilated patients, tests such as end-expiratory occlusion alone or in combination with end-inspiratory occlusion can be used as alternatives. The passive leg raising (PLR) test is an excellent alternative to the heart-lung interaction tests and can be used in patients receiving mechanical ventilation as well as in non-intubated patients [12]. Its appropriate use requires to respect strict rules [13]. Among them are the necessity to use a real-time cardiac output measurement and the necessity to start from a semi-recumbent position [13]. The main contraindications of PLR are intracranial hypertension and surgical conditions in the operating room. Finally, it seems that IAH is a limitation of the appropriate interpretation of PLR as test to predict fluid responsiveness [14].

**Conclusions**

There are many limitations to use PPV and SVV in critically ill patients. Note that in the operating room setting, these limitations are far more frequent. However, there are recent advances aimed at overcoming some of the limitations such as the tidal volume challenge. In critically ill patients (except in those with IAH), PLR can be a good alternative to predict fluid responsiveness in situations where PPV cannot be used. 

It is important to keep in mind that the presence of fluid responsiveness does not mean that fluid is necessary. The decision to infuse fluid should also be based on the presence of signs of shock and of low risks of fluid overload.

References

1. Michard, F.; Teboul, J.L. Predicting fluid responsiveness in ICU patients: a critical analysis of the evidence. Chest **2002**, *121*, 2000–2008.
2. Teboul, J.L.; Monnet, X.; Chemla, D.; et al. Arterial Pulse Pressure Variation with Mechanical Ventilation. *Am. J. Respir. Crit. Care Med.*
**2019**, *199*, 22–31.
3. Yang, X.; Du, B. Does pulse pressure variation predict fluid responsiveness in critically ill patients? A systematic review and meta-analysis. *Crit. Care*
**2014**, *18*, 650.
4. Michard, F.; Chemla, D.; Teboul, J.L. Applicability of pulse pressure variation: how many shades of grey?. *Crit. Care*
**2015**, *19*, 144.
5. De Backer, D.; Heenen, S.; Piagnerelli, M.; et al. Pulse pressure variations to predict fluid responsiveness: influence of tidal volume. *Intensive Care Med*. **2005**, *31*, 517–523.
6. Monnet, X.; Bleibtreu, A.; Ferré, A.; et al. Passive leg raising and end-expiratory occlusion tests perform better than pulse pressure variation in patients with low respiratory system compliance. *Crit. Care Med*. **2012**, *40*, 152–157.
7. Myatra, S.N.; Prabu, S.R.; Divatia, J.V.; et al. The changes in pulse pressure variation or stroke volume variation after a "tidal volume challenge" reliably predict fluid responsiveness during low tidal volume ventilation. *Crit. Care Med*. **2017**, *45*, 415–421.
8. Liu, Y.; Wei, L.Q.; Li, G.Q.; et al. Pulse Pressure Variation Adjusted by Respiratory Changes in Pleural Pressure, Rather Than by Tidal Volume, Reliably Predicts Fluid Responsiveness in Patients With Acute Respiratory Distress Syndrome. *Crit. Care Med*. **2016**, *44*, 342–351.
9. De Backer, D.; Taccone, F.S.; Holsten, R.; et al. Influence of respiratory rate on stroke volume variation in mechanically ventilated patients. *Anesthesiology*
**2009**, *110*, 1092–1097.
10. Jacques, D.; Bendjelid, K.; Duperret, S.; et al. Pulse pressure variation and stroke volume variation during increased intra-abdominal pressure: an experimental study. *Crit. Care*
**2011**, 15 doi:10.1186/cc9980.
11. Mahjoub, Y.; Pila, C.; Friggeri, A.; et al. Assessing fluid responsiveness in critically ill patients: False-positive pulse pressure variation is detected by Doppler echocardiographic evaluation of the right ventricle. *Crit. Care Med*. **2009**, *37*, 2570–2575.
12. Monnet, X.; Marik, P.E.; Teboul, J.L. Prediction of fluid responsiveness: an update. *Ann. Intensive Care*
**2016**, *6*, 111.
13. Monnet, X; Teboul, J.L. Passive leg raising: five rules, not a drop of fluid! *Crit. Care*. **2015**, *19*, 18.
14. Mahjoub, Y.; Touzeau, J.; Airapetian, N.; et al. The passive leg-raising maneuver cannot accurately predict fluid responsiveness in patients with intra-abdominal hypertension. *Crit. Care Med*. **2010**, *38*, 1824–1829.

### 2.3. SESSION III. KIDNEY IN SEPSIS

#### **2.3.1. Pathophysiology of Septic AKI** 

KellumJohn A.MDUniversity of Pittsburgh, Pittsburgh, Pennsylvania, USA

Acute kidney injury (AKI) and sepsis carry global consensus definitions. The simultaneous presence of both defines sepsis-associated AKI (S-AKI). S-AKI is the most common AKI syndrome in the ICU and accounts for approximately half of all AKI in critically ill patients. The pathophysiology of S-AKI remains poorly understood, but animal models and lack of major histological changes suggest that, at least initially, septic AKI may be a largely functional phenomenon with combined microvascular shunting and tubular cell stress. The diagnosis remains based on clinical assessment and measurement of urinary output and serum creatinine. However, multiple biomarkers and especially cell cycle arrest biomarkers are rapidly gaining acceptance. Prevention of septic AKI remains based on the treatment of sepsis and on early resuscitation. Such resuscitation relies on the judicious use of both fluids and vasoactive drugs. In particular, starch-containing and chloride-rich fluids are nephrotoxic to patients with high susceptibility (e.g., patients with sepsis). Vasoactive drugs have variable effects on renal function in septic AKI. At this time, norepinephrine is the dominant agent but vasopressin may also be used. 

Patients with severe AKI receiving RRT appear fairly homogeneous (at the clinical and molecular level). However, this may belie important phenotypic differences that are already present early on. In sepsis, patients who recover from AKI appear to have good outcomes (at least over 1–3 yrs). Certain molecular signatures are more prominent in sepsis (e.g., early TIMP-2 activation, early ATP depletion, late feroptotic cell death, and high BMPR1a without concomitant increased BMP7) and these signatures may also be indicative of non-recovery. Both TIMP-2 and IGFBP7 are modulated by P53; but downstream effects of both molecules vary. Understanding transition from early dysfunction to late irreversible injury (vs. recovery) will be key to developing treatments.

References

1. Gomez, H.; Ince, C.; De Backer, D.; et al. A unified theory of sepsis-induced acute kidney injury: inflammation, microcirculatory dysfunction, bioenergetics, and the tubular cell adaptation to injury. *Shock*
**2014**, *41*, 3–11.
2. Kellum, J.A.; Prowle, J.R. Paradigms of acute kidney injury in the intensive care setting. *Nat. Rev. Nephrol.*
**2018**, *14*, 217–230.
3. Kellum, J.A.; Chawla, L.S.; Keener, C.; et al. The Effects of Alternative Resuscitation Strategies on Acute Kidney Injury in Patients with Septic Shock. *Am. J. Respir. Crit. Care Med.*
**2016**, *193*, 281–287.
4. Peng, Z-Y.; Zhou, F.; Kellum, J.A. Cross-species validation of cell cycle arrest markers for acute kidney injury in the rat during sepsis. *Intensive Care Med.*
**2016**, *4*, 12.
5. Agarwal, A.; Dong, Z.; Harris, R.; et al. Cellular and Molecular Mechanisms of AKI. *J. Am. Soc. Nephrol.*
**2016**, *27*, 1288–1299.
6. Kellum, J.A.; Chawla, L.S. Cell-cycle arrest and acute kidney injury: the light and the dark sides. *Nephrol. Dial. Transpl.*
**2016**, *31*, 16–22.
7. Wenzel, S.E. et al. PEBP1 Wardens Ferroptosis by Enabling Lipoxygenase Generation of Lipid Death Signals. *Cell*
**2017**, *171*, 628–641.

#### **2.3.2. Kidney in Sepsis: New Biomarkers to Predict Acute Kidney Injury** 

HonorePatrick M.MD, PhDKugenerLucMDRedantSebastienMDAttouRachidMDGalleraniAndreaMDDeBelsDavidMDICU Dept, Centre Hospitalier Universitaire Brugmann, Brussels; Patrick.Honore@CHU-Brugmann.be (P.M.H.); Luc.Kugener@CHU-Brugmann.be (L.K.); Sebastien.Redant@CHU-Brugmann.be (S.R.); Rachid.Attou@CHU-Brugmann.be (R.A.); Andrea.Gallerani@CHU-Brugmann.be (A.G.); David.DeBels@CHU-Brugmann.be (D.D.)

Biomarkers hold considerable promise in current translational medicine and obviously in intensive care medicine (ICU). We previously discussed the role of biomarkers for early diagnosis of acute kidney injury (AKI) in critically ill patients [1]. Meanwhile, other biomarkers have found the way from bench to bedside. Clinical experience has shown that some of these markers may become powerful tools in the management of critically ill patients. However, the complexity of acute disease conditions exhibits some pitfalls for biomarker use that need further investigation. Renal biomarkers easily outperform “traditional” variables such as creatinine and estimated glomerular filtration rate to predict occurrence and outcome of AKI [1–3]. However, many questions remain. Whether biomarkers can improve the approach to the sick or compromised kidney, in particular regarding timely initiation of continuous renal replacement therapy (CRRT), has insufficiently been clarified. Moreover, biomarkers lack specificity in case of sepsis-induced AKI or in AKI associated with the acute respiratory distress syndrome (ARDS). Also, a potential role of biomarkers to assist in decision processes for admission or discharge in emergency and ICUs has not been established. AKI is a major determinant of morbidity and mortality in critically ill patients. Crucial issues are early detection of the at-risk population and timely start of therapy. In this context, biomarkers may have a decisive preemptive role. AKI is a common condition in hospitalized patients and is associated with worse short-term and long-term outcome. The delay in the diagnosis of AKI has been proven to be associated with morbidity and mortality. One of the challenges clinicians face is the early detection of AKI. Another difficulty is the lack of a consensual definition of AKI. Expert consensus seems to be emerging [4] but recent literature review shows that the definitions used in the published articles remain multiple and heterogeneous [5]. While many urinary and serum proteins have been investigated as potential biomarkers for the early diagnosis or prediction of AKI or to identify patients at high risk, the ideal diagnostic tools of AKI remains an unmet medical need. Patients with predominant cardio-renal syndrome and high serum concentrations of Neutrophil gelatinase-associated lipocalin (NGAL) were found to be at risk of developing AKI, even in the absence of oliguria and increased creatinine levels. NGAL-positive patients also more frequently require renal replacement therapy and have higher mortality rates [2,6]. This new entity, divulged by using a biomarker as “subclinical AKI” [7] suggested that current concepts and definition of AKI might need reassessment [2]. The SAPPHIRE study [8,9] showed that two novel cell cycle arrest biomarkers, tissue inhibitor of metalloproteinase-2 (TIMP-2) and urinary insulin-like growth factor binding protein 7 (IGFBP7), largely outperformed existing markers. Whether these markers can predict the need for or duration of renal replacement therapy is currently under investigation. Studies in ICU patients will help to identify the potential of tissue inhibitor of metalloproteinase-2/insulin-like growth factor binding protein 7 for early detection of postoperative or antimicrobial-related toxic AKI. High serum cystatin-C (Cys-C) levels at admission were found to predict subclinical AKI in patients admitted in the ICU [10].These biomarkers have been subject of very recent investigations. Vitamin-D-binding protein (VDBP) is a low molecular weight protein that is filtered through the glomerulus as a 25-(OH) vitamin D 3 (VDBP) complex. In the normal kidney, VDBP is reabsorbed and catabolized by proximal tubular epithelial cells reducing urinary excretion to trace amounts. Acute tubular injury is expected to result in urinary VDBP loss [11]. VDBP and kidney injury molecule-1 were identified as early and very sensitive markers of contrast-induced nephropathy, [12] whereas interleukin-18 and kidney injury molecule-1 predicted early-stage AKI in burn patients [13].An upcoming marker could be hemoglobin subunit beta (HBβ), a component of hemoglobin [13]. Hemoglobin is the main carrier of oxygen and carbon dioxide in cells of the erythroid lineage and ensures tissue oxygen delivery throughout the body. Blood HBβ levels in septic patients are significantly higher than those in healthy volunteers, and values in septic shock exceed those found in severe sepsis. HBβ levels apparently correlate with the degree of sepsis-induced endothelial cell dysfunction and thus may closely reflect sepsis severity [13]. An area of particular clinical interest is the behavior of biomarkers dedicated to detect a specific pathology (such as NGAL for AKI) in sepsis. Indeed, increased NGAL levels are also observed in many septic patients. Moreover, as up to half of septic shock patients may develop AKI, NGAL measurement loses all specificity for detecting AKI, as it detect sepsis at the same time [14]. Further research is needed to obtain biomarkers with sufficient diagnostic and prognostic specificity to discriminate between an ongoing septic process and an associated AKI [15]. Research to interfere in this fascinating universe of interacting pathways is on the brink of a breakthrough [16]. Hall et al. performed a systematic review associated with meta-analysis to evaluate the potential of AKI in-vitro diagnostic tests to improve the management of patients with critical illness [17,18]. They evaluated sensitivity and specificity, cost-effectiveness evaluation, appraisal of analytical validity, care pathway analysis and value of information analysis. Three tests (TIMP-2 IGFBP7, NGAL and cystatin C were subject to detail review. Their main finding is the heterogeneity in the reporting of those studies and the poor analytical validity. The meta-analysis of the reported results showed a pooled sensitivity of the biomarkers ranging from 0.54 to 0.92 and a pooled specificity ranging from 0.49 to 0.95. In addition, the authors discussed the fact that cost-effectiveness have either no or poorly been explored. The authors call for a homogenisation of practices both in the development of studies and in the analysis and reporting of their results. It also seems necessary to insist on the need to develop impact studies. Indeed, only biomarkers associated with management strategies that have shown that they can improve patient outcomes are of real interest. Finally, it should also be borne in mind that the dosage of these new biomarkers is often costly; medical-economic studies should be conducted to address this issue [17,18]. The odyssey for the ideal biomarkers for predicting AKI will continue.

References

1. Honore, P.M.; Jacobs, R.; Joannes-Boyau, O.; et al. Biomarkers for early diagnosis of AKI in the ICU: ready for prime time use at the bedside? *Ann. Intensive Care*
**2012**, *2*, 24.
2. Haase, M.; Devarajan, P.; Haase-Fielitz, A.; et al. The outcome of neutrophil gelatinase-associated lipocalin-positive subclinical acute kidney injury: A multicenter pooled analysis of prospective studies. *J. Am. Coll. Cardiol*. **2011**, *57*, 1752–1761.
3. Haase, M.; Kellum, J.A.; Ronco, C. Subclinical AKI–an emerging syndrome with important consequences. *Nat Rev. Nephrol*. **2012**, *8*, 735–739.
4. Kellum, J.A.; Lameire, N. Diagnosis, evaluation, and management of acute kidney injury: A KDIGO summary (Part 1). *Crit. Care*
**2013**, *17*, 204.
5. Codorniu, A.; Lemasle, L.; Legrand, M.; et al. Methods used to assess the performance of biomarkers for the diagnosis of acute kidney injury: a systematic review and meta-analysis. *Biomarkers*
**2018**, *23*, 766–72.
6. Ronco, C.; Kellum, J.A.; Haase, M. Subclinical AKI is still AKI. *Crit. Care*
**2012**, *16*, 313.
7. Ronco, C.; Stacul, F.; McCullough, P.A. Subclinical acute kidney injury (AKI) due to iodine-based contrast media. *Eur. Radiol*. **2013**, *23*, 319–323.
8. Kashani, K.; Al-Khafaji, A.; Ardiles, T.; et al. Discovery and validation of cell cycle arrest biomarkers in human acute kidney injury. *Crit. Care*
**2013**, *17*, doi:10.1186/cc12503.
9. Hoste, E.A.; McCullough, P.A.; Kashani, K. et al. Sapphire Investigators Derivation and validation of cutoffs for clinical use of cell cycle arrest biomarkers. *Nephrol. Dial Transplant*. **2014**, *29*, 2054–2061.
10. Gaygısız, Ü.; Aydoğdu, M.; Badoğlu, M.; Boyacı, N.; Güllü, Z.; Gürsel, G. Can admission serum cystatin C level be an early marker subclinical acute kidney injury in critical care patients? *Scand. J. Clin. Lab. Investig.*
**2016**, *76*, 143–150.
11. Chaykovska, L.; Heunisch, F.; von Einem, G.; et al. Urinary vitamin D binding protein and KIM-1 are potent new biomarkers of major adverse renal events in patients undergoing coronary angiography. *PLoS ONE*
**2016**, *11*, e0145723.
12. Ren, H.; Zhou, X.; Dai, D.; et al. Assessment of urinary kidney injury molecule-1 and interleukin-18 in the early post-burn period to predict acute kidney injury for various degrees of burn injury. *BMC Nephrol*. **2015**, *16*, 142.
13. Yoo, H.; Ku, S.K.; Kim, S.W.; Bae, J.S. Early diagnosis of sepsis using serum hemoglobin subunit beta*. Inflammation*
**2015**, *38*, 394–399.
14. Hjortrup, P.B.; Haase, N.; Treschow, F.; Møller, M.H.; Perner, A. Predictive value of NGAL for use of renal replacement therapy in patients with severe sepsis. *Acta Anaesthesiol. Scand*. **2015**, *59*, 25–34.
15. Ronco, C.; Legrand, M.; Goldstein, S.L.; et al. Neutrophil gelatinase-associated lipocalin: ready for routine clinical use? An international perspective. *Blood Purif*. **2014**, 37, 271–285.
16. Honore, P.M.; Nguyen, H.B.; Gong, M.; et al. Sapphire and Topaz Investigators Urinary tissue inhibitor of metalloproteinase-2 and insulin-like growth factor-binding protein 7 for risk stratification of acute kidney injury in patients with sepsis. *Crit. Care Med*. **2016**, *44*, 1851–1860.
17. Hall, P.S.; Mitchell, E.D.; Smith, A.F.; et al. The future for diagnostic tests of acute kidney injury in critical care: evidence synthesis, care pathway analysis and research prioritisation. *Health Technol. Assess.*
**2018**, *22*, 1–274.
18. Gayat, E. Biomarkers of acute kidney injury: Mixed results and huge heterogeneity of reporting. *BMJ Evid. Based Med*. **2019**, *2018*, 111152. doi:10.1136/bmjebm-2018-111152.

#### **2.3.3. Vasopressor in Sepsis States** 

MARTINClaudeMDAnesthesia and Intensive Care Department and Trauma Center, Hôpital Nord, Marseille, France

**Introduction**

Shock is caused by an inadequate supply, or inappropriate use of metabolic substrate (mainly oxygen) resulting in tissue damage and cellular death. Several factors contribute to organ dysfunction in septic patients. Hemodynamic factors such as volume depletion, low cardiac output or inappropriate vasodilation resulting in systemic hypotension may directly produce organ hypoperfusion through a reduction in organ perfusion pressure. Organ autoregulation, the tendency of organ blood flow to remain constant over a range of organ perfusion pressure values, defines the perfusion range over which resistance can compensate for the decrease in pressure. The autoregulatory threshold is the lowest pressure at which autoregulation is maintained and may be defined by the intercept of the autoregulated zone with the slope of the subautoregulatory zone. Below their autoregulatory thresholds, organ blood flows become linearly dependent on perfusion pressure. Therefore, it can be speculated that therapy should be aimed at providing an adequate organ perfusion pressure and that this is higher than commonly targeted or achieved in the treatment of septic patients. While this may require the use of vasopressor catecholamines, with their attendant risk of organ vasoconstriction and reduction in organ blood flow, clinical studies suggest that such reactions rarely occur and that organ function usually improve when tissue perfusion pressure is augmented during sepsis and septic shock.

**The Vasopressors Tools**

*Epinephrine*

In patients unresponsive to volume expansion or other catecholamine infusions, epinephrine can increase mean arterial pressure, primarily by increasing cardiac index and stroke volume with more modest increases in systemic vascular resistance and heart rate. Epinephrine can increase oxygen delivery, but oxygen consumption may be increased as well. Epinephrine administration has been associated with increases in systemic and regional lactate concentrations. The monitoring periods were short, and so it is unclear if these increases are transient. Other adverse effects of epinephrine include increases in heart rate, but electrocardiographic changes indicating ischemia or arrhythmias have not been reported in septic patients. Epinephrine has had minimal effects on pulmonary artery pressures and pulmonary vascular resistance in sepsis.

*Norepinephrine*

Norepinephrine is a potent alpha-adrenergic agonist with less pronounced beta-adrenergic agonist effects. In open labeled trials, norepinephrine has been shown to increase mean arterial pressure in patients who remained hypotensive after fluid resuscitation and dopamine. Due to concerns about potential adverse vasoconstrictive effects on regional vascular beds such as the liver and the kidney, norepinephrine traditionally had either not been used or had been reserved as a last resort in a moribund patient, with predictably poor results. The experience with norepinephrine in septic shock strongly suggests that this drug can successfully increase blood pressure without causing deterioration in organ function. In most studies, septic patients were given fluid to correct hypovolemia before dopamine with or without dobutamine was titrated to achieve the target blood pressure. When dopamine failed, norepinephrine was added to the dopamine regimen. In most studies of septic patients, norepinephrine was used at a mean dose of 0.2 to 1.3 g/kg/min. The initial dose can be as low as 0.01 g/kg/min, and the highest reported norepinephrine dosage was 3.3 g/kg/min. Thus, large doses of the drug may be required in some patients with septic shock, possibly due to alpha-receptor down-regulation in sepsis. Norepinephrine therapy usually causes a clinically significant increase in mean arterial pressure attributable to its vasoconstrictive effects, with little change in heart rate or cardiac output (CO), leading to increased systemic vascular resistance. CO is increased by 10 to 20% and stroke volume index (SVI) by 10 to 15%. Clinical studies have reported either no change or modest increases, in pulmonary capillary wedge pressure. Mean pulmonary arterial pressure is either unchanged or increased slightly. In patients with hypotension and hypovolemia, e.g., during hemorrhagic or hypovolemic shock, the vasoconstrictive effects of norepinephrine can have severe detrimental effects on renal hemodynamics, with increased renal vascular resistance and renal ischemia. Indeed, norepinephrine has been demonstrated to cause ischemia-induced acute renal failure in rats. The situation is different in hyperdynamic septic shock, in which it is believed that urine flow decreases mainly as a results of lowered renal perfusion pressure. Since norepinephrine has a greater effect on efferent arteriolar resistance and increases the filtration fraction, normalization of renal vascular resistance could effectively reestablish urine flow.

In the high output–low resistance state of septic shock patients, norepinephrine can markedly improve mean arterial pressure and glomerular filtration. In several studies norepinephrine (0.5 to 1.5 g/kg/min) significantly increased urine output, creatinine clearance, and osmolar clearance. This supports the hypothesis that renal ischemia observed during hyperdynamic septic shock is not worsened by norepinephrine infusion and even suggests that this drug may optimize effectively renal blood flow and renal vascular resistance.

**Use of Vasopressor Therapy**

When fluid administration fails to restore an adequate arterial pressure and organ perfusion, therapy with vasopressor agents should be initiated. Potential agents that can be selected include dopamine, norepinephrine, epinephrine and phenylephrine. Vasopressor therapy may be required transiently even while cardiac filling pressures are not yet adequate, in order to maintain perfusion in the face of life-threatening hypotension. Although the use of these drugs has the potential to reduce organ blood flow through arterial vasoconstriction, their final effects will depend upon the sum of the direct effects and any increase in organ perfusion pressure. In settings where organ autoregulation is lost, organ flow becomes linearly dependent on pressure and organ perfusion pressure should be preserved if flow is to be optimized. Whether or not a potent vasopressor also has positive inotropic effects is of clinical importance in patients with low cardiac output. From a practical point of view, when a vasopressor infusion is started, doses should be carefully titrated to restore mean arterial pressure, without impairing stroke volume. The precise mean blood pressure level targeted depends upon the premorbid blood pressure but can be as high as 75 mmHg. However, individual responses should be kept at the minimum level required to reestablish urine flow and in some patients this can be achieved with a mean arterial pressure of 60 or 65 mmHg. 

**Conclusions**

Optimization of organ perfusion pressure is an important goal to achieve during the treatment of sepsis and septic shock. A rational argument can be made for augmenting perfusion pressure based on the linear pressure-flow relationship secondary to organ insult during sepsis. In addition, there is a lot of clinical evidence showing the absence of harm with vasopressor infusion and the improvement in organ function when these agents are used to restore adequate blood pressure.

#### **2.3.4. CRRT in Sepsis** 

GutiérrezManuel E. HerreraICU, Complejo Universitario de Malaga. Malaga. Spain

Sepsis is the main cause of AKI in the ICU. Following the interaction between the bacteria and the host, a response is triggered that encompasses the synthesis of pro (TNF, IL-1, IL-8, PAF) and antiinflammatory (IL-10, IL-4, etc…) mediators. If an excessive amount of these molecules is produced the response of the host can be compromised (inmunoparalisys) [1]. Different strategies aiming to block the synthesis of some of these mediators have shown no results or even worst, a worsening of the situation of our patients, but if we could count on a therapy that normalizes the concentration of mediators keeping a normal balance between them, hypothetically we could control de inmunoparalisys improving the defensive capability of the immune system (immunomodulation) [2]. 

In the last decades an important effort has been done by different investigators and the industry aiming to improve the extracorporeal therapy devices at our disposal in order to gain efficacy in this theoretical inmunomodulatory capability by means of a higher clearance of immune mediators. 

A first approach was to employ convection because a high convective dose (high volume hemofiltration) was shown to improve hemodynamics and was followed by a rapid decrement in vasopresor need. As a fact, a reduction in mediator levels has been demonstrated with convection but it is clear that to reach this effect, a high change of fluid must be employed [3] so that, in we decide to treat our septic patient with a convective therapy we must use HVHF. Anyway, even when a clinical improvement is to be expected, a decrement of mortality has not been proven for this treatment and its use can carry serious problems because the high volume of fluid interchanged and the need for an equipment capable of controlling exact balances.

Another approach, less workload demanding is the use of membranes with a wider pore size (high cut-off membranes HCO) [4] that let the mediators to be effectively cleared using a lower amount of fluid. Its main drawback is the high loss of albumin that accompanies this therapy, but this side effect cam be minimized if we employ diffusion as the depurative modality. Results with this device are close to those described for HVHF and once more there is no evidence about improvement in outcome. 

If continue widening the pore size, the amount of molecules cleared increases and with it the possibility to eliminate more mediators and so the use of PMF membranes has been tested also for this indication. Once more the clinical effect is evident but not the improvement in outcome [5] and PMF presents supposes also a serious risk, because the non-selective elimination of important plasmatic proteins and the need for high FFP volumes.

Finally, adsorption has also been proposed as a mean for the elimination of inflammatory mediators and as a fact, seems the most promising among these techniques and the one where more advances are being presented, in three different directions: first we are witnessing an improvement in the adsorptive capability of old membranes, as AN-69 (Oxiris^®^) or PMMA; then some membranes have been developed aiming for a highly selective adsorption capability as is LPS affinity for LPS Adsorber^®^ or Toraymixin^®^, and finally and looking for exactly the opposite, development of membranes with very high non-selective capability of adsorption as for Cytosorb^®^. At the moment, experience for all these systems is scarce and almost observational small series, counting only with RCT for Toraymin^®^. Its usefulness has not been definitely proven and the most recent RCT published does not show effect on survival also for this device [6]. A meta-analysis published in 2013 did show a positive effect on survival for extracorporeal devices (in the analysis were included all the modalities already mentioned) and suggesting a stronger effect for the adsorptive devices [7] but this analysis did not include the last one published mentioned earlier. 

Some authors have also tried for a combination of different modalities but this strategy has not been proven to be of utility. CPFA, a hybrid modality combining plasmafiltration plus adsorption, has not shown the expected results. 

So, at this point we can not rest in any conclusive evidence for or against the use of extracorporeal therapies for the management of sepsis in terms of survival, even when, as already mentioned, all the modalities tried have shown a positive effect on hemodynamics and vasopressor usage. This similarity in the results is not surprising because we must keep in mind that whatever technique we use, our aim is in fact the same, an elimination of inmunomediators, and that any differences in effect will really reflect a difference in clearance capability for these techniques (except for the highly selective adsorption provided by Toraymixyn^®^).

One last aspect that merits attention when approaching the septic patient with an extracorporeal therapy is the non-selective elimination that these treatments provide and the fact that among the many molecules that are cleared are included most o the antibiotics, the main treatment of sepsis. If we consider that our aim is to clear as much mediators as possible, it is easy to understand that the risk of underdosing the antibiotics is really high. If a careful dosage of these drugs is not prescribed we’ll be in the end endangering the recovery of our patients and can even worsen their prognosis.

As a resume and following the recommendations of the SSC, at this point we cannot make a sound recommendation for or against the use of extracorporeal therapies for sepsis [8].

References

1. Hotchkiss RS, Coopersmith CM, McDunn JE, Ferguson TA. The sepsis seesaw: Tilting toward immunosuppression. *Nat. Med*. **2009**, *15*, 496–497.
2. Yekebas EF, Eisenberger CF, Ohnesorge H, Saalmuller A, Elsner HA, Engelhardt M, et al. Attenuation of sepsis-related immunoparalysis by continuous veno-venous hemofiltration in experimental porcine pancreatitis. *Crit. Care Med*. **2001**, *29*, 1423–1430.
3. Ghani RA, Zainudin S, Ctkong N, Rahman AF, Wafa SR, Mohamad M, et al. Serum il-6 and il-1-ra with sequential organ failure assessment scores in septic patients receiving high-volume haemofiltration and continuous venovenous haemofiltration. *Nephrology (Carlton)*, **2006**; *11*, 386–393.
4. Haase M, Bellomo R, Baldwin I, Haase-Fielitz A, Fealy N, Davenport P, et al. Hemodialysis membrane with a high-molecular-weight cutoff and cytokine levels in sepsis complicated by acute renal failure: A phase 1 randomized trial. *Am. J. Kidney Dis*. **2007**, *50*, 296–304.
5. Knaup H, Stahl K, Schmidt BMW, Idowu TO, Busch M, Wiesner O, et al. Early therapeutic plasma exchange in septic shock: A prospective open-label nonrandomized pilot study focusing on safety, hemodynamics, vascular barrier function, and biologic markers. *Crit. Care*
**2018**, *22*, 285.
6. Payen DM, Guilhot J, Launey Y, Lukaszewicz AC, Kaaki M, Veber B, et al. Early use of polymyxin b hemoperfusion in patients with septic shock due to peritonitis: A multicenter randomized control trial. *Intensive Care Med*. **2015**, *41*, 975–984.
7. Zhou F, Peng Z, Murugan R, Kellum JA. Blood purification and mortality in sepsis: A meta-analysis of randomized trials. *Crit. Care Med*, **2013**, *41*, 2209–2220.
8. Rhodes A, Evans LE, Alhazzani W, Levy MM, Antonelli M, Ferrer R, et al. Surviving sepsis campaign: International guidelines for management of sepsis and septic shock: 2016. *Intensive Care Med*. **2017**, *43*, 304–377.

### 2.4. SESSION IV. PERSONALIZE SEPSIS CARE

#### **2.4.1. Influence of Time Period Symptoms in Biomarkers in CAP** 

TorresAntoniServei de Pneumologia. Hospital Clinic. University of Barcelona

Assessment of the inflammatory response can help the decision-making process when diagnosing community-acquired pneumonia (CAP) [1–6], but there is a lack of information about the influence of time since onset of symptoms.

We studied the impact of the number of days since onset of symptoms on inflammatory cytokines and biomarker concentrations at CAP diagnosis in hospitalized patients [1].

We performed a secondary analysis in two prospective cohorts including 541 patients in the derivation cohort and 422 in the validation cohort. The time since onset of symptoms was self- reported, and patients were classified as early presenters (<3 days) and nonearly presenters. Biomarkers (C-reactive protein [CRP] and procalcitonin [PCT] in both cohorts) and cytokines in the derivation cohort (IL-1,6,8,10, and tumor necrosis factor-α) were measured within 24 h of hospital admission.

In early presenters, CRP was significantly lower, whereas PCT, IL-6, and IL-8 were higher. Nonearly presenters showed significantly lower PCT, IL-6, and IL-8 levels. In the validation cohort, CRP and PCT exhibited identical patterns: CRP levels were 36.4% greater in patients with 3 or more days since onset of symptoms than in those with less than 3 days since symptom onset in the derivation cohort and 38.2% in the validation cohort. PCT levels were 40% lower in patients with 3 or more days since onset of symptoms in the derivation cohort and 56% in the validation cohort.

Time since symptom onset modifies the systemic inflammatory profile at CAP diagnosis. This information has relevant clinical implications for management, and it should be taken into account in the design of future clinical trials [1].

References

1. Méndez, R.; Menéndez, R.; Cillóniz, C.; Amara-Elori, I.; Amaro, R.; González, P.; Posadas, T.; Gimeno, A.; España, P.P.; Almirall, J.; Torres, A. *Am. J. Respir. Crit. Care Med*. **2018**, *198*, 370–378.
2. Quan, T.P.; Fawcett, N.J.; Wrightson, J.M.; Finney, J.; Wyllie, D.; Jeffery, K.; et al. Infections in Oxfordshire Research Database (IORD). Increasing burden of community-acquired pneumonia leading to hospitalisation, 1998–2014. *Thorax*
**2016**, *71*, 535–542.
3. Ito, A.; Ishida, T.; Tachibana, H.; Ito, Y.; Takaiwa, T. Serial procalcitonin levels for predicting prognosis in community-acquired pneumonia. *Respirology*
**2016**, *21*, 1459–1464.
4. Schuetz, P.; Briel, M.; Christ-Crain, M., Stolz, D.; Bouadma, L.; Wolff, M.; et al. Procalcitonin to guide initiation and duration of antibiotic treatment in acute respiratory infections: an individual patient data meta-analysis. *Clin. Infect. Dis*. **2012**, *55*, 651–662.
5. Torres, A.; Sibila, O.; Ferrer, M.; Polverino, E. Menéndez, R.; Mensa, J.; et al. Effect of corticosteroids on treatment failure among hospitalized patients with severe community-acquired pneumonia and high inflammatory response: a randomized clinical trial. *JAMA*
**2015**, *313*, 677–686.
6. Martínez, R.; Menéndez, R.; Reyes, S.; Polverino, E.; Cillóniz, C.; Martínez, A.; et al. Factors associated with inflammatory cytokine patterns in community-acquired pneumonia. *Eur. Respir. J*. **2011**, *37*, 393–399.

#### **2.4.2. Phenotyping of Patients with Sepsis** 

GerlachHerwigMD, PhD, MBA, MSc, MAEDepartment of Anesthesia, Critical Care Medicine, and Pain Management Vivantes—Klinikum Neukoelln; Rudower Strasse 48 D-12313 Berlin, Germany; Tel: +49-30-13014-2361; FAX: +49-30-13014-2497, Berlin, Germany; herwig.gerlach@vivantes.de

The last two to three years provided several big steps regarding understanding and management of sepsis. The increasing insight into pathomechanisms of post-infectious defense not only led to some new models of host response, but also opened a new of personalized sepsis therapy by using the individual pattern of information on different biological levels. These include genomics, proteomics, metabolomics, etc., often put together as the “omic” approach to personalized medicine, e.g., in sepsis [1]. Several projects concentrated on the fingerprint of patients by describing their genetic information on the DNA and/or RNA level, thus described as genotyping, often with the goal to assess their individual risk [2].

In contrast, downstream information on the levels of proteins, other biomarkers, or metabolic changes, but also clinical symptoms or even demographic data such as age, weight, or height of patients, are often put together as phenotyping, the natural pendant to the genotype. To identify reproducible pattern of septic patients, modern IT technologies are tested more and more. In a recent landmark paper, a group of international investigators developed a computational model using reinforcement learning, which is able to dynamically suggest optimal treatments for adult patients with sepsis in the intensive care unit (ICU); reinforcement learning is a category of Artificial Intelligence (AI) tools in which a virtual agent learns from trial-and-error processes to create an optimized set of rules [3]. Forty-eight variables were extracted from the patients’ files, including demographics, the premorbid status, vital signs, laboratory values, applied fluids and vasopressors. It was demonstrated that the value of the treatment selected by the AI tool was reliably higher than that of ICU physicians. Interestingly, mortality was lowest in patients for whom clinicians’ actual treatment matched the AI decisions. Similar sophisticated methods were applied by another group of researchers using a large database, although they did this with data from patients immediately after cardiac arrest and hospital transfer, i.e., without any further information on the patients’ history [4]. The number of 39,566 patients from 186 ICUs were analyzed. The investigators found that Machine Learning approaches (gradient boosting machine, support vector classifier, random forest, artificial neural network, and an ensemble) significantly enhance predictive discrimination for mortality following cardiac arrest compared to existing illness severity scores and classical logistic regression, without the use of pre-hospital data.

Patients with lower respiratory tract infections (LRTI) are a challenge for goal-directed antibiotic treatment: in the absence of a definitive microbiologic diagnosis, clinicians may presume symptoms are due to a noninfectious inflammatory condition and initiate empiric corticosteroids, which can exacerbate an occult infection. Furthermore, even with negative microbiologic testing, providers often continue empiric antibiotics due to concerns of falsely negative results, a practice that drives emergence of antibiotic resistance and increases risk of second-hit infections. Recent studies integrated data from the patients’ host response marker with information of the individual microbiome to forecast the risk in critically ill patients for LRTI [5]. the research group performed metagenomic next-generation sequencing (mNGS) on tracheal aspirates from 92 adults with acute respiratory failure and simultaneously assessed pathogens, the airway microbiome, and the host transcriptome. This study suggests that a single streamlined protocol offering an integrated genomic portrait of pathogen, microbiome, and host transcriptome may hold promise as a tool for LRTI diagnosis. This kind of detailed patient phenotyping, performed in a population reflective of the true heterogeneity of ICU patients, including severely immunocompromised subjects and patients receiving broad-spectrum antibiotics, could be a view-into-the future. Studies in a larger cohort may further validate these findings, strengthen the utility of these models, and assess the impact on clinical outcomes.

Another exiting approach to better describe the pattern of infections in individual septic patients was recently introduced by investigators who presented a way to distinguish the source of various bloodstream infections (BSI), which may facilitate more accurate tracking and prevention of hospital-acquired infections [6]. The researchers developed and applied a streamlined bioinformatic tool to match bloodstream pathogens precisely to a candidate source. Then they leveraged this approach to interrogate the gut microbiota as a potential reservoir of bloodstream pathogens in a cohort of hematopoietic cell transplantation recipients. In conclusion, more precisely identifying the origins of BSIs may influence how hospitals and health care providers can most effectively work to prevent infections. 

The previous two examples demonstrated the impact of modern techniques to better diagnose the patients’ diseases. Of course, the question remains if these approaches may have any influence on the therapeutic consequences—a rather new field of medicine, which is described as theragnostics. The following study is currently submitted by our own group and showed how phenotyping by a combination of specific biomarkers influences the effect of low dose hydrocortisone (HC) in septic shock patients [7]. Although several trials have consistently reported faster shock resolution, the utility of HC in patients with septic shock remains controversial; whereas two French studies reported outcome benefit, two international studies found no survival effect from HC. Corticosteroids are traditionally considered to induce immune suppression via the glucocorticoid receptor (GR) and its repressive effect on pro-inflammatory transcription factors. Thus, patients in an overall state of immunosuppression, which is presumed for many cases of septic shock, may be potentially compromised by administration of an immunosuppressive drug. These diverging effects of steroids support the need for biomarkers to guide their application. Therefore, we applied machine learning to physiological and laboratory data from patients enrolled into former randomized studies to determine a theragnostic marker for hydrocortisone treatment. It was found that the ratio of serum interferon-γ (IFNγ) to interleukin-10 (IL-10) was able to identify specific sub-cohorts with increased and decreased survival upon treatment. Applying the rule to two further, smaller datasets showed the same tendency. Strengths of our study were that we used well selected cohorts of patients with septic shock, that our marker showed very similar results across all studies, and that this approach showed the potential clinical relevance as a future option to better phenotype septic shock patients before applying hydrocortisone.

References

1. Itenov, T.S.; Murray, D.D.; Jensen, J.U.S. Sepsis: Personalized Medicine Utilizing ‘Omic’ Technologies—A Paradigm Shift? *Healthcare*
**2018**, *6*, 111–119.
2. Lu, H.X.; Sun, J.H.; Wen, D.L.; et al. LBP rs2232618 polymorphism contributes to risk of sepsis after trauma. *World J. Emerg. Surg*. **2018**; *13*, 52–29.
3. Komorowski, M.; Celi, L.A.; Badawi, O.: et al. The Artificial Intelligence Clinician learns optimal treatment strategies for sepsis in intensive care. *Nat. Med*. **2018**, *24*, 1716–1720.
4. Nanayakkara, S.; Fogarty, S.; Tremeer, M.; et al. Characterising risk of in-hospital mortality following cardiac arrest using machine learning: A retrospective international registry study. *PLoS Med.*
**2018**; *15*, e1002709.
5. Langelier, C.; Kalantar, K.L.; Moazed, F.; et al. Integrating host response and unbiased microbe detection for lower respiratory tract infection diagnosis in critically ill adults. *PNAS*
**2018**, *115*, E12353–E12362.
6. Tamburini, F.B.; Andermann, T.M.; Tkachenko, E.; et al. Precision identification of diverse bloodstream pathogens in the gut microbiome. *Nat. Med*. **2018**, *24*, 1809–1814.
7. König, R.; Kolte, A.; Ahlers, O.; et al. Use of IFNγ/IL10 ratio for stratification of hydrocortisone therapy in patients with septic shock. *EBioMedicine*
**2019**, submitted.

### 2.5. SESSION V. NEW TREATMENTS IN SEPSIS

#### **2.5.1. Adjunctive Therapy in Sepsis—Old Friends vs. New Trials** 

RussellJames A.Professor of Medicine, University of British Columbia, Principal Investigator, Centre for Heart Lung Innovation, Vancouver, BC, Canada

The main adjunctive therapy in common use in septic shock and that has Surviving Sepsis Guideline (SSG) [1] recommendations is corticosteroids. There are several exciting novel adjunctive agents that could accelerate clearance and neutralization of lipopolysaccharide (LPS) and lipotechoic acid (LTA). There are additional immune enhancing strategies for patients in later sepsis who have signs of depressed immunity and novel pleiotropic anticoagulant type products (recombinant human thrombomodulin) that could also prove efficacious.

**Corticosteroids**

The SSG suggest “against using IV hydrocortisone to treat septic shock patients if adequate fluid resuscitation and vasopressor therapy are able to restore hemodynamic stability. If this is not achievable, we suggest IV hydrocortisone at a dose of 200 mg per day (weak recommendation, low quality of evidence)” [1]. Note the low quality evidence and the weak recommendation.

Since these SSG were published there are now two large pivotal RCTs of corticosteroids in septic shock with quite contrasting results and conclusions [2,3]. Annane and colleagues [2] found that hydrocortisone plus fludrocortisone decreased mortality compared to placebo (39% vs. 45,3% respectively, *p* = 0.02), while Venkatesh and colleagues [3] reported that hydrocortisone did not decrease mortality compared to placebo (27.9% vs. 28.8% respectively). One can see obvious differences between the RCTs: (1) Annane and colleagues used a combination of hydrocortisone plus fludrocortisone because of the altered steroid physiology of septic shock and because of the positive results of their prior steroid RCT [4]; (2) the pooled mortality of Annane’s RCT was higher than Venkatesh suggesting that Annane’s patients were sicker. The Surviving Sepsis Guidelines [1] and recent corticosteroid guidelines [5] do not include these RCTs and will need to be updated. We suggest that the combination of hydrocortisone and fludrocortisone be considered for patients in septic shock who do not respond adequately to norepinephrine infusion.

**Clearance and neutralization of lipopolysaccharide (LPS) and lipotechoic acid (LTA)**

Decreased Proprotein Convertase Subtilisin/Kexin type-9 (PCSK9) activity increases LDL receptor density and clearance. Pathogen lipid (PL) clearance is related to endogenous lipid clearance; accordingly, PCSK9 regulates clearance of PLs such as LPS and LTA. Pharmacologic inhibition of PCSK9 improved survival and inflammation in murine polymicrobial peritonitis [6,8]. In several human septic shock cohorts, PCSK9 Loss-Of-Function (LOF) genetic variants were associated with decreased mortality compared to PCSK9 wild-type patients. PCSK9 LOF also decreased inflammatory cytokines in septic shock patients and after LPS administration to healthy volunteers. The PCSK9 effect was mitigated in LDL receptor knock-out mice and in humans homozygous for a LDL receptor variant resistant to PCSK9. Thus, decreased PCSK9 function is associated with increased PL clearance via the LDL receptor, a decreased inflammatory response, and decreased mortality [8,9]. Patients who have PCSK9 loss-of-function variants also have decreased readmission rate for sepsis after an episode of sepsis suggesting long term benefits of impaired PCSK9 function [10]. 

Plasma PCSK9 levels are greatly increased in sepsis [11]. Plasma PCSK9 greater than 250 ng/mL decreases hepatocyte LPS clearance and such levels are associated with cardiovascular and respiratory failure [11]. 

Plasma LDL levels decrease in sepsis and are associated with increased mortality [12] but is this merely association or do LDL levels causally contribute to increased mortality? We reasoned that an “instrumental variable” approach may help address this issue [13,14]. Recently Ference et al. [13] used a genetic instrumental variable strategy to show that LDL levels causally contribute to cardiovascular risk. They used PCSK9 genotype and HMGCR genotype as instrumental variables to prove causality from association data. Surprisingly we observed discordant associations between PCSK9 and HMGCR genotypes with mortality of sepsis. This logically leads to the novel conclusion that low LDL levels are not causal but rather LDL clearance rate contributes to sepsis mortality. 

In interim summary, PCSK9 regulates PL clearance and so inhibition of PCSK9 activity is an attractive sepsis and septic shock target - PCSK9 inhibitor(s) could be safe and effective.

**Modulation of HDL levels in sepsis**

The excessive host inflammation induced by PLs can lead to septic shock. Of all plasma lipoproteins, high-density lipoprotein (HDL) has the greatest affinity for PLs. Decreased HDL cholesterol (HDL-C) levels are associated with an increased risk of organ dysfunction and mortality, prolonged hospital admission, and nosocomial infection [15]. We wondered whether genetic variation in genes of HDL metabolism alter HDL-C levels and outcomes of sepsis. Cholesteryl ester transfer protein (CETP) regulates HDL levels and our major findings were that variants of CETP that decrease HDL increase mortality: more specifically, the rs1800777 GOF variant in CETP is associated with greater decline of HDL-C, elevated CETP activity, and increased mortality of sepsis. CETP also affected the development of acute kidney injury in septic shock [16].

Therefore, CETP activity plays a critical role in affecting sepsis outcomes. Thus, CETP could be a therapeutic target in sepsis. One CETP inhibitor, torcetrapib significantly increased the risk of infection- and malignancy-associated mortality in patients with high cardiovascular risk. This may be an off-target effect of torcetrapib because other large RCTs of CETP inhibitors dalcetrapib, evacetrapib, anacetrapib did not validate increased risk of severe infection. 

CETP regulation of HDL-C levels during acute infection critically modulates mortality and acute kidney injury. CETP genotype may represent a novel tool to risk stratify patients with sepsis. Modulation of HDL-C levels by CETP inhibition could be a novel sepsis therapeutic target.

**Immune enhancing strategies for patients in later sepsis who have signs of depressed immunity**

The later stages of septic shock are characterized by depressed immunity and increased risk of nosocomial infection, organ dysfunction and death [17]. Accordingly, immune enhancing interventions could improve outcomes of such patients. There are ongoing RCTs of IL-7 and of XX that are designed to test the hypothesis that later intervention to enhance immunity could decrease mortality of sepsis and septic shock.

**Recombinant human thrombomodulin**

Sepsis-associated coagulopathy (SAC) is common in sepsis and septic shock, affecting about 2/3 of patients. Anticoagulation with activated protein C was successful in a pivotal RCT but not in a later Phase 3 RCT in septic shock [18]. Recombinant human throbomodulin (rhTM) is a protein that binds to protein C and activates protein C, so rhTM is anticoagulant but also has anti-inflammatory and complement inhibitory actions. A Phase 2 RCT of rhTM showed that rhTM improved outcomes in patients who had increased INR and thrombocytopenia. Accordingly, such patients were included in a subsequent pivotal Phase 3 RCT that was recently completed.

References

1. Rhodes, A.; Evans, L.E.; Alhazzani, W.; Levy, M.M.; Antonelli, M.; Ferrer, R.; et al. Surviving Sepsis Campaign: International Guidelines for Management of Sepsis and Septic Shock: 2016. *Crit. Care Med.*
**2017**, *45*, 486–552.
2. Annane, D.; Renault, A.; Brun-Buisson, C.; Megarbane, B.; Quenot, J.P.; Siami, S.; et al. Hydrocortisone plus Fludrocortisone for Adults with Septic Shock. *N. Engl. J. Med*. **2018**, *378*, 809–818.
3. Venkatesh, B.; Finfer, S.; Cohen, J.; Rajbhandari, D.; Arabi, Y.; Bellomo, R.; et al. Adjunctive Glucocorticoid Therapy in Patients with Septic Shock. *N. Engl. J. Med*. **2018**, *378*, 797–808.
4. Annane, D.; Sebille, V.; Charpentier, C.; Bollaert, P.E.; Francois, B.; Korach, J.M.; et al. Effect of treatment with low doses of hydrocortisone and fludrocortisone on mortality in patients with septic shock. *JAMA*
**2002**, *288*, 862–871.
5. Annane, D.; Pastores, S.M.; Rochwerg, B.; Arlt, W.; Balk, R.A.; Beishuizen, A.; et al. Guidelines for the diagnosis and management of critical illness-related corticosteroid insufficiency (CIRCI) in critically ill patients (Part I): Society of Critical Care Medicine (SCCM) and European Society of Intensive Care Medicine (ESICM) 2017. *Intensive Care Med*. **2017**, *43*, 1751–1763.
6. Dwivedi, D.J.; Grin, P.M.; Khan, M.; Prat, A.; Zhou, J.; Fox-Robichaud, A.E.; et al. Differential Expression of PCSK9 Modulates Infection, Inflammation, and Coagulation in a Murine Model of Sepsis. *Shock*
**2016**, 46, 672–680.
7. Topchiy, E.; Cirstea, M.; Kong, H.J.; Boyd, J.H.; Wang, Y.; Russell, J.A.; et al. Lipopolysaccharide Is Cleared from the Circulation by Hepatocytes via the Low Density Lipoprotein Receptor. *PLoS ONE*
**2016**, *11*, e0155030.
8. Walley, K.R.; Thain, K.R.; Russell, J.A.; Reilly, M.P.; Meyer, N.J.; Ferguson, J.F.; et al. PCSK9 is a critical regulator of the innate immune response and septic shock outcome. *Sci. Transl. Med*. **2014**, *6*, 258ra143.
9. Walley, K.R.; Francis, G.A.; Opal, S.M.; Stein, E.A.; Russell, J.A.; Boyd, J.H. The Central Role of Proprotein Convertase Subtilisin/Kexin Type 9 in Septic Pathogen Lipid Transport and Clearance. *Am. J. Respir. Crit. Care Med*. **2015**, *192*, 1275–1286.
10. Roveran Genga, K, Lo, Cody.; Cirstea, M.S.; Walley, K.R.; Russell, J.A.; Linder, A.; Francis, G.A.; Boyd, J.H. The impact of PCSK9 loss-of-function genotype on 1-year mortality and recurrent infection in sepsis survivors. *eBioMedicine*. **2018**, in press.
11. Boyd, J.H.; Fjell, C.D.; Russell, J.A.; Sirounis, D.; Cirstea, M.S.; Walley, K.R. Increased Plasma PCSK9 Levels Are Associated with Reduced Endotoxin Clearance and the Development of Acute Organ Failures during Sepsis. *J. Innate Immun*. **2016**, *8*, 211–220.
12. Vermont, C.L.; den Brinker, M.; Kakeci, N.; de Kleijn, E.D.; de Rijke, Y.B.; Joosten, K.F.; et al. Serum lipids and disease severity in children with severe meningococcal sepsis. *Critical care medicine*. **2005**, *33*, 1610–1615.
13. Ference, B.A.; Robinson, J.G.; Brook, R.D.; Catapano, A.L.; Chapman, M.J.; Neff, D.R.; et al. Variation in PCSK9 and HMGCR and Risk of Cardiovascular Disease and Diabetes. *N. Engl. J. Med*. **2016**, *375*, 2144–2153.
14. Wang, F.; Meyer, N.J.; Walley, K.R.; Russell, J.A.; Feng, R. Causal Genetic Inference Using Haplotypes as Instrumental Variables. *Genet Epidemiol*. **2016**, 40, 3–44.
15. Cirstea, M.; Walley, K.R.; Russell, J.A.; Brunham, L.R.; Genga, K.R.; Boyd, J.H. Decreased high-density lipoprotein cholesterol level is an early prognostic marker for organ dysfunction and death in patients with suspected sepsis. *J. Crit. Care*
**2017**, 38, 289–294.
16. Roveran Genga K, Trinder, M.; Kong, H.J.; Leung, A.K.K.; Shimada, T.; Walley, K.R.; Russell, J.A.; Brunham, L.; Francis, G.A.; Boyd, J.H. Cholesterylester Transfer Protein (CETP) genotype R468Q is associated with increased risk of sepsis-associated Acute Kidney Injury (AKI). *Sci Reports*
**2018**, in press
17. Boomer, J.S.; To, K.; Chang, K.C.; Takasu, O.; Osborne, D.F.; Walton, A.H.; et al. Immunosuppression in patients who die of sepsis and multiple organ failure. *JAMA*
**2011**, *306*, 2594–2605.
18. Ranieri, V.M.; Thompson, B.T.; Barie, P.S.; Dhainaut, J.F.; Douglas, I.S.; Finfer, S.; et al. Drotrecogin alfa (activated) in adults with septic shock. *N. Engl. J. Med*. **2012**, *366*, 2055–2064.

#### **2.5.2. Blood Coagulation in Sepsis and ARDS** 

Camprubí-RimblasMartaInstitut d’ Investigació i Innovació Parc Taulí (I3PT), Sabadell, Catalonia, Spain Universitat Autònoma de Barcelona, Bellaterra, Catalonia, Spain

Sepsis and acute respiratory distress syndrome (ARDS) are life threatening diseases present in all critical care units with an elevated mortality and morbidity. Although significant advances have been performed in the management of patients with sepsis and ARDS, an effective pharmacological therapy is not available yet. 

In the first stages of ARDS proinflammatory mediators inhibit natural anticoagulant factors which alter the normal balance between coagulation and fibrinolysis leading to a procoagulant state [1]. This together with the breakdown of the alveolar-capillary barrier leads to proteinaceous edema, neutrophils infiltration into the alveolar compartment and the activation of macrophages towards a pro-inflammatory phenotype.

Beneficial effects of anti-coagulants have been proved in pre-clinical and clinical models of acute lung injury (ALI) and ARDS, although systemic bleeding offset its positive effects. Anti-coagulants could be effective for their anti-inflammatory activity in addition to their anti-coagulant properties. Moreover, given the cross talk of these pathways and their influence on permeability, anti-coagulants could also restore the alveolar-capillary barrier. 

Recent studies revealed that local administration of anticoagulants by nebulization could not only re-establish coagulant activity in the lung but also prevent systemic bleeding [2]. Beneficial effects of anticoagulants are shown in preclinical and clinical trials of ALI and ARDS, although results are controversial. 

Local administration of tissue-type plasminogen activator or tissue factor pathway inhibitor by nebulization could maintain its properties while avoid systemic adverse effects; however further investigation in this form of delivery is needed [3,4]. 

Nebulized heparin and/or antithrombin reduced pulmonary inflammation and coagulation and avoided systemic bleeding in a model of ALI [5]. Treatment with nebulized heparin modulated alveolar macrophages diminishing TGF-β and NF-κB effectors and the coagulation pathway and reduced the recruitment of neutrophils into the alveolar space. Nebulization of antithrombin alone ameliorated coagulation, while combined antithrombin and heparin had a higher impact reducing permeability and decreasing the infiltration of macrophages into the alveolar compartment. In injured human cell lung populations isolated from lung biopsies, heparin reduced the expression of pro-inflammatory markers in alveolar macrophages and deactivated the NF-κB pathway in alveolar type II cells; decreasing the expression of its mediators and effectors [6]. Also, in injured alveolar type II cells the administration of antithrombin decreased the levels of pro-inflammatory mediators and increased the tight junctions. Those studies proved a translational action of both anti-coagulants into humans.

The local administration of heparin and antithrombin by nebulization might be a potential treatment for ARDS, as they act in different pathways and processes of the pathophysiology of this syndrome. Lung injury is attenuated by the administration of those local anti-coagulants decreasing inflammation, coagulation and proving ameliorations on permeability without causing systemic bleeding. 

Nevertheless, controversial results have been found in clinical studies where heparin has been nebulized. In patients with ARDS nebulized heparin reduced the days of mechanical ventilation and did not affect systemic coagulation, although a trend to increase aPTT levels was observed with the highest dose [7]. In the multicenter trial HEPBURN, nebulized heparin in burn patients with inhalation trauma, the study had to be stopped prematurely due to safety reasons: increased blood sputum and high levels of aPTT [3]. 

To identify subtypes in ARDS heterogeneity might improve the response of those patients to a specific treatment. The nebulization of antithrombin and heparin combined or alone in a subtype of patients most likely to respond to the appropriate anticoagulant should also be studied. This together with the proper time to initiate a treatment is of major importance to fight ARDS. Moreover, we should have in mind that animal models mimic human ARDS only in part, and that this could affect the relevance of the data. 

ARDS is a really complex disease regarding its pathophysiology, so the unique or combined therapy should face different pathways and processes to ameliorate patient’s outcomes.

References

1. Ware, L.B.; Camerer, E.; Welty-Wolf, K.; Schultz, M.J.; Matthay, M.A. Bench to bedside: targeting coagulation and fibrinolysis in acute lung injury. *Am. J. Physiol. Lung Cell Mol. Physiol*. **2006**, 291, L307–311.
2. Hofstra, J.J.; Cornet, A.D.; de Rooy, B.F.; Vlaar, A.P.; van der Poll, T.; Levi, M.; Zaat, S.A.; Schultz, M.J. Nebulized antithrombin limits bacterial outgrowth and lung injury in *Streptococcus pneumoniae* pneumonia in rats. *Crit. Care*. **2009**, *13*, R145
3. Juschten, J.; Tuinman, P.R.; Juffermans, N.P.; Dixon, B.; Levi, M.; Schultz, M.J. Nebulized anticoagulants in lung injury in critically ill patients-an updated systematic review of preclinical and clinical studies. *Ann. Transl. Med*. **2017**, *5*, 444.
4. Camprubí-Rimblas, M.; Tantinyà, N.; Bringué, J; Guillamat-Prats, R.; Artigas, A. Anticoagulant therapy in acute respiratory distress syndrome. *Ann. Transl. Med*. **2018**, *6*, 36.
5. Chimenti, L.; Camprubí-Rimblas, M.; Guillamat-Prats, R.; Gomez, M.N.; Tijero, J.; Blanch, L.; Artigas A. Nebulized Heparin Attenuates Pulmonary Coagulopathy and Inflammation through Alveolar Macrophages in a Rat Model of Acute Lung Injury. *Thromb. Haemost*. **2017**, *117*, 2125–2134.
6. Camprubí-Rimblas, M.; Guillamat-Prats, R.; Lebouvier, T.; Bringué, J.; Chimenti, L.; Iglesias, M.; Obiols, C.; Tijero, J.; Blanch, L.; Artigas, A. Role of heparin in pulmonary cell populations in an in-vitro model of acute lung injury. *Respir. Res*. **2017**, 18, 89.
7. Dixon, B.; Schultz, M.J.; Smith, R. et al. Nebulized heparin is associated with fewer days of mechanical ventilation in critically ill patients: A randomized controlled trial. *Crit. Care Lond. Engl*. **2010**, *14*, R180.
Friday, 8 February 2019

#### **2.5.3. Clinical Year in Review: Respiratory Infections 2018–2019** 

NiedermanMichael S.MDProfessor of Clinical Medicine, Weill Cornell Medical College, Associate Chief, Division of Pulmonary and Critical Care Medicine, New York Presbyterian/Weill Cornell Medical Center, New York, NY, USA

Key articles to discuss include: 

1. Waterer, G.E.; Self, W.H.; et al. In-Hospital Deaths Among Adults With Community-Acquired Pneumonia. *Chest* **2018** 154, 628–635. 

2320 CAP patients in five tertiary care medical centers with 2.2% mortality. Concluded that most in hospital CAP deaths are NOT preventable by improved hospital care and that deaths are determined by age, comorbidity and end of life limitations. 

2. van Duijn, P.J.; Verbrugghe, W.; et al. The effects of antibiotic cycling and mixing on antibiotic resistance in intensive care units: a cluster-randomised crossover trial. *Lancet Infect. Dis*. **2018**, *18*, 401–409.

Cluster randomized, crossover trial of eight ICUs comparing antibiotic cycling with antibiotic mixing. No impact of cycling on preventing the carriage of drug resistant Gram –negative bacteria. 

3. Kunze-Szikszay, N.; Walliser, K.; Luther, J.; et al. Detecting Early Markers of Ventilator-Associated Pneumonia by Analysis of Exhaled Gas. *Critical Care Med* (in press). 

Animal study of the feasibility of using exhaled gas analysis for volatile organic compounds to detect the presence of early VAP, and possibly to distinguish one bacterial etiology from another. 

4. Wafa Ibn Saied, W.; Mourvillier, B.; et al. A Comparison of the Mortality Risk Associated With Ventilator-Acquired Bacterial Pneumonia and Nonventilator ICU-Acquired Bacterial Pneumonia. *Critical Care Med*. (in press).

Study of the impact of VAP and ICU acquired HAP on 30 days mortality. Although VAP was more common, both were associated with increased 30 days mortality, with a greater impact of HAP than of VAP. 

5. Cowley, M.C.; Ritchie, D.J.; et al. Outcomes Associated With De-escalating Therapy for Methicillin-Resistant Staphylococcus aureus in Culture-Negative Nosocomial Pneumonia. *Chest* **2019**, *155*, 53–59.

Study of the impact of stopping MRSA therapy in VAP patients with negative respiratory cultures. Of the approximately one third who were deescalated, there was no increase in mortality, but there was a shorter length of hospital and ICU stay and a lower incidence of acute kidney injury (less vancomycin use).

6. Six, S.; Rouze, A.; et al. Impact of hyperoxemia on mortality in critically ill patients with ventilator-associated pneumonia. *Ann. Transl. Med*. **2018**, *6*, 417.

Time spent with hyperoxemia (>98% saturation) prior to pneumonia onset, had no impact on VAP mortality in 93 patients. There was also no impact on ventilator free days or ICU length of stay. 

Welte, T.; et al. Efficacy and safety of trimodulin, a novel polyclonal antibody preparation, in patients with severe community-acquired pneumonia: a randomized, placebo-controlled, double-blind, multicenter, phase II trial (CIGMA study). *Intensive Care Med*. **2018**, *44*, 438–448.

Double blind trial showing no clinical benefit of IgM enriched immunoglobulin preparation in patients with severe CAP. In subset analysis, there may be a mortality benefit for those with either high CRP, low IGM, or both. 

8. Valade, S.; et al. Severe atypical pneumonia in critically ill patients: a retrospective multicenter study. *Ann. Intensive Care* **2018**, *8*, 81.

The 104 patients in ICU with atypical pneumonia, 73% Mycoplasma and 27% Chlamydophila, but with lower mortality than pneumococcus and maybe with some distinct clinical features.

9. Quah, J.; et al. Impact of microbial Aetiology on mortality in severe community-acquired pneumonia. *BMC Infectious Diseases* **2018**, *18*, 451.

In 117 patients in the ICU with CAP, 72% had a defined etiology. Isolated bacterial and viral infections occurred in slightly less than 30% each, and mixed bacterial –viral infection in 15%. Mixed infection was associated with a nearly 14-fold increase in mortality.

### 2.6. SESSION VI. SEVERE COMMUNITY-ACQUIRED PNEUMONIA

#### **2.6.1. Epidemiology and Outcome of Severe Community-Acquired Pneumonia** 

FerrerMiquelServei de Pneumologia, Hospital Clinic, Universitat de Barcelona, Villarroel 170, 08036 Barcelona, Spain. Institut d’Investigacions Biomèdiques August Pi i Sunyer (IDIBAPS) Centro de Investigación Biomedica En Red-Enfermedades Respiratorias (CibeRes, CB06/06/0028); miferrer@clinic.cat

**Keywords:** Severe community-acquired pneumonia; corticosteroids; systemic inflammatory response

**Support statement:** 2017-SGR-787, IDIBAPS, CibeRes (CB06/06/0028)-ISCIII.

Severe community-acquired pneumonia (CAP) has been defined as those cases that require intensive care unit (ICU admission. Direct admission to an ICU is required for patients with septic shock or acute respiratory failure requiring invasive mechanical ventilation (IMV), which are defined as major severity criteria by the 2007 IDSA/ATS guidelines [1]. These guidelines developed a set of 9 minor severity criteria on the basis of data on individual risks to identify patients with severe CAP: respiratory rate > 30 breaths/min, PaO_2_/FiO_2_ < 250 mmHg, multilobar infiltrates, confusion and/or disorientation, uremia (BUN level > 20 mg/dL), leukopenia (WBC count < 4 × 10^9^ cells/L), thrombocytopenia (platelet count < 100 × 10^9^ platelets/L), hypothermia (core temperature < 36 ºC), and hypotension (SBP < 90 mm Hg requiring aggressive fluid resuscitation). Admission to an ICU was also recommended for patients with 3 or more of these minor severity criteria [1]. However, none of those minor severity criteria adequately distinguish patients for whom ICU admission is necessary. Invasive mechanical ventilation was the main determinant for ICU admission in one study, followed by septic shock [2]. In the absence of major criteria, ICU admission was not related to survival of patients with minor severity criteria in this study [2]. Minor criteria probably identify patients that may require close monitoring rather than active life-support treatment.

Severe CAP is present in about 18% of hospitalized patients with CAP [3]. Despite global efforts to improve outcomes, mortality remains high in severe CAP. Considering all cases who met criteria for severe CAP, admitted or not to the ICU, we recently reported that the 30-day mortality of these patients is 22% [3]. Between 37% and 60% patients with CAP in the ICU may require IMV [2,4–6]. The mortality rates of ICU patients with CAP range between 13% and 28%, depending on the different series and whether ICU or hospital mortality was reported. 

The mortality rate of patients with severe CAP that require IMV is high, 32% and 55% for ICU mortality [7,8], and between 33% and 56% for hospital mortality [3,9,10]. Even in patients with CAP treated with NIV, the hospital mortality of those intubated after NIV failure may be as high as 54% [11]. As expected, older age, co-morbidities, and higher severity indices of pneumonia and organ system dysfunction at admission were independently associated with mortality in these reports. In order to assess whether the use of IMV is simply a marker of more acute severe disease or a determinant of poor outcome, we recently reported a large, prospective and consecutive series of hospitalized patients with severe CAP with special focus in the association of IMV with mortality [3]. Compared to non-intubated patients, those who received IMV did not present higher severity scores at hospital admission. However, the use of IMV independently predicted 30-day mortality. The contribution of IMV to mortality was reinforced by the finding that the actual mortality of these patients was substantially higher than that predicted by the APACHE-II score. In contrast, the actual mortality of non-intubated patients was lower than that predicted by this score. Whatever the cause is, the use of IMV seems to give a surplus of mortality in this subgroup of severe CAP patients.

Septic shock was also an independent predictor of mortality in patients with severe CAP in our series [3]. This is not surprising considering that shock is an accepted major severity criterion of CAP and that it is associated with clinical failure [12]. However, the actual mortality of those patients with septic shock as the unique major severity criterion was only slightly higher than that predicted by the APACHE-II score [3]. Finally, the hospital mortality of patients with at least one major severity criteria, either septic shock, need for IMV or both, was higher than that of patients with minor criteria only (86, 29% vs. 59, 16%, *p* < 0.001) [3].

*Streptococcus pneumoniae* is the leading cause of CAP; it is the underlying aetiological agent in 22% of patients requiring ICU admission [13], and about 30% of these patients develop pulmonary complications during their clinical courses [14]. A French multicentre study of severe pneumococcal CAP patients admitted to ICUs reported an overall mortality rate of 29% [15]. The high mortality of severe CAP occurs despite the fact that the majority of patients receive an early and adequate antibiotic treatment [3]. This is probably due, in part, to an imbalanced and disproportionate local and systemic inflammatory response that contributes to impairment of gas exchange, sepsis and end-organ dysfunction. Although clinical trials are heterogeneous, the adjuvant use of corticosteroids appears to be beneficial for those patients with severe CAP, particularly in presence of high systemic inflammatory response [16,17].

Acute respiratory distress syndrome (ARDS) is a potential complication of severe CAP. Pneumonia is largely the most frequent cause of ARDS in a multicentre prospective epidemiological study [18]. There was limited information regarding the incidence of ARDS, associated pathogens, risk factors, and specific outcomes in hospitalized patients with severe CAP in the era of the current Berlin definition [19], according to which patients must be receiving positive-pressure ventilation. We therefore assessed prospectively the characteristics of mechanically-ventilated patients with severe CAP and ARDS [20]. Among patients with CAP, ARDS was present in 2% of hospitalized patients, in 13% of ICU patients, and in 29% of mechanically ventilated patients, either invasively or non-invasively. Higher organ system dysfunction and previous antibiotic use were independent risk factors for ARDS, while previous inhaled corticosteroids was independently associated with a lower risk. The 30-day mortality was similar between patients with and without ARDS (25% vs. 30%, *p* = 0.25), confirmed by propensity-adjusted multivariate analysis. These results indicate that the expected association of ARDS with mortality seems more related to the need for mechanical ventilation in these patients with CAP rather than ARDS itself, as we have recently reported that invasive mechanical ventilation in patients with severe CAP independently predicts mortality [3].

References

1. Mandell, L.A.; Wunderink, R.G.; Anzueto, A.; Bartlett, J.G.; Campbell, G.D.; Dean, N.C.; Dowell, S.F.; File, T.M. Jr.; Musher, D.M.; Niederman, M.S.; Torres, A.; Whitney, C.G. Infectious Diseases Society of America/American Thoracic Society consensus guidelines on the management of community-acquired pneumonia in adults. *Clin. Infect. Dis.*
**2007**, *44*, S27-S72.
2. Liapikou, A.; Ferrer, M.; Polverino, E.; Balasso, V.; Esperatti, M.; Piner, R.; Mensa, J.; Luque, N.; Ewig, S.; Menendez, R.; Niederman, M.S.; Torres, A. Severe Community-Acquired Pneumonia: Validation of the Infectious Diseases Society of America/American Thoracic Society Guidelines to Predict an Intensive Care Unit Admission. *Clin. Infect. Dis*. **2009**, 48, 377–385.
3. Ferrer, M., Travierso, C.; Cilloniz, C.; Gabarrus, A.; Ranzani, O.T.; Polverino, E.; Liapikou, A.; Blasi, F.; Torres, A. Severe community-acquired pneumonia: Characteristics and prognostic factors in ventilated and non-ventilated patients. *PLoS ONE*
**2018**, *13*,e0191721.
4. Leroy, O.; Santre, C.; Beuscart, C.; Georges, H.; Guery, B.; Jacquier, J.M.; Beaucaire, G. A five-year study of severe community-acquired pneumonia with emphasis on prognosis in patients admitted to an intensive care unit. *Intensive Care Med.*
**1995**
*21*, 24–31.
5. Bodi, M.; Rodriguez, A.; Sole-Violan, J.; Gilavert, M.C.; Garnacho, J.; Blanquer, J.; Jimenez, J.; de la Torre, M.V.; Sirvent, J.M.; Almirall, J.; et al. Antibiotic prescription for community-acquired pneumonia in the intensive care unit: impact of adherence to Infectious Diseases Society of America guidelines on survival. *Clin. Infect. Dis.*
**2005**, *41*, 1709–1716.
6. Restrepo, M.I.; Mortensen, E.M.; Velez, J.A.; Frei, C.; Anzueto, A. A comparative study of community-acquired pneumonia patients admitted to the ward and the ICU. *Chest*
**2008**, *133*, 610–617.
7. Tejerina, E.; Frutos-Vivar, F.; Restrepo, M.I.; Anzueto, A.; Palizas, F.; Gonzalez, M.; Apezteguia, C.; Abroug, F.; Matamis, D.; Bugedo, G.; Esteban, A. Prognosis factors and outcome of community-acquired pneumonia needing mechanical ventilation. *J. Crit. Care*
**2005**, *20*, 230–238.
8. Aydogdu, M.; Ozyilmaz, E.; Aksoy, H.; Gursel, G.; Ekim, N. Mortality prediction in community-acquired pneumonia requiring mechanical ventilation; values of pneumonia and intensive care unit severity scores. *Tuberk Toraks.*
**2010**, *58*, 25–34.
9. Pascual, F.E.; Matthay, M.A.; Bacchetti, P.; Wachter, R.M. Assessment of prognosis in patients with community-acquired pneumonia who require mechanical ventilation. *Chest*
**2000**, *117*, 503–512.
10. Lee, J.H.; Ryu, Y.J.; Chun, E.M.; Chang, J.H. Outcomes and prognostic factors for severe community-acquired pneumonia that requires mechanical ventilation. *Korean J. Intern. Med*. **2007**, *22*, 157–163.
11. Carrillo, A.; Gonzalez-Diaz, G.; Ferrer, M.; Martinez-Quintana, M.E.; Lopez-Martinez, A.; Llamas, N.; Alcazar, M.; Torres, A. Non-invasive ventilation in community-acquired pneumonia and severe acute respiratory failure. *Intensive Care Med.*
**2012**, 38, 458–466.
12. Aliberti, S.; Amir, A.; Peyrani, P.; Mirsaeidi, M.; Allen, M.; Moffett, B.K.; Myers, J.; Shaib, F.; Cirino, M.; Bordon, J.; Blasi, F.; Ramirez, J.A. Incidence, etiology, timing, and risk factors for clinical failure in hospitalized patients with community-acquired pneumonia. *Chest*
**2008**, 134, 955–962.
13. Cilloniz, C.; Ewig, S.; Polverino, E.; Marcos, M.A.; Esquinas, C.; Gabarrus, A.; Mensa, J.; Torres, A. Microbial aetiology of community-acquired pneumonia and its relation to severity. *Thorax*
**2011**, *66*, 340–346.
14. Cilloniz, C.; Ewig, S.; Polverino, E.; Munoz-Almagro, C.; Marco, F.; Gabarrus, A., Menendez, R.; Mensa, J.; Torres, A. Pulmonary complications of pneumococcal community-acquired pneumonia: incidence, predictors, and outcomes. *Clin. Microbiol. Infect.*
**2012**, 18, 1132–1142.
15. Mongardon, N.; Max, A.; Bougle, A.; Pene, F.; Lemiale, V.; Charpentier, J.; Cariou, A.; Chiche, J.D.; Bedos, J.P.; Mira, J.P. Epidemiology and outcome of severe pneumococcal pneumonia admitted to intensive care unit: a multicenter study. *Crit. Care*
**2012**, *16*, R155.
16. Torres, A.; Sibila, O.; Ferrer, M.; Polverino, E.; Menendez, R.; Mensa, J.; Gabarrus, A.; Sellares, J.; Restrepo, M.I.; Anzueto, A.; Niederman, M.S.; Agusti, C. Effect of corticosteroids on treatment failure among hospitalized patients with severe community-acquired pneumonia and high inflammatory response: a randomized clinical trial. *JAMA*
**2015**, *313*, 677–686.
17. Torres, A.; Ferrer, M.; Niederman, M.S. Adjuvant therapies in critical care: steroids in community-acquired pneumonia. *Intensive Care Med*
**2018**, *44*, 478–481.
18. Bellani, G.; Laffey, J.G.; Pham, T.; Fan, E.; Brochard, L.; Esteban, A.; Gattinoni, L.; van, H.F.; Larsson, A.; McAuley, D.F.; et al. Epidemiology, Patterns of Care, and Mortality for Patients With Acute Respiratory Distress Syndrome in Intensive Care Units in 50 Countries. *JAMA*
**2016**, *315*,788–800.
19. Ranieri, V.M.; Rubenfeld, G.D.; Thompson, B.T.; Ferguson, N.D.; Caldwell, E.; Fan, E.; Camporota, L.; Slutsky, A.S. Acute respiratory distress syndrome: the Berlin Definition. *JAMA*
**2012**, *307*, 2526–2533.
20. Cilloniz, C.; Ferrer, M.; Liapikou, A.; Garcia-Vidal, C.; Gabarrus, A.; Ceccato, A.; Puig de la, B.J.; Blasi, F.; Torres, A. Acute respiratory distress syndrome in mechanically ventilated patients with community-acquired pneumonia. *Eur. Respir. J.*
**2018**, 51.

#### **2.6.2. Severe CAP: How to Improve Outcomes** 

NiedermanMichael S.MDProfessor of Clinical Medicine Weill Cornell Medical College; Associate Chief, Division of Pulmonary and Critical Care Medicine. New York Presbyterian/Weill Cornell Medical Center, New York, USA

This presentation will review strategies including

- Early recognition of severe illness: q-SOFA may be the best mortality predictor, but M-SOFA is better for predicting ICU admission.
  Ranzani, O.T.; et al. *AJRCCM* **2017**; *196*, 1287–97.
- Proper site of care and deciding need for cardiac monitoring
  – Severity assessment, biomarkers
  – Cardiac disease and impact on long-term CAP mortality
    Reyes, L.F.; et al. *AJRCCM* **2017**, 196, 609–620.
- Supportive care: HFNC
- Proper antibiotic selection
  – Advantages for adding a macrolide to a beta-lactam
  – Pereira, J.M.; et al. *J. Crit. Care* **2018**, 43, 183–189.
  – Ceccato, A.; et al. *Chest* **2019**, (in press). (benefit in pneumococcal CAP, esp with a high inflammatory response).
- Adjunctive Therapy
  – Steroids: Yes for severe CAP, but NO for influenza
  – Immunoglobulins? IgM enriched preparations and benefit in subsets with high CRP and low IgM.
- Welte, T.; et al. *Intensive Care Med*. **2018**, *44*, 438–448.
  – Others: Statins, aspirin

### 2.7. SESSION VII. THE MICROBIOME IN CRITICAL ILLNESS

#### **2.7.1. The Microbiome in the ICU and Implications for Selective Decontamination Strategies** 

BosLieuweAmsterdam, The Netherlands

The human microbiome consists of a similar amount of cells as the human body itself and has about 100 times the number of genes. The microbiome changes considerably during critical illness and the composition is much more chaotic than under healthy circumstances. This may explain the much higher rectal carriage of potentially pathogenic micro-organisms, which is associated with more bloodstream infections and pneumonia. A loss of diversity of the microbiome is associated with infection and a worse outcome and therefore the microbiome may be a target of interventions. Selective decontamination of the digestive tract has a long history in the ICU and is considered standard of practice in several hospitals throughout Europe. Through the application of topic antibiotics in the digestive tract, SDD aims to reduce the burden of potentially pathogenic micro-organisms and thereby reduce the number of bloodstream and respiratory infections. Several studies that randomized more than 10.000 patients showed that SDD is associated with less respiratory tract infections and bloodstream infections. It also resulted in a reduction in mortality with an adjusted odds ratio around 0.80. This makes SDD one of the most effective and well studied interventions in the intensive care unit. However, adoption of SDD as a standard practice has met a lot of resistance from clinicians. We will explore the five most frequently used arguments against SDD in the lecture.

#### **2.7.2. Ways to Prevent the Acquisition of Multi-Resistant Bacteria (Mrb) in the Gut Microbiota of ICU Patients** 

CarletJeanConsultant, President of the World Alliance Against Antibiotic Resistance (WAAAR). Paris, France

Antibiotic resistance is becoming a serious threat for humanity. Antimicrobial resistance (AMR) is far higher in the ICUs than in classical wards in the hospital or in the community. Particularly in ICU patients, the gut can be considered as the epicenter or the factory of antibiotic resistance [1]. The presence of MRB in the gut is due to either their selection by the antibiotics or to the transmission of MRB from other patients or the environment via the hands. The ways to prevent the acquisition of those resistant strains in the gut of ICU patients are usually hygiene control measures, in particular hand-disinfection and antibiotic restriction/stewardship. However, some other techniques might be useful to prevent colonization of the gut with MRB, or to clean up the gut of the patients from MRB, in particular in case of outbreaks in the ICU from those strains. Selective Digestive Decontamination (SDD) including the use of non-absorbable antibiotics in the stomach is one of those techniques. This technique allows to reach extremely high levels of those non- absorbable antibiotics, far above the MIC of all strains of G- including the most resistant ones. The topic is very controversial, since SDD is suspected to increase AMR in the ICU, but in fact, some studies from The Netherlands showed that resistance was not increased in the digestive flora with SDD, but rather decreased [2]. However some increase in the MIC of aminoglycosides to gram negative has been described, some strains being fully resistant to those antibiotics. Resistance to colistin has been also described. Oral non-absorbable antibiotics, which can be used without the other components of SDD have also been able to stop outbreaks with MRB in the ICU, for example one due a highly resistant strain of Klebsiella pneumoniae [3]. Several recent studies confirm this initial one. However, some studies are negative, and further data are mandatory.

Other methods have also been tried with contrasting results, including the use of probiotics, of B- lactamases able to destroy MRB, of encapsulated charcoal able to trap antibiotics at the end of the small intestine and then avoid their effect on the colonic flora [4], and more recently fecal transplantation with very promising results [5]. The “search and destroy “concept has been developed a long time ago in The Netherlands with excellent results, in particular for Staphylococcus aureus resistant to meticillin. On the same line, the concept “Search, destroy and restore” could be one of the best solution to treat gut colonization with MRB in the ICU. The issue of the prevention must of course be part of this strategy.

References

1. Carlet, J. The gut is the epicenter of antibiotic resistance. *Antibio. Resist. Infect. Control*
**2012**, 2739, doi:10.1186/2047-2994-1-39.
2. Oostdijk, E.A.; Kesecioglu, J.; Schulz, M.J.; et al. Effects of decontamination of the oropharynx and intestinal tract on antibiotic resistance in ICUs: A randomized clinical trial. *JAMA*
**2014**, *312*, 1429–1437.
3. Brun Buisson, C.; Legrand, P.; Rauss, A.; et al. Intestinal decontamination for control of nosocomial multi-resistant gram-negative bacilli. Study of an outbreak in an intensive care unit. *Ann. Intern. Med*. **1989**, *110*, 873–881
4. de Gunzburg, J.; Ghozlane, A.; Ducher, A.; Le Chatelier, E.; Duval, X.; Ruppé, E.; Armand-Lefevre, L.; Sablier-Gallis, F.; Burdet, C.; Alavoine, L.; Chachaty, E.; Augustin, V.; Varastet, M.; Levenez, F.; Kennedy, S.; Pons, N.; Mentré, F.; Andremont, A. Protection of the Human Gut Microbiome From Antibiotics. *J. Infect. Dis*. **2018**, *217*, 628–636. doi:10.1093/infdis/jix604.
5. Huttner, B.D.; de Lastours, V.; Wassenberg, M.; Maharshak, N.; Mauris, A.; Galperine, T.; Zanichelli, V.; Kapel, N.; Bellanger, A.; Olearo, F.; Duval, X.; Armand, L.; Carmeli, Y.; Bonten, M.; Fantin, B.; Harbarth, S.; R-Gnosis WP3 study group. A five-day course of oral antibiotics followed by faecal transplantation to eradicate carriage of multidrug-resistant Enterobacteriaceae: A Randomized Clinical Trial. *Clin. Microbiol. Infect*. **2019**, doi:10.1016/j.cmi.2018.12.009. [Epub ahead of print]

### 2.8. SESSION VIII. DIAGNOSTIC TOOLS IN PULMONARY INFECTION AND SEPSIS

#### **2.8.1. Diagnostic Tools in Pulmonary Infection and Sepsis: Caveats of Molecular Testing for Diagnosing Infection in the ICU** 

BouzaEmilioDept of Medicine. Universidad Complutense. CIBERES. Clin. Microbiology and ID Department. Hospital Gregorio Marañón. Madrid. Spain

The new diagnostic methods, based mainly on molecular technology, allow the detection of microorganisms in a much shorter period of time than those of traditional microbiology. In the field of respiratory infection in Intensive Care Units, new procedures have simplified the diagnosis of viruses, such as the case of Influenza or Respiratory Syncytial Virus [1–5]. Tests are available that make possible, not only the detection of bacteria, but also the quantification of the bacterial load and the rapid detection of various mechanisms of resistance. Multiplex-real time Polymerase Chain Reaction tests are now available commercially and ease to use. They identify the most common virus and Gram positive and Gram negative bacteria causing VAP in less than 4 h [6]. Rapid detection of the main resistance mechanisms, including Carbapenemase producing bacteria are now available in hours but most studies have been performed on isolated strains but not on direct clinical samples [7–9]. 

With regard to fungal diseases, the new technology permits rapid identification of fungi of the genus Aspergillus and the family Mucoraceae and has been greatly improved with the molecular detection techniques of *Pneumocystis jirovecii*. In addition to tests directly applied to respiratory samples, fungal blood biomarkers such as 1–3 B-D-glucan and Galactomannan are of unquestionable diagnostic help [10–12].

Speaking of time of response, as opposed to a traditional duration of 48–72 h to give answers of practical clinical interest, we have switched to clinically useful response times of less than 8 h. Despite of this, there are still not enough data to certify the clinical and economic impact of these tests, which in my opinion are, by far, cost-effective.

One of the problems lies, not in the technique itself, but in the mechanisms of explanation and communication to the clinician that are associated with it. With bad communication, the best techniques are incapable of making an adequate clinical impact [13]. However, the best markers to evaluate clinical impact of microbiological laboratory techniques have yet to be determined. 

A good example of our previous points is Infection with methicillin-resistant *S. aureus* (MRSA). Today the presence of MRSA genome can be reported in approximately 2 h. In recent years it has become acceptable that MRSA pneumonia can be reasonable rule out if a nasal carrier status is discarded. This obviously permit safe de-escalation of specific antiMRSA drugs with considerable stewardship impact [14]. 

Regarding etiological detection in the septic patient, the changes are also spectacular, although the new molecular technology has not succeeded in displacing traditional blood cultures as a reference technique [15,16]. Microbiology departments have an essential role in alerting to sepsis in the whole population of a general hospital. The simple receipt of a blood culture request is a valuable alert of sepsis, before microbiological results, and a telephone intervention with alert just upon reception of blood cultures has proven clinically useful [17]. 

A group of techniques that is making its way into the detection of specific microorganisms in a time close to 2 h, are the techniques of T2 Magnetic Resonance, available now, not only for the detection of candidemia but for the presence of several of the bacteria most frequently causing sepsis [18–22].

The direct detection of bacterial or fungal genomes in blood continues to be problematic and for this reason, an interesting way of working is to use the newly arrived blood cultures to identify microorganisms in other samples coming from places other than the blood that are sent simultaneously to the laboratory. In a proportion of those cases the causal microorganism of sepsis can be found in samples such as

urine, respiratory tract or skin and soft tissues in which, because of their higher microbial load, molecular techniques are more sensitive [23,24].

The problem of rapid diagnosis of Infectious Diseases is increasingly moving from the technique itself and its performance to administrative problems derived fundamentally from a simplistic and basic interpretation of its cost and availability problems derived from the times and working hours of the Microbiology Services. The pending revolution is that of having a 7 × 24 Microbiology, with the capacity to perform, interpret and transmit these results in real time [25].

References

1. Egilmezer, E.; Walker, G.J.; Bakthavathsalam, P.; Peterson, J.R.; Gooding, J.J.; Rawlinson, W.; et al. Systematic review of the impact of point-of-care testing for influenza on the outcomes of patients with acute respiratory tract infection. *Rev. Med.l Virol.*
**2018**, *28*, e1995.
2. Kim, H.J.; Choi, S.M.; Lee, J.; Park, Y.S.; Lee, C.H.; Yim, J.J.; et al. Respiratory virus of severe pneumonia in South Korea: Prevalence and clinical implications. *PLoS ONE*
**2018**, *13*, e0198902.
3. van Someren Greve, F.; Juffermans, N.P.; Bos, L.D.J.; Binnekade, J.M.; Braber, A.; Cremer, O.L.; et al. Respiratory Viruses in Invasively Ventilated Critically Ill Patients-A Prospective Multicenter Observational Study. *Critical Care Med*. **2018**, 46, 29–36.
4. Huang, H.S.; Tsai, C.L.; Chang, J.; Hsu, T.C.; Lin, S.; Lee, C.C. Multiplex PCR system for the rapid diagnosis of respiratory virus infection: systematic review and meta-analysis. Clinical microbiology and infection: the official publication of the European Society of Clinical Microbiology and Infectious Diseases. **2018**, *24*, 1055–1063.
5. Basile, K.; Kok, J.; Dwyer, D.E. Point-of-care diagnostics for respiratory viral infections. Expert review of molecular diagnostics. **2018**, *18*, 75–83.
6. Kollef, M.H.; Burnham, C.D. Ventilator-Associated Pneumonia: The Role of Emerging Diagnostic Technologies. Seminars in respiratory and critical care medicine. **2017**, *38*, 253–263.
7. Miller, S.; Humphries, R.M. Clinical laboratory detection of carbapenem-resistant and carbapenemase-producing Enterobacteriaceae. Expert review of anti-infective therapy. **2016**, *14*, 705–717.
8. Lutgring, J.D.; Limbago, B.M. The Problem of Carbapenemase-Producing-Carbapenem-Resistant-Enterobacteriaceae Detection. *J.Clin. Micro*. **2016**, *54*, 529–534.
9. Burillo, A.; Marin, M.; Cercenado, E.; Ruiz-Carrascoso, G.; Perez-Granda, M.J.; Oteo, J.; et al. Evaluation of the Xpert Carba-R (Cepheid) Assay Using Contrived Bronchial Specimens from Patients with Suspicion of Ventilator-Associated Pneumonia for the Detection of Prevalent Carbapenemases. *PLoS ONE*
**2016**, *11*, e0168473.
10. Timbrook, T.T.; Spivak, E.S.; Hanson, K.E. Current and Future Opportunities for Rapid Diagnostics in Antimicrobial Stewardship. *The Medical clinics of North America*. **2018**, *102*, 899–911.
11. Rath, P.M.; Steinmann, J. Overview of Commercially Available PCR Assays for the Detection of Aspergillus spp. DNA in Patient Samples. *Frontiers in microbiology*. **2018**, *9*, 740.
12. Richardson, M.; Page, I. Role of Serological Tests in the Diagnosis of Mold Infections. Current fungal infection reports. **2018**, *12*, 127–136.
13. Timbrook, T.T.; Morton, J.B.; McConeghy, K.W.; Caffrey, A.R.; Mylonakis, E.; LaPlante, K.L. The Effect of Molecular Rapid Diagnostic Testing on Clinical Outcomes in Bloodstream Infections: A Systematic Review and Meta-analysis. Clinical infectious diseases: an official publication of the Infectious Diseases Society of America. **2017**, *64*, 15–23.
14. Smith, M.N.; Brotherton, A.L.; Lusardi, K.; Tan, C.A.; Hammond, D.A. Systematic Review of the Clinical Utility of Methicillin-Resistant *Staphylococcus aureus* (MRSA) Nasal Screening for MRSA Pneumonia. The Annals of pharmacotherapy. **2019**, doi:1060028018823027.
15. van de Groep, K.; Bos, M.P.; Savelkoul, P.H.M.; Rubenjan, A.; Gazenbeek, C.; Melchers, W.J.G.; et al. Development and first evaluation of a novel multiplex real-time PCR on whole blood samples for rapid pathogen identification in critically ill patients with sepsis. *Eur. J. Clin. Microbiol. Infect. Dis.*
**2018**, *37*, 1333–1344.
16. Brenner, T.; Decker, S.O.; Grumaz, S.; Stevens, P.; Bruckner, T.; Schmoch, T.; et al. Next-generation sequencing diagnostics of bacteremia in sepsis (Next GeneSiS-Trial): Study protocol of a prospective, observational, noninterventional, multicenter, clinical trial. *Med*. **2018**, *97*, e9868.
17. Bunsow, E.; Gonzalez-Del Vecchio, M.; Sanchez, C.; Munoz, P.; Burillo, A.; Bouza, E. Improved Sepsis Alert With a Telephone Call From the Clinical Microbiology Laboratory: A Clinical Trial. *Med*. **2015**, *94*, e1454.
18. Clancy, C.J.; Nguyen, M.H. Non-Culture Diagnostics for Invasive Candidiasis: Promise and Unintended Consequences. *J. .Fungi* (Basel, Switzerland). **2018**, 4.
19. Clancy, C.J.; Pappas, P.G.; Vazquez, J.; Judson, M.A.; Kontoyiannis, D.P.; Thompson, G.R. 3^rd^.; et al. Detecting Infections Rapidly and Easily for Candidemia Trial, Part 2 (DIRECT2): A Prospective, Multicenter Study of the T2Candida Panel. *Clin. Infect. Dis*. **2018,**
*66*, 1678–1686.
20. Giannella, M.; Paolucci, M.; Roncarati, G.; Vandi, G.; Pascale, R.; Trapani, F.; et al. Potential role of T2Candida in the management of empirical antifungal treatment in patients at high risk of candidaemia: A pilot single-centre study. *The Journal of antimicrobial chemotherapy*. **2018**, *73*, 2856–2859.
21. Marco, F. Molecular methods for septicemia diagnosis. Enfermedades infecciosas y microbiologia clinica. **2017** 35, 586–592.
22. Muñoz, P.; Vena, A.; Machado, M.; Gioia, F.; Martinez-Jimenez, M.C.; Gomez, E; et al. T2Candida MR as a predictor of outcome in patients with suspected invasive candidiasis starting empirical antifungal treatment: a prospective pilot study. *J. Antimicrob. Chemoter*. **2018**, *73*.
23. Bouza, E.; Onori, R.; Semiglia-Chong, M.A.; Alvarez-Uria, A.; Alcala, L.; Burillo, A. Fast track SSTI management program based on a rapid molecular test (GeneXpert((R)) MRSA/SA SSTI) and antimicrobial stewardship*. J. Microbiol. Immunol*. **2018** (corrected proofs).
24. Burillo, A.; Rodriguez-Sanchez, B.; Ramiro, A.; Cercenado, E.; Rodriguez-Creixems, M.; Bouza, E. Gram-stain plus MALDI-TOF MS (Matrix-Assisted Laser Desorption Ionization-Time of Flight Mass Spectrometry) for a rapid diagnosis of urinary tract infection. *PLoS ONE*
**2014**, *9*, e86915.
25. Burillo, A.; Bouza, E. Use of rapid diagnostic techniques in ICU patients with infections. *BMC infectious diseases*. **2014,**
*14*, 593.

#### **2.8.2. Should we and how could we differentiate VAT from VAP** 

NseirSaadCritical Care Center, CHU Lille, F-59000 LilleLille University, Medicine Faculty, F-59000 Lille, France
s-nseir@chru-lille.fr


Ventilator-associated tracheobronchitis (VAT) is a common ICU-acquired infection. Its incidence ranges 1.4-19% of critically ill patients receiving invasive mechanical ventilation. This infection is considered as an intermediate process between colonization and ventilator-associated pneumonia (VAP). Histological studies showed a continuum between these two infections. Several definitions are available for VAT. However, all these definitions have some limitations. The most accepted and frequently used definition include all the following criteria: fever >38 °C with no other cause, purulent tracheal secretions, positive tracheal aspirate (≥105 cfu/mL), and absence of new infiltrate on chest X-ray [1]. VAT is frequently caused by Gram negative bacilli. *Pseudomonas aeruginosa,* Staphylococcus aureus, and *Acinetobacter baumannii* are the most common pathogens isolated in respiratory secretions of VAT patients.

Previous studies reported prolonged duration of mechanical ventilation, and ICU stay in VAT patients. This negative impact on outcome is related to increased inflammation of lower respiratory tract, and sputum production [2]. Extubation failure, and difficult weaning could result from increased sputum production. In addition, higher rates of VAP were reported in patients with VAT compared with those without VAT.

Differentiating VAT from colonization or from VAP could be a difficult task. The use of significant microbiological threshold (tracheal aspirate at 105 cfu/mL or bronchoalveolar lavage (BAL) at 104 cfu/mL), associated with local and systemic signs of infection could be helpful to distinguish VAT from tracheobronchial colonization. Further, in the event that a portable chest X-ray is not accurate enough in diagnosing a new infiltrate in critically ill patients, it would probably allow differentiating severe (VAP) from less severe (VAT) ventilator-associated lower respiratory tract infections. Therefore, one could argue that the presence of a new infiltrate on chest X-ray, associated with clinical and biological signs of infection, should be considered as a severity sign, that might trigger prompt empirical antibiotic treatment [3,4].

There are at least four reasons to suggest a continuum between VAT, and VAP. First, the higher rates of VAP in patients with VAT compared with those with no VAT. Second, histological findings of postmortem animal and human studies clearly showed the coexistence of these two infections, and described them as bronchopneumonia. Third, the higher SOFA, CPIS, PCT levels, and mortality in VAP compared with VAT patients strongly suggest that VAT might be a precursor of VAP. Fourth, the pathophysiology of VAP also supports this hypothesis, as microaspiration of contaminated oropharyngeal secretions is a permanent phenomenon, lesions with different severity might exist in the lower respiratory airway of mechanically ventilated patients. However, in some patients VAP might occur without previous VAT, suggesting two different pathogenic pathways.

There is probably an overlap between these two infections, but no available examination could differentiate them at bedside [5]. CT scan and lung ultrasound are more efficient in diagnosing lung infiltrate than chest Xray. However, to diagnose a new infiltrate, baseline examination is required. Additionally, fiberoptic bronchoscopy and BAL could probably not be used to differentiate VAT from VAP, as previous studies reported frequent high burden of bacteria on BAL in chronically ventilated patients without local or systemic signs of infection. A post-hoc analysis of the large TAVeM international database evaluated the interest of biomarkers in differentiating VAT from VAP [6]. Although PCT and CRP presented lower values in VAT as compared to VAP, there was a marked overlap of both biomarkers values in both VA-LRTI not allowing adequate discrimination. Another recent large post-hoc study evaluated the accuracy of the clinical pulmonary infection score (CPIS) in differentiating VAT from VAP [7]. All patients with a microbiologically confirmed VAT or VAP were included. CPIS exhibited moderate accuracy for the diagnostic of VAP among patients with microbiologically confirmed ventilator infections. 

There are at least two reasons to distinguish VAT from VAP: (1) antibiotic treatment for VAT is currently not recommended because of lack of good quality data in favor of antibiotic treatment and also because antibiotic treatment is a well-known risk factor for multidrug resistance bacteria emergence. (2) if antibiotic treatment is beneficial in VAP patients, a short course of antibiotics (i.e., 3 days) might be sufficient to treat this infection. The recent large prospective multicenter multinational TAVeM study allowed validation of a highly specific definition of VAT, and clearly showed that VAT and VAP are not associated with the same impact on outcome. Mortality rate was significantly higher in VAP patients compared with those with VAT, and those with no VA-LRTI. In our opinion, this is a key finding supporting the fact that these two infections should be differentiated even if closely linked, and that VAT patients might benefit from a shorter duration of antibiotic treatment. The TAVeM2, randomized double-blind controlled study, has started recently in France, and is evaluating the impact of two durations of systemic antibiotic treatment (3, or 7 days) versus no antibiotic treatment in a large cohort of VAT patients.

References

1. Martin-Loeches, I.; Povoa, P.; Rodríguez, A.; Curcio, D.; Suarez, D.; Mira, J.-P.; et al. Incidence and prognosis of ventilator-associated tracheobronchitis (TAVeM): a multicentre, prospective, observational study. *Lancet Respir. Med.*
**2015**, *3*, 859–868.
2. Nseir, S.; Di Pompeo, C.; Pronnier, P.; Beague, S.; Onimus, T.; Saulnier, F.; et al. Nosocomial tracheobronchitis in mechanically ventilated patients: Incidence, aetiology and outcome. *Eur. Respir J.*
**2002**, *20*, 1483–1489.
3. Keane, S.; Vallecoccia, M.S.; Nseir, S.; Martin-Loeches, I. How Can We Distinguish Ventilator-Associated Tracheobronchitis from Pneumonia? *Clin. Chest Med*. **2018**, *39*, 785–796.
4. Coelho, L.; Rabello, L.; Salluh, J.; Martin-Loeches, I.; Rodriguez, A.; Nseir, S.; et al. C-reactive protein and procalcitonin profile in ventilator-associated lower respiratory infections. *J. Crit Care*. **2018**, *48*, 385–389.
5. Chastre, J.; Luyt, C.-E. Does this patient have VAP? *Intensive Care Med*. **2016**. doi:10.1007/s00134-016-4239-1.
6. Martin-Loeches, I.; Coakley, J.D.; Nseir, S. Should We Treat Ventilator-Associated Tracheobronchitis with Antibiotics? *Semin Respir Crit Care Med*. **2017**, *38*.
7. Gaudet, A.; et al. *Intensive Care Med. Exp.*
**2018**, *6*, 0077.

#### **2.8.3. Cardiac Biomarkers in Community-Acquired Pneumonia** 

MenéndezRosarioUniversity and Polytechnic Hospital La Fe, Valencia, Spain

Community-acquired pneumonia may cause during the acute phase cardiovascular complications with a frequency around 15% and, interestingly, after discharge and during the next 10 years that risk remain higher [1]. The association between cardiovascular complications and pneumonia has been recently recognized and proved in several studies: 

1. In animal pneumonia models Streptococcus pneumonia may directly cause necrosis of myocites and scar formation [2] 

2. In case-control studies, after controlling for comorbid conditions and age, patients with pneumonia had significantly higher risk for developing cardiovascular events 

3. In sepsis, a similar increase of cardiovascular events has been reported during the episode and in those who survive. In fact, some authors have stated that pneumonia should be considered a *new* cardiovascular risk factor. During bacteremia *Streptococcus pneumoniae* can translocate across the vascular endothelium into the myocardium and form microlesions. Cardiotoxic products generated by *S. pneumoniae* may damage myocardium and heart failure and other complications may develop. Several molecular mechanisms underlying pneumococcal cardiac invasion have been described: cardiomyocite death, scar formation and cardiac remodeling. 

Biomarkers are important tools that allow quantifying biologic processes such as inflammation or damage with a potential usefulness for diagnosis and prognosis evaluation. Several criteria define a good biomarker: accuracy, reliability and impact for diagnosis. There are several cardiac biomarkers that have been evaluated in pneumonia with the purpose of measuring cardiovascular damage and/or mortality [3]. Thus, cardiac troponin is related to myocardial necrosis; endothelin 1 is secreted by endothelial cells and correlates with shear stress; natriuretic peptides of myocardial stress (proBNP and proANP) are considered of similar accuracy for the diagnosis of heart failure); proADM is a neurohormonal biomarker of pressure and volume overload in heart failure. 

In general, most of the studies have measured cardiac biomarkers at CAP diagnosis and there are scarce publications with determination after discharge. Moreover, mortality is the most frequent outcome whereas cardiovascular events have been less investigated. Kruger et al. [4] in a CAP cohort found that cardiac biomarkers, and especially proADM, were associated with short and medium term mortality (at 90 days). Biteker et al. [5] in a CAP prospective study evaluated echocardiographic findings along with NT-proBNP levels. They found that patients with raised levels of NT-proBNP and reduction of TAPSE had increased probability of complications and death. On the contrary, those with low levels of NT-proBNP without reduction of TAPSE had no complications. In a posterior study, endothelin 1 provided additional information when studied together with proBNP. 

Chang CL et al. [6] evaluated both NT-proBNP and troponin T for mortality prediction in CAP. They found that both, NT-proBNP and troponin T, predicted 30-day mortality in age-adjusted analysis. However, after adjustment for PSI, a raised NT-proBNP persisted as independent biomarker predictor. The authors pointed out that some degree of cardiac involvement may exist and even remain under-recognized during the pneumonia episode and its presence is related to poor outcome. Nowak et al. [7] have compared prognostic mortality accuracy (comparing areas under the curve) of natriuretic peptides in CAP, showing that it was similar for short and long-term mortality although not higher than Pneumonia severity index (PSI). In multivariable Cox-regression analysis, NT-proBNP remained an independent mortality predictor and they suggest that a combination of this biomarker with PSI would be helpful for stratifying mortality risk. 

Soluble markers of platelet activation such as s-P selectin and sCD40 ligand have been found in patients with stable atherosclerosis or acute coronary syndromes and they predict cardiovascular events. In CAP, Cangemi et al. [8] evaluated platelet activation and the development of myocardial infarction showing that raised platelet activation biomarkers were independent predictors. Recently, Mendez R et al. [9] have evaluated the usefulness of cardiac biomarkers in CAP to predict early and late cardiovascular events (1 year follow-up). They have reported that raised initial levels and at 30 days were independent predictors of cardiovascular events.

In summary, cardiac biomarkers as surrogate markers of cardiovascular damage or stress may help identifying CAP patients more susceptible to develop cardiovascular events and/or die. The increased damage and its persistence are pointing out to damage of the cardiovascular system provoked by CAP. 

References

1. Corrales-Medina, V.F.; Suh, K.N.; Rose, G.; Chirinos, J.A.; Doucette, S.; Cameron, D.W.; Fergusson, D.A. Cardiac complications in patients with community-acquired pneumonia: A systematic review and meta-analysis of observational studies. *PLoS Med.*
**2011**, *8*, e1001048.
2. Reyes, L.F.; Restrepo, M.I.; Hinojosa, C.A.; Soni, N.J.; Anzueto, A.; Babu, B.L.; Gonzalez-Juarbe, N.; Rodriguez, A.H.; Jimenez, A.; Chalmers, J.D.; Aliberti, S.; Sibila, O.; Winter, V.T.; Coalson, J.J.; Giavedoni, L.D.; Dela Cruz, C.S.; Waterer, G.W.; Witzenrath, M.; Suttorp, N.; Dube, P.H.; Orihuela, C.J. Severe Pneumococcal Pneumonia Causes Acute Cardiac Toxicity and Subsequent Cardiac Remodeling. *Am. J. Respir. Crit. Care Med.*
**2017**, *196*, 609–620.
3. Wang, J.; Tan, G.; Han, L.; Bai, Y.; He, M.; Liu, H. Novel biomarkers for cardiovascular risk. *J. Geriatr. Cardiol.*
**2017**, *14*, 135–150.
4. Krüger, S.; Ewig, S.; Giersdorf, S.; Hartmann, O.; Suttorp, N.; Welte, T.; Becker, M.; Kuhnke, A.; Lode, H.; Schmidt-Ioanas, M.; Bauer, T.; Schlosser, B.; Pletz, M.; Dalhoff, K.; Pischke, S.; Schübel, N.; Huntemann, I.; Lorenz, J.; Klante, T.; Schaberg, T.; Voigt, K.; Schumann, C.; Jany, B.; Ziegler, U.; Illmann, T.; Wallner, M.; Weber, M.; Von Baum, H.; Barten, G.; Gosmann, L. Cardiovascular and inflammatory biomarkers to predict short- and long-term survival in community-acquired pneumonia: Results from the German Competence Network, CAPNETZ. *Am. J. Resp Crit. Care Med*
**2010**; *182*, 1426–1434.
5. Biteker, F.S.; Basaran, O.; Dogan, V.; Caylak, S.D.; Yildirim, B.; Sozen, H. Prognostic value of transthoracic echocardiography and biomarkers of cardiac dysfunction in community-acquired pneumonia. *Clin. Microbiol. Infect.*
**2016**, *2*2, 1006.e1–1006.e6.
6. Chang, C.L.; Mills, G.D.; Karalus, N.C.; Jennings, L.C.; Laing, R.; Murdoch, D.R.; Chambers, S.T.; Vettise, D.; Tuffery, C.M.; Hancox, R.J. Biomarkers of Cardiac Dysfunction and Mortality from Community-Acquired Pneumonia in Adults. *PLoS ONE*
**2013**, 8, 1–7.
7. Nowak, A.; Breidthardt, T.; Christ-Crain, M.; Bingisser, R.; Meune, C.; Tanglay, Y.; Heinisch, C.; Reiter, M.; Drexler, B.; Arenja, N.; Twerenbold, R.; Stolz, D.; Tamm, M.; Muller, B.; Muller, C. Direct comparison of three natriuretic peptides for prediction of short- and long-term mortality in patients with community-acquired pneumonia. *Chest*
**2012**, *141*, 974–982.
8. Cangemi, R.; Casciaro, M.; Rossi, E.; Calvieri, C.; Bucci, T.; Calabrese, C.M.; Taliani, G.; Falcone, M.; Palange, P.; Bertazzoni, G.; Farcomeni, A.; Grieco, S.; Pignatelli, P.; Violi, F.; Albanese, F.; Biliotti, E.; Carnevale, R.; Catasca, E.; Celestini, A.; Esvan, R.; Fazi, L.; Marinelli, P.; Mordenti, M.; Napoleone, L.; Palumbo, M.; Pastori, D.; Perri, L.; Proietti, M.; Marco, R.C.; Russo, A.; et al. Platelet activation is associated with myocardial infarction in patients with pneumonia. *J. Am. Coll Cardiol*
**2014**, *64*, 1917–1925.
9. Méndez, R.; Aldás, I.; Amara, I.; Gimeno, A.; Posadas, T.; Reyes, S.; Suescun, M.; Alonso, R.; Menendez, R. Biomarkers As Predictors Of Early And Long-Term Cardiovascular Events In Community-Acquired Pneumonia. Preliminary Results. *Am J Respir Crit Care Med*
**2016**, *193*,2016:A2095

### 2.9. SESSION IX. OPTIMIZING ANTIMICROBIAL THERAPY

#### **2.9.1. Optimizing Antimicrobial Therapy: Role of New Antibiotics** 

NiedermanMichael S.M.D.Professor of Clinical Medicine, Weill Cornell Medical CollegeAssociate Chief, Division of Pulmonary and Critical Care Medicine, New York Presbyterian/Weill Cornell, Medical Center, New York, NY

New Antibiotics for Gram-Negative Pneumonia

- Ceftolozane–tazobactam
- Ceftazidime–avibactam
- Aztreonam–avibactam: active vs. metallo-β lactamases
- Siderophore cephalosporin
- Carbavance (carbapenem/BLI)
- Meropenem/RPX7009 (Vaborbactam): enhanced KPC activity
- Imipenem–relebactam
- Plazomicin
- POL 7080 (macrolide Lpt D inhibitor)
  – Vincent JL, et al. Crit Care 2016;20:133
- Cyclopeptide
  – Murepavadin: bactericidal vs. P. aeruginosa, incl. MDR pathogens. Targets bacterial outer membrane proteins, binds LPS. No clinical trials yet in VAP

New Antibiotics for Gram-Positive Pneumonia

- Oxazolidinones that are active vs. linezolid-resistant MRSA
  – Tedizolid: Lower MICs than linezolid vs. MRSA. Once daily dosing. No serotonergic agent interactions. SSTI trials done. HCAP/VAP study ongoing
  – Radezolid: concentrates in macrophages and neutrophils
- Glycopeptide/cephalosporin heterodimer (TD-1607). For serious Gram-positive infections, including VAP and bacteremia (phase I)
- Tetracyclines
  – Omadacycline: IV and oral for CAP (DRSP, S. aureus incl. MRSA, atypicals, some GNB). Low protein binding. Less GI toxicity than tigecycline. Comparable to linezolid in cSSTI. Few drug interactions, little potential for Clostridium difficile. Approved for CAP.

#### **2.9.2. When Not to Start Broad-Spectrum Antibiotics in the ICU** 

ChastreJeanMDFrom the Service de Médecine Intensive Réanimation, Institut de Cardiométabolismeet Nutrition (iCAN), Hôpital La Pitié–Salpêtrière, Sorbonne Université,Assistance Publique–Hôpitaux de Paris (APHP), Paris, FranceE-mail: jean.chastre@aphp.fr

The rapid emergence and dissemination of antibiotic-resistant microorganisms in intensive care units (ICUs) worldwide threatens adequate antibiotic coverage of infected patients in this environment and may justify using regimens combining several broad-spectrum antibiotics antimicrobials, even when the presumed infection probability is low. Numerous studies have indeed documented that a significant increase in mortality is observed when optimal antibiotic therapy is delayed in infected ICU patients and some quality improvement initiatives that encouraged earlier prescribing have also reported decreases in mortality [1]. Unfortunately, this “spiraling empirical” practice increasingly leads to undue antibiotic administration to many ICU patients without true infections or with infections caused by very susceptible bacteria not requiring broad-spectrum antimicrobial agents, paradoxically driving the emergence of more antibiotic-resistant microorganisms and causing infections that are, in turn, associated with heightened mortality and morbidity. Receipt of unnecessary and prolonged broad-spectrum antibiotics can also cause significant direct harm including antibiotic-associated adverse events, *Clostridioides difficile* infections, and changes to the digestive microbiome [2]. 

Although the Surviving Sepsis Campaign guidelines [3] recommend starting new antibiotics within one hour in ICU patients with sepsis, such an approach leads to unnecessary treatment in many non-infected patients and thus could be unwarranted in many cases [4]. In a quasi-experimental, before-and-after, observational cohort study on patients admitted to the University of Virginia surgical ICU, Hranjec and colleagues documented that delaying antibiotics antimicrobials for hemodynamically stable patients with suspected infections until they were objectively documented was associated with more initially appropriate therapy and lower all-cause mortality than using an aggressive strategy [5]. Thus, for clinically stable patients, this strategy might achieve better antibiotic use without impacting prognosis. Patients with mildly or moderately severe, early-onset infections and no specific risk factors (e.g., prolonged hospitalization, immunosuppression and/or recent prolonged antibiotics) can also receive narrow-spectrum drugs, like a non-pseudomonal third-generation cephalosporin, as recommended in the ERS/ESICM/ESCMID/ALAT guidelines for the management of hospital-acquired pneumonia [6].

Obtaining specimens for appropriate cultures before antibiotic administration is essential to confirm infection, identify responsible pathogen(s) and enable therapy de-escalation in response to susceptibility profile(s). Having current and frequently updated knowledge of local bacteriological epidemiology increases the likelihood of prescribing appropriate initial antibiotics. Whether surveillance cultures could further improve empirical treatment selection for ICU patients with suspected hospital-acquired infections is still debated but should certainly be weighed when difficult-to-treat microorganisms abound, making initial choices particularly risky. 

For ICU patients admitted with community-acquired or healthcare-associated infections, more restraints for antimicrobial-therapy selection are certainly possible. Available data suggest that the incidence of pathogens resistant to the usual in-patient IDSA–ATS guideline-recommended antibiotic regimen for pneumonia (i.e., a non-pseudomonal cephalosporin and a macrolide) is usually not significantly increased unless two, three or more risk factors are present, with prior antibiotic use or hospitalization and poor functional status being more important predictors of resistant bacteria than nursing-home residence alone. Using such an algorithm could lead to fewer pneumonia patients unnecessarily receiving broad-spectrum antibiotics [7].

Within the past decade, the way clinical microbiology laboratories identify microorganisms was revolutionized, leaving behind slow, traditional methods based on phenotype characteristics (e.g., growth on defined media, colony morphology, Gram-staining, and biochemical reactions) incurring significant diagnosis delay, in exchange for new diagnostic techniques including real-time multiplex polymerase chain reaction and matrix-assisted laser-desorption ionization–time-of-flight mass spectrometry. The latter, making possible rapid pathogen identification and their antimicrobial-resistance patterns (at least for certain organisms), could undoubtedly promote earlier therapy appropriateness and de-escalation [8]. 

Regardless of the diagnostic strategy used for suspected infections in the ICU, serial clinical and microbiological evaluations are highly relevant to re-assess therapy after 48–72 h and stop it if infection is unlikely [9]. For many ICU patients with infections, including late-onset infections, therapy can be de-escalated, once respiratory tract-, blood- and/or other specimen-culture results become available, if no resistant organism is recovered or because the isolated pathogen is sensitive to a narrower-spectrum antibiotic than that prescribed empirically. For example, if MRSA is not found, vancomycin and linezolid should be stopped, unless the patient is allergic to β-lactams or has developed an infection with Gram-positive bacteria susceptible only to them. Unfortunately, study results showed that, despite not being associated with any adverse outcomes, de-escalation was not consistently applied in many ICUs [10].

The two most commonly cited reasons to prescribe combined antibiotics for the entire treatment duration are to achieve synergy and prevent resistant-strain emergence. However, antibiotic synergy has only been shown to be valuable in vitro and in patients with neutropenia or a >25% probability of death. Randomized–controlled trial results on combined therapy showed its benefit to be inconsistent or null, even when they were pooled in meta-analyses or analysis was restricted to *P. aeruginosa*-infected patients. Importantly, such regimens did not prevent antimicrobial-resistance emergence under therapy, and were associated with significantly more nephrotoxicity. Based on those data, most patients’ therapy could be safely switched to monotherapy after 3–5 days, provided that the initial therapy was appropriate, the clinical course evolved favorably, and that microbiological data did not indicate very difficult-to-treat microorganisms, as can be observed for some non-fermenting GNB and carbapenemase-producing Enterobacteriaceae.

Computerized decision-support programs linked to electronic patient records can facilitate the dissemination of information to physicians for immediate use in therapeutic decision-making and improving quality of care. Partially or non-automated protocols, often instigated by hospital-based quality-improvement teams, also had demonstrated efficacy, as well as having an infectious disease specialist interacting regularly with the medical ICU team [11]. 

In summary, although no one disputes the fact that ICU patients with true bacterial infection and septic shock should receive appropriate antibiotics immediately, it is certainly possible to titrate the timing of antibiotic administration and not to use broad-spectrum agents in all cases, according to the risk of infection of patients, risk factors for MDR pathogens and severity of the disease [12]. 

References

1. Seymour, C.W.; Gesten, F.; Prescott, H.C.; et al. Time to Treatment and Mortality during Mandated Emergency Care for Sepsis. *N. Engl. J. Med*. **2017**, *376*, 2235–2244.
2. Routy, B.; Le Chatelier, E.; Derosa, L.; et al. Gut microbiome influences efficacy of PD-1-based immunotherapy against epithelial tumors. *Science*
**2018**, *359*, 91–97.
3. Rhodes, A.; Evans, L.E.; Alhazzani, W.; et al. Surviving Sepsis Campaign: International Guidelines for Management of Sepsis and Septic Shock: 2016. *Crit. Care Med*. **2017**, *45*, 486–552.
4. Klein Klouwenberg, P.M.; Cremer, O.L.; van Vught, L.A.; et al. Likelihood of infection in patients with presumed sepsis at the time of intensive care unit admission: A cohort study. *Crit. Care*
**2015**, 19, 319.
5. Hranjec, T.; Rosenberger, L.H.; Swenson, B.; et al. Aggressive versus conservative initiation of antimicrobial treatment in critically ill surgical patients with suspected intensive-care-unit-acquired infection: A quasi-experimental, before and after observational cohort study. *Lancet Infect. Dis*. **2012,** 12, 774–780.
6. Torres, A.; Niederman, M.S.; Chastre, J.; et al. International ERS/ESICM/ESCMID/ALAT guidelines for the management of hospital-acquired pneumonia and ventilator-associated pneumonia. *Eur. Respir. J*. **2017**, *50*; doi:10.1183/13993003.00582-2017.
7. Wunderink, R.G. Community-acquired pneumonia versus healthcare-associated pneumonia. The returning pendulum. *Am J Respir Crit Care Med*. **2013**, *188*, 896–898.
8. Huang, A.M.; Newton, D.; Kunapuli, A.; et al. Impact of rapid organism identification via matrix-assisted laser desorption/ionization time-of-flight combined with antimicrobial stewardship team intervention in adult patients with bacteremia and candidemia. *Clin. Infect. Dis*
**2013**, *57*, 1237–1245.
9. Palacios-Baena, Z.R.; Delgado-Valverde, M.; Valiente Méndez, A.; et al. Impact of de-escalation on prognosis of patients with bacteraemia due to Enterobacteriaceae: a post-hoc analysis from a multicenter prospective cohort. *Clin Infect Dis.*
**2018** Dec 8. [Epub ahead of print].
10. Liu, P.; Ohl, C.; Johnson, J.; et al. Frequency of empiric antibiotic de-escalation in an acute care hospital with an established Antimicrobial Stewardship Program. *BMC Infect. Dis*. **2016***, 16*, 751.
11. Jenkins, T.C.; Price, C.S.; Sabel, A.L.; et al. Impact of routine infectious diseases service consultation on the evaluation, management, and outcomes of *Staphylococcus aureus* bacteremia. *Clin. Infect. Dis.*
**2018**, *46*, 1000–1008.
12. Klompas, M.; Calandra, T.; Singer, M. Antibiotics for sepsis-Finding the Equilibrium. *JAMA*
**2018**, *320,* 1433–1434.

#### **2.9.3. Prolonged Infusion of Beta-Lactams: Do We Have Enough Data?** 

WAELEJan J. DEDept. Of Critical Care Medicine, Ghent University Hospital, Ghent, Belgium

Broad-spectrum beta-lactam antibiotics are the cornerstone of antimicrobial therapy—both empirical and directed—for many patients in the intensive care unit (ICU), because of their large spectrum covering a wide range of pathogens, low toxicity and cost.

In recent years, there has been an increased interest in a PKDP optimized use of antibiotics and particularly for beta-lactam antibiotics research into optimized therapy has been intense. The primary reason for this has been the extensive body of literature demonstrating the variability of antibiotic concentrations in critically ill patients with standard dosing [1] (both dose and method of administration) and the clinical failure that is often observed despite presumed antibiotic efficacy. Furthermore, an increase in antibiotic resistance around the world has resulted in a search for innovative strategies to treat infections caused by pathogens with reduced susceptibility—and on occasions overt resistant pathogens, and for methods to reduce the development of resistance during routine antibiotic therapy. Prolonged infusion of beta-lactam antibiotics has been proposed to overcome all of this and improve outcome, treat multidrug resistant (MDR) infections and reduce resistance.

Prolonged infusion covers different strategies; both extended infusion (infusion of an antibiotic dose over 2 h or more), and continuous infusion. Although both are often lumped together and may serve the same goal, they should not be used interchangeably. Most of the data currently available are on continuous infusion, and the results should not be extrapolated to extended infusion.

The key feature of prolonged infusion is that it avoids the peaks and troughs observed in intermittent infusion and it prolongs the time during which a concentration is above a certain threshold [2]. Considering that antibiotic efficacy of beta-lactam antibiotics is determined by the time above the minimal inhibitory concentrations of the causative pathogen, and that up to 100% time above this concentration is associated with increased bacterial killing, it should be evident that prolonged infusion results in a more effective antibiotic therapy.

The data on prolonged infusion can be divided in studies that have investigated the pharmacokinetics and studies that have looked at clinical outcomes. The literature documenting the changed PK is quite consistent and shows that indeed PK is changed, and antibiotic exposure is higher with prolonged infusion [2]. One limitation is that for higher targets (to treat more resistant pathogens), time above the MIC may be (paradoxically reduced) with continuous infusion.

The clinical data on prolonged infusion are less clear. A number of studies have suggested a clinical advantage of continuous infusion, and when put together in a meta-analysis a reduced mortality has even been described [3], but the most solid data on the topic could not demonstrate superiority of continuous infusion, but it can be said that prolonged infusion seems to be safe, and not associated with worse outcomes. A large study (the Beta-Lactam Infusion Group-III study (BLING-III)), aiming to include 7000 patients in Australia, UK, Belgium, New Zealand, Sweden, France and Portugal, has recently started and should give a definitive answer to this important question.

A few reports have described the use of prolonged infusion to treat MDR infections, but these are purely descriptive, and the role of the infusion strategy is unclear.

Data on the effect on antimicrobial resistance are less clear and the available data so far could not demonstrate an impact on the development of antibiotic resistant in patients with continuous infusion, but this is limited by the low quality of the evidence [4].

A number of practical issues and potential pitfalls should be considered before embarking on continuous infusion. Antibiotic stability is key, and not all antibiotics are suitable for prolonged infusion [5]. Most commonly used broad spectrum beta-lactam antibiotics are stable for a number of hours but rarely beyond 12 h at room temperature; the involvement of a clinical pharmacist in this process is recommended. Continuous infusion requires a separate infusion line, and this may pose practical problems; for extended infusion this may be less a challenge. A loading dose is required before starting continuous infusion and should not be forgotten. For extended infusion, care should be taken to use infusion lines with small dead spaces to avoid antibiotic solution remaining behind in the infusion line. Finally, prolonged infusion should not be used for economic reasons—similar target attainment with lower doses.

Some areas of uncertainty remain. For some patients just changing the infusion strategy may be insufficient to reach the set target, and it is not clear if the PK target changes when switching to continuous infusion.

In conclusion, prolonged infusion of beta-lactam antibiotics (in fact continuous infusion) holds promise as it has been shown to lead to better PK target attainment. The effect on clinical outcomes is less clear, and although the intervention is cheap and relatively simple to implement, there is no compelling evidence to consider intermittent infusion of beta-lactam antibiotics obsolete.

References

1. Roberts, J.A.; Paul, S.K.; Akova, M.; Bassetti, M.; De Waele, J.J.; Dimopoulos, G.; Kaukonen, K.M.; Koulenti, D.; Martin, C.; Montravers, P.; Rello, J.; Rhodes, A.; Starr, T.; Wallis, S.C.; Lipman, J., DALI Study. DALI: Defining antibiotic levels in intensive care unit patients: are current β-lactam antibiotic doses sufficient for critically ill patients. *Clin. Infect. Dis*. **2014**, *58*, 1072–1083.
2. Roberts, J.A.; Paratz, J.; Paratz, E.; Krueger, W.A.; Lipman, J. Continuous infusion of beta-lactam antibiotics in severe infections: a review of its role. *Int. J. Antimicrob. Agents*
**2007**, *30*, 11–18.
3. Roberts, J.A.; Abdul-Aziz, M.H.; Davis, J.S.; Dulhunty, J.M.; Cotta, M.O.; Myburgh, J.; Bellomo, R.; Lipman, J. Continuous versus Intermittent β-Lactam Infusion in Severe Sepsis. A Meta-analysis of Individual Patient Data from Randomized Trials. *Am. J. Respir. Crit. Care Med*. **2016**, *194*, 681–691.
4. Dhaese, S.A.M.; De Kezel, M.; Callant, M.; Boelens, J.; De Bus, L.; Depuydt, P.; De Waele, J.J. Emergence of antimicrobial resistance to piperacillin/tazobactam or meropenem in the ICU: Intermittent versus continuous infusion. A retrospective cohort study. *J. Crit. Care*. **2018**, *47*, 164-168.
5. De Waele, J.J.; Lipman, J.; Carlier, M.; Roberts, J.A. Subtleties in practical application of prolonged infusion of β-lactam antibiotics. *Int. J. Antimicrob. Agents*
**2015**, *45*, 461–463.

### 2.10. Posters Presentations

#### **2.10.1. The Relation Between Arterial Hyperoxia And Mortality In Septic Shock Patients** 

ElsayedSamar Elsayed Fahmy^1^ElsayedHany Eid Mohamed^2^ZaytounTayseer Mohamed^3^1Master of Critical Care medicine, Department of Critical Care medicine—Faculty of Medicine—Alexandria University2Lecturer of Critical Care medicine, Department of Critical Care medicine—Faculty of Medicine—Alexandria University3Professor of Critical Care Medicine, Department of Critical Care medicine—Faculty of Medicine—Alexandria University

**Introduction:** Sepsis remains the primary cause of death in intensive care unit (ICU) patients despite improvements in antibiotic and early hemodynamic management. Sepsis is defined as life-threatening organ dysfunction caused by a dysregulated host response to infection. Septic shock is defined as a subset of sepsis in which underlying circulatory and cellular metabolism abnormalities are profound enough to substantially increase mortality. Oxygen must be regarded as any other drug with potential dose and time depended side effects. Oxygen administration is the most widely prescribed therapy in critically ill patients and frequently represents a life-saving intervention. Since hypoxemia is generally viewed as deleterious and moderate levels of arterial hyperoxia as benign, health care practitioners are more likely to accept supranormal arterial O2 levels as this provides a wider safety buffer. The aim of this work was to investigate whether hyperoxia is associated with higher mortality in septic shock patients.

**Objective:** The aim of this work was to investigate whether hyperoxia is associated with higher mortality in septic shock patients. 

**Methods:** This study was carried out on 200 septic shock patients. After fulfilling the inclusion criteria, the following data were collected and recorded: The demographic data, Clinical data, Severity scoring systems, Clinical and microbiological cause of sepsis, Laboratory investigations, Ventilatory data, Oxygenation status data, days of mechanical ventilation, length of hospital stay and the patient outcome . The studied patients were classified in the end of the study into two groups: group I (the non hyperoxic group) that included 40 patients who were exposed to PaO_2_ <120 mmHg and group II (the hyperoxic group) that included 160 patients who were exposed to PaO_2_ >120 mmHg. We divided the group II into two subgroups, a group of moderate hyperoxia which included 118 patients who were exposed to PaO_2_ 120–300 and a group of severe hyperoxia which included 42 patients who were exposed to PaO_2_ > 300.

**Results:** In group I: mean number of days of mechanical ventilation was of 5.25 ± 1.81 day, while in group II: mean number was of 11.38 ± 5.27 day. In group I: mean number of days of ICU stay was of 8.88 ± 3.31 day, while in group II: mean number was of 13.14 ± 5.39 day. In group I: mean number of days of hospital stay was of 11.18 ± 3.74 day while in group II: mean number was of 13.55 ± 5.11 day. In group I, 23 patients (57.5%) were discharged and 17 patients (10.6%) were died. While in group II, 17 patients (42.5%) were discharged and 143 patients (89.4%) were died. In subgroup of moderate hyperoxaemia, 16 patients (94.1%) were discharged and 102 patients (71.3%) were died. While in subgroup of severe hyperoxaemia, 1 patient (5.9%) was discharged and 41 patients(28.7%) were died. In patients exposed to 1 day of hyperoxia (a total of 16 patients), 4 patients (23.5%) were discharged and 12 patients (8.4%) were died. While in patients exposed to hyperoxia more than 1 day ( a total of 144 patients), 13 patients (76.5%) were discharged and 131 patients (91.6% ) were died. 

**Conclusion:** Hyperoxia was associated with increased number of mechanical ventilation days, length of ICU stay and hospital stay. Furthermore it was associated with increase in the mortality among septic shock patients. The severity of hyperoxia and the duration of exposure to hyperoxia could as well influence the prognosis in septic shock patients.

#### **2.10.2. Novel Stratergies to Treat Travel-Associated Multi-Resistant Gram-Negative Pneumonia Requring Icu Support Following In-House Laboratory Synergy Testing** 

ThomasStephanie^1^CullenMairi^1^HassanIbrahim^1^MirzaSajjad^1^TaylorMoira^1^FeltonTim^2^1Department of Clinical Microbiology, Manchester University Foundation Trust, (Wythenshawe Hospital), Manchester, UK2Department of Adult Intensive Care2, Manchester University Foundation Trust, (Wythenshawe Hospital), Manchester, UK

**Introduction**

Antibiotics are the ONLY class of drug where giving to one patient may change the efficacy in another patient. Resistance among the Gram-negative bacilli is increasing at an alarming rate. Antimicrobial resistance (AMR) is mobile via genes on plasmids, leading to spread through bacterial populations which is augmented by increasing global air travel and migration. The direct consequences of infection with resistant microorganisms include more severe, longer illnesses, increased mortality, prolonged hospital stay and increased healthcare costs. Of particular concern is carbapenem resistance in the Gram-negative Enterobacteriacae (Carbapenemase Producing Enterobacteriacea, CPE). There are few new antimicrobial agents in the pipeline which give cover for these Gram-negative pathogens. New agents are being developed to cover the serine carbapenemases (KPC, OXA), however very few agents are available to cover metallo-carbapenemases (NDM, VIM, IMI). Therefore, novel strategies to treat these infections must be employed. 

**Case report**

We report a case of severe pneumonia in a patient transferred directly by air (medevac), from Turkey to our Adult Intensive Care Unit. Respiratory samples (BAL) isolated multi-drug resistant Gram-negative bacilli. Both *Acinetobacter* spp. carrying an OXA-23 and OXA-51 and *Klebsiella pneumoniae* carrying an OXA-48 and NDM were detected. Both organisms were pan-resistant. In-house laboratory antimicrobial synergy testing was carried out to determine a treatment regime and a novel treatment combination was initiated (Figure 1).

**Conclusions**

The WHO states that antimicrobial resistance “threatens the core of modern medicine and sustainability of an effective, global public health response”. AMR results from systematic misuse and overuse of antibiotics. In view of the ease and amount of global travel, every nation is at risk. Carbapenem resistance in the Gram-negative population is increasing at an alarming rate. Very few new or replacement products are in the pipeline. Therefore novel strategies to treat these infections are required. Combinations of antimicrobials, based around individualised resistance mechanisms and laboratory synergy testing can be employed. 

#### **2.10.3. Human Metapneumovirus as a Cause of Severe Respiratory Failure in a Patient Requiring Extracorporeal Membrane Oxygenation (ECMO)** 

ThomasStephanieDr^1^Traverse-HealyLiam^2^TempletonRichardDr^3^GarciaMiguelDr^3^1Department of Microbiology2University of Manchester Medical School, Manchester, UK3Department of Cardiothoracic Critical Care, Wythenshawe Hospital, Manchester University NHS, Foundation Trust, Southmoor Road, Wythenshawe, UK

**Introduction:** Human Metapneumovirus (hMPV), from the paramyxovirus family, is frequently a cause of childhood respiratory tract infections but may rarely cause acute respiratory failure. hMPV can produce a spectrum of disease from mild coryza to bronchiolitis and pneumonia and is difficult to distinguish clinically from respiratory syncytial virus infection. Reinfection in adulthood is common and often asymptomatic but can occasionally be severe particularly in elderly or immunocompromised patients. We report a case of severe acute respiratory failure in a patient who had been newly diagnosed with pro-myeloid leukaemia, who subsequently required Extra Corporeal Membrane Oxygenation (ECMO), and where the only pathogen identified was hMPV.

**Case Report:** A previously fit and well 23-year-old woman presented to her local A+E with a two-week history of bruising and menorrhagia. She was diagnosed with pro-myeloid leukaemia for which she received 10 days of idarubicin and arsenic treatment. Post-therapy she became neutropenic and developed a chest infection. Multiple sets of blood cultures were taken along with a serum galactomannan all of which were negative. The only pathogen identified was hMPV, from PCR of a throat swab. She was started on a variety of anti-microbials including intravenous ribavirin directed against hMPV. Over the next four days the patient’s oxygen requirements increased, and she was moved to a level 2 bed for CPAP and the next day to ICU. She was intubated one day after arriving in ICU but remained hypoxic (Pa02 8–10 kPa) despite 90–95% oxygen. She was therefore referred for consideration of veno-venous (VV) ECMO support. She was accepted as a candidate for ECMO based on a Murray score of 9 and the potentially reversible aetiology of her acute respiratory failure. The patient was transferred and commenced on VV ECMO via femoro-femoral cannulation. A CT head on arrival showed a small left-sided bleed and some swelling though her pupils remained 2 mm and reactive. However, at 4 am the next morning her pupils were found to be 7 mm and fixed. A repeat CT head showed catastrophic bleeding and brain stem herniation. The decision was taken to remove care and she died later that day. 

**Discussion:** Testing for hMPV is part of the routine viral PCR panel but positive results may be disregarded in patients with acute respiratory failure particularly when another organism is identified concurrently. This is despite accumulating evidence that hMPV can cause severe respiratory disease and ARDS even in immunocompetent patients. There is a paucity of evidence to support the use of any drug therapies to treat hMPV although ribavirin and intravenous immunoglobulin have both shown an effect in vitro. As such, the mainstay of management is supportive care which can include the use of VV ECMO. VV ECMO provides gas exchange thereby increasing oxygenation while allowing the lungs to be rested from the insult caused by mechanical ventilation. Haemorrhage is the most common complication of ECMO due to associated clotting factor dilution, platelet dysfunction and the need for patients to be systemically anticoagulated. Here we report the case of patient who after cytotoxic chemotherapy developed a fulminant pneumonia and ARDS due to hMPV infection and required VV ECMO support. She then had a large intracranial haemorrhage secondary either to ECMO or sepsis-induced coagulopathy and likely complicated by her underlying haematological malignancy. 

**Conclusion:** In adult patients with acute respiratory failure hMPV infection should form part of the differential diagnosis. If hMPV is confirmed physicians should prepare for the possibility of rapid deterioration and the potential need for ECMO support which may itself cause significant complications.

#### **2.10.4. *Clostridium Difficile* in an Intensive Care Unit** 

OcaboP.Garcia-RayadoG.AsinM.EdrosoP.LozanoH.MatuteA.AbansesP.ArcheM. J.MayordomoC.HerreroS.CamonA.CruellasM.SanchezC.SeralC.Hospital Clínico Universitario Lozano Blesa

**Introduction:** Over the last years, there was an increase in the number and severity of Clostridium difficile infections (CDI) in all medical settings, including the intensive care unit (ICU). The current prevalence of CDI among ICU patients is estimated at 0.4-4% and has severe impact on morbidity and mortality.

**Objective:** The main objective was to know the incidence of C. Difficile in a third level medical- surgical ICU, likewise the characteristics of the infected patients.

The secondary objective was to study the association between the given treatment and the maintained clinical cure until the hospital discharge.

**Material and methods:** We used a retrospective, observational and analytical design. Have been included all patients with ICD with microbiological (+) tests diagnosed in the ICU from January 2014 to December 2017. Positive microbiological tests have been excluded within the period of antibiotic treatment and those with negative toxin or that could not be confirmed by PCR.

**Results:** 19 patients were included. The descriptive analysis shows us that 63,2% of the patients were men with a medium age of 61.9 years. 52.6% of infected patients were over 65 years. 

About comorbidities, diabetes mellitus was present in 31.6% of patients, acute kidney injury in 36.8% and 47.4 of them were immunodepressed.

About therapies, 94.7% was given antibiotic treatment before the infection by *Clostridium difficile*, 100% of the patients were receiving proton pump inhibitors.

It is striking that in the majority of the patients who were diagnosed with *Clostridium difficile* infection, the antibiotic treatment they were receiving previously could not be interrupted. In the analytical study it was observed that 100% of patients who stopped taking antibiotics at the time of diagnosis of Clostridium infection evolved more favorably than those who could not, as well as those patients who responded early (<5 days) to treatment with C. difficile had a greater maintained clinical cure.

Finally, treatment with metronidazole in monotherapy was associated, although not significantly, with a greater maintained clinical response than combined treatment with metronidazole + vancomycin, which could be explained by a severity bias.

**Conclusion:***Clostridium difficile* infection is more serious when affects a critical ill patient. It is mandatory to evaluate periodically the need to continue with the prescribed treatments (antibiotic therapy, proton pump inhibitors …), as well as an early diagnosis to identify those patients with higher risk of recurrence to choose the most appropriate antibiotic therapy for them.

#### **2.10.5. Tuberculosis Meningitis in Aragon** 

PueyoAna Maria Camon^1^BuilPaula Ocabo^2^JordanTina Herrero^3^PimentelLuisa Cabrera^3^CanalesMaria José Crussels^4^CondeIsabel Sanjoaquin^4^1Servicio de Medicina Interna. Hospital Clínico Universitario Lozano Blesa2Servicio de Medicina Intensiva. Hospital Clínico Universitario Lozano Blesa3Servicio de Neumologia. Hospital Clínico Universitario Lozano Blesa. Servicio de Enfermedades Infecciosas4Hospital Clínico Universitario Lozano Blesa

**Background:** Tuberculosis (TB) remains a significant public health problem worldwide, with an estimated 8.7 million cases and 1.4 million deaths from it in 2011. TBM the most fatal presentation of tuberculosis (TB) especially in HIV-infected patients is a real diagnostic and therapeutic challenge worldwide.

**Objective:** The aim of the studio is to determine epidemiological, clinical and microbiological characteristics of TB in the general population of North Region of Spain (Aragon) and also investigate those in TBM patients.

**Material/Methods:** This was a retrospective cohort study of adults TB patients (age ≥ 18 years) who were notified in Aragon from January 1st, 2011 to December 31st 3017. 1204 adults cases notified during the period. The threshold of significance was set at 5%. Statistical analysis was carried out by SPSS 22.0.

**Results:** The incidence of TB in the general population of Aragon has fallen by 42% from 2011 to 2017. The background country in 80% of the cases was Spain, and in 20% they came from Eastern Europe. The subtype with the highest incidence is respiratory TB. Mortality in the general population of Aragón is 5.4%, while in patients with HIV positive infection it is 13% (Figure 1).

The prevalence of Clinical University Hospital Lozano Blesa area TBM was 2.36%. The study population was predominantly female. The mean age of the study population was 45.57 ± 12.7 years. The most common symptoms were headache and altered mental status, without any other neurological defects. The duration of present symptoms may vary from 1 day to 4 months. Critical care specializes was involved in 33% of patients. 

All patients had a lumbar tap (LP) done for cerebrospinal fluid (CSF) analysis. Mycobacterium tuberculosis baciloscopy wasn t identify in any of the samples after Ziehl Nielson stain. CSF mycobacterial culture was positive in 55%. PCR of TB was positive in 33% of them. No abnormal radiological findings have been found. 

Risk factors such as HIV co infected patients were reported in 33% cases and malignancy in 11%. Two—thirds of TBM patients have a Spanish background, and rest of them were from Africa. No other risk factors have been observed. 

On followed up after 6 months of discharged patients 70% of them had a full recovery, however 2 patients lost follow up. 

**Conclusions**

- The incidence of TB in Aragón has dropped significantly between 2011 and 2017 and TBM was the first most common of extrapulmonary disease.
- TBM is a very critical diseases in terms of fatal outcome and permanent sequelae and requires rapid diagnoses and treatment.

#### **2.10.6. Pancreatic Stone Protein as Mortality Predictor in Critically Ill Patients With Sepsis** 

L.García de Guadiana-Romualdo^1^M. D.Albaladejo-Otón^1^S.Rebollo-Acebes^2^E.Jiménez-Santos^1^R.Jiménez-Sánchez^2^P.Esteban-Torrella^1^A.Ortín-Freire^2^J. M.Allegue-Gallego^2^M.Berger^3^F.Rebeaud^4^1Biochemistry Department. University Santa Lucia Hospital (Cartagena, Spain)2Critical Care Unit. University Santa Lucía Hospital (Cartagena, Spain)3Philips Handheld Diagnostics, Eindhoven, The Netherlands4Chief Scientific Officer of Abionic

**Introduction**

Despite significant improvements in clinical management, including antibiotic and early hemodynamic support, severe forms of infections, such as sepsis and septic shock, are the leading causes of mortality in intensive care unit (ICU) patients. Prognostication is currently done using scores systems, such as the Sequential Organ Failure Assessment (SOFA), based on clinical and analytical data as surrogates for organ dysfunction. However, although numerous biomarkers have been evaluated in sepsis, none has sufficient accuracy to be used in clinical practice. The most commonly used biomarker in septic patients is procalcitonin (PCT), but its main utility is to discontinue antibiotic therapy when concentrations falls. Recently, Surviving Sepsis Campaing has identified the research to create novel biomarkers in sepsis as one of the 26 priorities for sepsis and septic shock. 

**Objective**

In this study, we have analyzed the accuracy of pancreatic stone/regenerating protein (PSP) levels, measured on admission to Intensive Care Unit (ICU) and during the first 48 h of intensive care treatment, in predicting 28-day mortality, compared to that of lactate and PCT.

**Method**

Design and laboratory methods: Single center prospective observational study, enrolling patients admitted to the ICU with sepsis and septic shock, according to Sepsis-3 definition. On admission to ICU, lactate level was measured, by using Point of Care Testing analyzer, and SOFA score was calculated. Besides, blood samples for measurement on central laboratory of PCT (electrochemiluminiscence assay) and PSP (ELISA assay) were collected on admission and on day 2. 

Statistical analysis: The association between biomarkers and 28-day mortality was assessed by Cox-regression analysis and to evaluate the accuracy of the biomarkers for 28-day mortality, we used ROC curves to determine the AUC. The DeLong method was used for comparison of ROC AUCs and the optimal cutoffs were derived by the Youden index.

**Results**

A total of 122 consecutive adult patients median age: 65 years (Interquartil range (IQR): 52–72); 68 male (55.7%) admitted to the ICU with diagnosis of sepsis (52.5%) or septic shock (47.5%), 94 (77%) medical and 28 (23%) surgical patients, were enrolled, with a 28-day mortality rate of 27%. The most common infection source was of abdominal origin (32%) and bacteremia was documented in 47 (38.5%) patients. Infection was microbiologically documented in 89 (73.0%) patients, being gram negative bacteria the main cause of infection (52.1%).

#### **2.10.7. Sepsis Scores Underestimate Outcome in Sepsis Patients in the Emergency Department due to the Treatment Paradox** 

J. W.UffenH.van GoorJ. J.OosterheertM. J. A.de RegtH. A. H.KaasjagerUniversity Medical Center Utrecht, Utrecht, The Netherlands

**Introduction:** In absence of a gold standard for the diagnosis of sepsis, prognostic scores have been introduced to guide treatment decisions in the emergency department (ED). The quick Sequential Organ Failure Assessment (qSOFA) has been developed to identify non-intensive care unit patients with infection at risk of dying, which might be lowered by early interventions. However, qSOFA was developed and validated in large observational patient cohorts in which treatment was given. This introduces the risk of an epidemiological phenomenon called the treatment paradox: the effect of a strong predictor for mortality will be reduced if that predictor also acts as a trigger for initiating treatment to prevent mortality. If prognostic scores are developed in treated populations they will likely underestimate the risk of predicted outcomes in the absence of treatment. 

**Objectives:** the aim of this study is to investigate whether sepsis scores are influenced by the treatment paradox in patients with suspected infection in the ED in a large cohort from a tertiary hospital.

**Method:** data was used from the SPACE (sepsis in acutely ill patients in the ED) cohort, which prospectively registers data of internal medicine ED patients with suspected infection. Systemic Inflammatory Response Syndrome (SIRS) and qSOFA scores are automatically calculated and systematically recorded in patient records. To investigate the existence of a treatment paradox, we quantified the associations between worse prognostic scores (SIRS ≥ 2 and qSOFA ≥ 2) and the intensity of initial treatment by vasopressor use within 24 h, administration of antibiotics within 1 h and aggressive fluid resuscitation of more than 1 L in the first hour.

**Results:** 1700 consecutive patients (median age 61 ± IQR 50-72, 52% men) were included. 1046 (61.5%) had a SIRS score ≥2 and 124 (7.3%) had a qSOFA ≥2. Patients with SIRS ≥2 or qSOFA ≥2 received more intensive therapy then patients who scored negative on any of these scores. After correction for age and comorbidities, multiple logistic regression analysis showed qSOFA ≥2 was associated with vasopressor use (OR 8.3 [95% CI 4.4–15.5]) and antibiotic administration within 1 h (OR 6.6 [95% CI 3.6–12.2]). Both qSOFA ≥2 and SIRS ≥2 were associated with aggressive fluid resuscitation (OR 4.2 [95% CI 2.6–4.9]) and OR 2.1 [95% CI 1.5–3.0], respectively).

**Conclusions:** early sepsis treatment is more intensive in ED patients with a SIRS score or qSOFA ≥2, suggesting these scores trigger treatment decisions. Therefore, SIRS and qSOFA scores are likely to be effected by a treatment paradox and underestimate the risk of mortality in patients with suspected infection in the ED, imposing a risk of under treatment.

#### **2.10.8. Immunocompetent Versus Immunocompromised Patients With Respiratory Tract Infections in the Emergency Department: No Differences in Clinical Presentation** 

J. W.UffenA.GerristmaJ. J.OosterheertM. J. A.de RegtH. A. H.KaasjagerUniversity Medical Center Utrecht, Utrecht, The Netherlands

**Introduction:** immunosuppression due to medication or underlying illness leads to different immune responses to pathogenic micro-organisms in immunocompromised and immunocompetent patients. This may lead to different clinical presentations of common infectious diseases, such as respiratory tract infections. Furthermore, this introduces risks of misrecognition and under treatment of these infections. 

**Objectives:** the aim of this study is to determine differences and possible pitfalls in clinical presentation between immunocompromised and immunocompetent patients with respiratory tract infections in the emergency department (ED).

**Methods:** since September 2016, the SPACE (sepsis in acutely ill patients in the ED) cohort prospectively registers data of all internal medicine ED patients with suspected infection. Data on symptoms, vital signs, clinical manifestations and laboratory results upon admission to the ED are systematically collected. Patients are considered immunocompromised when well-defined criteria are fulfilled. Final diagnoses are systematically registered using predefined definitions based on microbiological, radiological and clinical criteria, independent of the working diagnosis at presentation. Consecutive patients between September 2016 and August 2018 with a final diagnosis of upper or lower respiratory tract infections were included, to analyze differences in clinical presentation at the ED between immunocompromised and immunocompetent patients.

**Results:** 600 consecutive patients with a final respiratory tract infection diagnosis (median age 62 ± IQR 51–70, 54% men) were included. 209 (35%) were considered immunocompromised. The main cause of immunodeficiency was corticosteroid use (*n* = 83, 40%), followed by immunosuppression therapy in context of solid organ transplant (*n* = 49, 23%). A respiratory virus was the main cause of respiratory infection (*n* = 278, 46%), equally distributed between immunocompromised and immunocompetent patients (*n* = 96, 46% respectively *n* = 181, 47%, *p* = 0.889) followed by a bacterial community acquired pneumonia (*n* = 266, 44%), also equally distributed (*n* = 101, 48% respectively *n* = 165, 42%, *p* = 0.166). Between immunocompromised and immunocompetent patients no significant differences were found in (duration of) symptoms at presentation, vital parameters, and findings at physical examination. Laboratory results differed significantly in leukocyte count (median 8.0 versus 9.1 × 10^9^/L respectively, *p* < 0.001) but not in CRP levels (median 55.0 versus 56.0mg/L respectively, *p* = 0.974).

**Conclusions:** in the ED, immunocompromised and immunocompetent patients with a respiratory tract infection have a similar presentation, based on vital signs, symptoms, findings at physical exam and even CRP levels.

#### **2.10.9. Functional Outcome in Patients with Sepsis and/or Septic Shock after Admission in the Intensive Care Unit (ICU)** 

RouxinolPedro^1^PiscalhoInês^2^TeixeiraJoana^2^CampelloGlória^3^PiresLígia^4^RitaMilene Santa^5^1Internist, Department of Intensive Care, Centro Hospitalar e Universitário do Algarve—Unidade de Portimão, Portimão, Portugal2Medical student, Department of Biomedical Sciences and Medicine, Universidade do Algarve, Portugal3Intensivist, Head of the Intensive Care Unit, Department of Intensive Care, Centro Hospitalar e Universitário do Algarve—Unidade de Portimão, Portimão, Portugal4Intensivist, Consultant in Pneumology, Department of Intensive Care, Centro Hospitalar e Universitário do Algarve—Unidade de Portimão, Portimão, Portugal5Nurse, Department of Intensive Care, Centro Hospitalar e Universitário do Algarve—Unidade de Portimão, Portimão, PortugalCorrespondence: Pedro Rouxinol; pedrouxinol@gmail.com

**Introduction:** Sepsis and septic shock are frequent diagnoses in intensive care units, either as an admission diagnosis or as a consequence of respiratory, abdominal, urinary and other health conditions.

The incidence of these diagnoses is high and cause a remarkable morbidity and mortality. Therefore, it is relevant to assess the functional outcome of patients after hospitalization in the ICU of “Centro Hospitalar e Universitário do Algarve (CHUA)—Unidade de Portimão”, with sepsis and/or septic shock, and to identify the variables that contribute to their functional recovery after discharge.

The background variables, which are relevant to characterize the patients and their clinical condition before hospitalization, as well as the ICU variables, were selected and used to establish an association with the data obtained in the intensive medicine follow-up consultation, carried out 6 months after ICU discharge. In order to monitor the functional outcome, these consultations focusing on the evaluation of: respiratory, neurological, digestive, muscular and psychological function.

**Objectives:** To characterize the functional outcome in critically ill patients with sepsis and/or septic shock admitted in the ICU and to identify variables that determine their recovery.

**Methods and results:** Patients admitted to the ICU of CHUA—Unidade de Portimão from 1 May 2017 to 30 April 2018 (*n* = 309) were identified, of whom 151 were diagnosed with sepsis and/or septic shock (as an admission diagnosis to the ICU or as a diagnosis associated with other pathologies).

These 151 patients were divided into two distinct groups, survivors from sepsis and/or septic shock (*n* = 100) and deceased during hospitalization (*n* = 51). In the survivors group, 48 patients were monitored 6 months after ICU discharge, in the intensive medicine follow-up consultation.

The functional status and health-related quality of life were established with the application of the Anxiety and Depression Scale, EQ-5D (EuroQol five-dimension) questionnaire, ICU-PTSS (post-traumatic stress symptoms) questionnaire and ICU memories questionnaire, and when considered relevant, respiratory, neurological, digestive and muscular function were assessed.

Follow-up data were associated with background variables, including patient’s sex, age, work status, and previous health status and with ICU variables, namely severity of disease at admission (measured by Simplified Acute Physiology Score II—SAPSII), Sequential Organ Failure Assessment score (SOFA score), admission category, duration of ICU stay, number of days of ventilation, number of days of antibiotic therapy.

**Conclusions:** This study is relevant and allows us to verify the impact of age and premorbid conditions in the functional outcome and health-related quality of life in critically ill patients who survived to the hospitalization in the ICU due to sepsis and/or septic shock.

#### **2.10.10. Very Old Patients Admitted to the ICU for Sepsis: A Multicenter Study** 

IbarzMercedes^1^BoumendilAriane^2^HaasLenneke E. M.^3^FlaattenHans^4^LangeDylan W. de^5^GuidetBertrand^6^ArtigasAntonioOn behalf of the VIP1 study^7^1Department of Intensive Care Medicine, University Hospital of Sagrado Corazón. Barcelona, Spain (mibarzvillamayor@gmail.com)2Assistance Publique-Hôpital de Paris, Hôpital Saint-Antoine, Service de Réanimation Médicale. Paris, F-75012, France (ariane.boumendil@gmail.com)3Department of Intensive Care Medicine, Diakonessenhuis Utrecht. Utrecht, the Netherlands (lvlelyveld@diakhuis.nl)4Department of Clinical Medicine, University of Bergen, Department of Anaesthesia and Intensive Care, Haukeland University Hospital. Bergen, Norway (hans.flaatten@uib.no)5Department of Intensive Care Medicine, University Medical Center, University of Utrecht. Utrecht, the Netherlands (d.w.delange@umcutrecht.nl)6(a) Assistance Publique—Hôpitaux de Paris, Hôpital Saint-Antoine, service de réanimation médicale. Paris, France (bertrand.guidet@aphp.fr)(b) Sorbonne Universités, UPMC Univ Paris 06, UMR_S 1136, Institut Pierre Louis d’Epidémiologie et de Santé Publique. Paris, France(c) INSERM, UMR_S 1136, Institut Pierre Louis d’Epidémiologie et de Santé Publique. Paris, France7(a) Department of Intensive Care Medicine, CIBER Enfermedades Respiratorias, Corporacion Sanitaria Universitaria Parc Tauli, Autonomous University of Barcelona. Sabadell, Spain (aartigas@tauli.cat)(b) Department of Intensive Care Medicine, University Hospitals Sagrat Cor and General de Catalunya. Quirón Salud. Barcelona-Sant Cugat, SpainCorrespondence: aartigas@tauli.cat; mibarzvillamayor@gmail.com

**Purpose:** The number of intensive care patients aged ≥80 years (VIPs) admitted for sepsis at ICUs is growing. VIPs have high mortality and morbidity, so the benefits of ICU admission are questioned. We aimed to identify ICU and 30-day mortality and secondly, risk factors associated with 30-day mortality in acute VIPs admitted for sepsis.

**Methods:** This prospective study included VIPs with SOFA scores ≥2 acutely admitted to 307 ICUs in 21 European countries. Sepsis was defined according to sepsis-3 definition. We described therapeutic interventions, limitations on life support, risk factors for mortality, and outcomes in VIPs admitted for sepsis.

**Results:** Of 3869 acutely admitted VIPs, 493 (12.7%) had sepsis as admission diagnosis. 53.8% were male, median age was 83 (IQR 81–86) years. Frailty (CFS > 4) was present in 51.1% and median SOFA score at admission was 9 (6–12). Therapeutic interventions registered were: vasoactive drugs [82.2%], mechanical ventilation (47.5%), non-invasive ventilation (21.9%) and renal replacement therapies (17.4%). Median ICU stay was 3.54 (IQR 1.5–8). Life-support limitations were present in 37.3% (only withhold 21.9%, withdraw ± withhold 15.4%). ICU mortality was 31.2% and 30-day mortality was 44.6%. Multivariate analysis (Cox) showed age, frailty, and SOFA score were the independent risk factors associated with 30-day mortality. 

**Conclusions:** Mortality in this population of acute VIPs admitted for sepsis was high. Frailty, older age and higher SOFA scores were independently associated with 30-day mortality. Future studies are needed to identify factors that can predict survival and quality of life after discharge from the ICU and the hospital.

Results of the Cox analysis in acute patients admitted for sepsis (*n* = 493)
HR (95% CI)*p*-valueAge (per five-year increase)1.33 (1.1–1.61)*p* = 0.0029Frailty: vulnerable vs. fit1.54 (1.02–2.34)*p* = 0.0416Frailty: frail vs. fit1.47 (1.07–2.02)*p* = 0.0182Male vs. female1.12 (0.85–1.47)*p* = 0.4202SOFA score (per one-point increase)1.13 (1.1–1.17)*p* < 0.0001

#### **2.10.11. Plasma Gelsolin Administration Decreases the Inflammatory Response in Mice with Sepsis** 

PiktelEwelina^1^WnorowskaUrszula^1^DurnaśBonita^2^BuckiRobert^1^1Department of Microbiological and Nanobiomedical Engineering, Medical University of Bialystok, Bialystok, 15-222 Mickiewicza 2C, Poland2Department of Microbiology and Immunology, The Faculty of Health Sciences of the Jan Kochanowski University in Kielce, Kielce, 25-317, Al. IX Wieków Kielc 19A, Poland

Gelsolin (GSN) is a multifunctional, actin-binding protein involved in a number of physiological and pathological processes such as cellular cytoskeleton remodeling (i.e., controlling of cell shape, chemotaxis, secretion) and those associated with continuous removal of actin filaments released from dead cells into the bloodstream as the results of cells injuries. Recent work has suggested that the extracellular isoform of GSN, called plasma gelsolin (pGSN), which is present in human blood and plasma and other extracellular fluids, including cerebrospinal fluid, airway surface fluid and wound fluid, might be a key factor involved in modulation of immune responses diminishing the inflammatory reaction of the host due to (i) privileged binding of microbial-derived compounds i.e., LPS from Gram-negative and LTA and Gram-positive bacteria and (ii) prevention of toll-like receptors (TLRs) activation. Nevertheless, there is a still limited knowledge about the pharmacokinetic parameters of exogenously administrated pGSN and implication of pGSN in reducing of inflammation in animals with sepsis, developed as a result of peritonitis upon bacterial injection into abdominal cavity.

The aim of the study was to assess pGSN biodistribution after intraperitoneal administration and to assess the protective effects of exogenous pGSN against *Pseudomonas aeruginosa* Xen5 strain challenge in the mouse model of peritonitis. 

Pharmacokinetic parameters of pGSN were analyzed on 10-week-old Cby.Cg-Foxn1nu/cmdb using the Odyssey Clx reader. pGSN (100 µL; 10 mg/mL) was administrated 8 h after peritonitis induction with the *P. aeruginosa* Xen5 injection. To evaluate the development of inflammation, a 100-µL volume of 2-deoxyglucose labeled with the fluorescent dye IRDye^®^800CW was injected intravenously. Antibacterial activity of pGSN was evaluated by counting the bacterial colonies and bacterial-derived chemiluminescence signal from the inoculation of the peritoneal fluid.

During the study, we observed decrease in tissue uptake of IRDye^®^800CW 2-deoxyglucose, indicative of inflammation severity, and decrease of bacterial outgrowth from ascites in mice subjected to intravenous pGSN injection. The greatest accumulation of dye-labeled GSN was observed in lungs and liver of pGSN-treated mice.

These observations indicate that administration of plasma gelsolin might prevent inflammatory response upon bacterial infection and might be used as a therapeutic approach in treatment of sepsis.

This work was financially supported by the National Science Centre, Poland (UMO-2015/17/B/NZ6/03473 to RB)

#### **2.10.12. Human Plasma Gelsolin Stimulates Phagocytosis in Mice Macrophages** 

WnorowskaUrszula^1^PiktelEwelina^1^DurnaśBonita^2^BuckiRobert^1^1Department of Microbiological and Nanobiomedical Engineering, Medical University of Bialystok, Bialystok, 15-222 Mickiewicza 2C, Poland2Department of Microbiology and Immunology, The Faculty of Health Sciences of the Jan Kochanowski University in Kielce, Kielce, 25-317, Al. IX Wieków Kielc 19A, Poland

Plasma gelsolin (pGSN) belongs to the family of actin-binding proteins (ABPs) that function in the blood as a part of an actin buffer and scavenger of different bioactive lipids including LPA, S1P, PAF and bacterial cell components LPS and LTA. pGSN is present in a variety of body fluids, including blood, plasma, urine, milk, cerebrospinal fluid and wound fluid. Effective phagocytosis by macrophages is crucial in the clearance of invading microbes. However, the effect of pGSN on phagocytosis process is still not investigated. In effect, we used a set of experiments to evaluate whether application of exogenous pGSN increases the bacteria phagocytosis. 

Mouse macrophages RAW 264.7 were seeded on round 12-mm collagen type I-coated cover glasses in a 24-well culture plate in RPMI 1640 medium and cultured in 5% CO2, 37ºC until confluent monolayers was formed. After that, pGSN (0, 2–10 µM) and *P. aeruginosa* Xen 5 (PA Xen 5) (5 × 105 CFU/mL) in varied combinations were added to obtain the ratio of bacteria to the studied macrophages as 10 to 1. Next, pGSN and PA Xen 5-treated macrophages were harvested, washed with PBS, fixed with methanol for 10 min, and stained using Giemsa protocol. The phagocytosis of PA Xen 5 by macrophages RAW 264.7 was observed using light microscopy. 

Our results indicate that administration of pGSN stimulates phagocytosis in dose-dependent manner, as evaluated by light microscopy. Preincubation of macrophages with pGSN (10 µM) enhances phagocytosis of PA Xen 5 (a 5-fold increase compared to control was observed). Moreover, 1 h preincubation of pGSN with macrophages followed by the addition of both pGSN with PA Xen 5, results in increase of the phagocytic to over 80%, even at low concentrations of gelsolin i.e., 0.2–2 µM. In contrast, there are no significant differences between phagocytosis of PA Xen 5 when macrophages were preincubated with bacteria, washed off and then pGSN for 1 h was added. Similarly, no beneficial effect was observed when cells were simultaneously treated with both pGSN and bacteria, without earlier pGSN preincubation. 

Above observations indicate that exogenous pGSN reinforce host antibacterial activity upon bacterial infection. Our results extend the previous findings on beneficial and protective effects of pGSN and suggest the possibility to employ pGSN-based therapies as effective approaches in the therapy of sepsis.

This work was financially supported by the National Science Centre, Poland (UMO-2015/17/B/NZ6/03473 to RB)

## Figures and Tables

**Figure 1:**
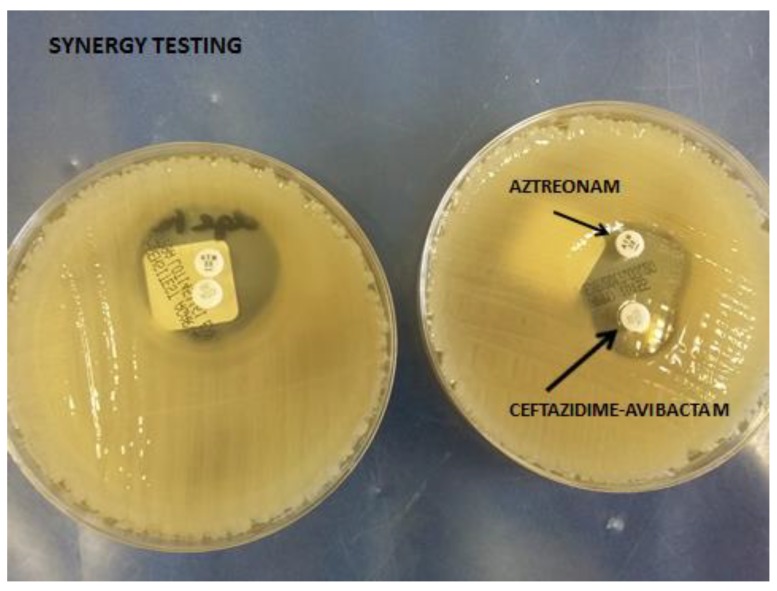
Positive Synergy Test. “Russian-doll” appearance implies synergy between Ceftazidime-avibactam and Aztreonam. When used in combination these agents can be used synergistically to cover both serine (Ceftazidine-avibactam) and metallo (Aztreonam) -carbapenemases.

**Figure 1:**
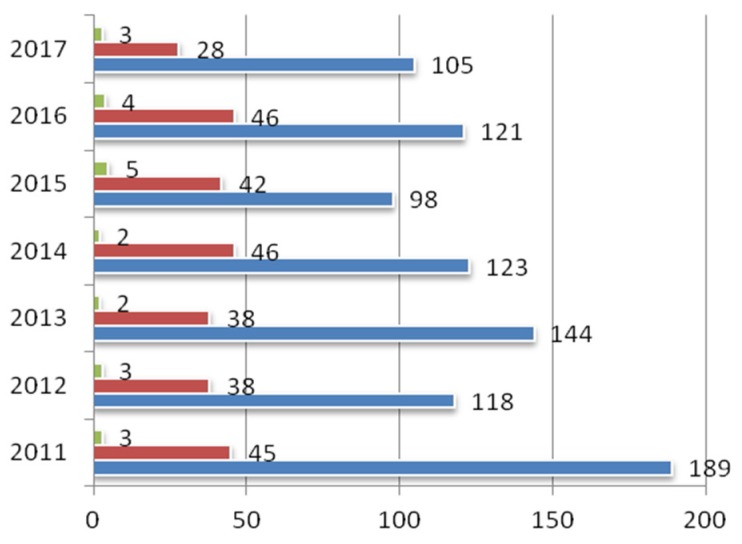
Types of TB in Aragon. Blue line: Respiratory TB. Red line: Other Tb. Green line: TB meningitis.

**Table 1:** Key features of **BETTER** RCTs in sepsis and septic shock compared to oncology RCTs that lead to **BETTER** outcomes.

Condition	Biomarker-guided (therapy examples)	Early (in time and disease progression) (examples)	antibioTic adjuvanTs	in ER
Sepsis/septic shock	Vasopressin Norepinephrine Angiotensin-II Corticosteroids PCSK9 inhibition CETP inhibition Weaning from ventilation	Vasopressin EGDT	PCSK9 inhibition CETP inhibition	Within 1–2 h recruitment in ER
Cancer	Expression levels (HER2 neu for Herceptin)	Early stage cancer	Chemotherapy Adjuvants- Immune therapies	clinics/offices (prior to need for hospitalization)

